# Supplementary material for: Targeting RSV-neutralizing B cell receptors with anti-idiotypic antibodies
Source: Cell Rep. 2024 Oct 8;43(10):114811. doi: 10.1016/j.celrep.2024.114811 (PMC11496930; doi:10.1016/j.celrep.2024.114811)
Supplement: Document S1. Figures S1–S7 and Tables S1 and S2 [file mmc1.pdf]

Cell Reports, Volume *43*

Supplemental information

Targeting RSV-neutralizing B cell receptors

with anti-idiotypic antibodies

Samuel C. Scharffenberger, Yu-Hsin Wan, Leah J. Homad, Gargi Kher, Austin M. Haynes, Bibhav Poudel, Irika R. Sinha, Nicholas Aldridge, Ayana Pai, Madeleine Bibby, Crystal B. Chhan, Amelia R. Davis, Zoe Moodie, Maria Belen Palacio, Amelia Escolano, M. Juliana McElrath, Jim Boonyaratanakornkit, Marie Pancera, and Andrew T. McGuire

A

```
                        10        20        30        40        50        60        70        80        90       100       110       120  Accession
IGHV3-21*01    EVQLVESGGGLVKPGGSLRLSCAASGFTFSSYSMNWVRQAPGKGLEWVSSISSSSSYIYYADSVKGRFTISRDNAKNSLYLQMNSLRAEDTAVYYCAR------------------------    Number
ADI-19425 HC   ..................................................................................................LGYCSGGSCH-FDYWGQGTLVTVSS   MG524063
ADI-14337 HC   ..................................................................................................EYYDSSGYTNWFDPWGQGTLVTVSS  MG524251
ADI-25532 HC   Q....Q............................................................................................---GSSSSWYYFDYWGQGTLVTVSS   MG524182
```

B

```
                        10        20        30        40        50        60        70        80        90       100       110  Accession
IGLV1-40*01    QSVLTQPPSVSGAPGQRVTISCTGSSSNIGAGYDVHWYQQLPGTAPKLLIYGNSNRPSGVPDRFSGSKSGTSASLAITGLQAEDEADYYCQSYDSSLSG-------------    Number
ADI-19425 LC   .P.................................................................................................FYVFGTGTKLTVL  MG524528
ADI-14337 LC   .P...............................................................................................A--WVFGGGTQLTVL  MG524647
ADI-25532 LC   ...V..............................................................................................--YVFGTGTKVTVL  MG524716
```

Figure S1: Alignment of unmutated VH3-21/VL1-40 mAbs used to generate anti-idiotypic hybridomas. Related to Figure 1. (**A**) Alignment of ADI-19425, ADI-14337, and ADI-25532[S1] heavy chains to germline VH3-21*01. (**B**) Alignment of ADI-19425, ADI-14337, and ADI-25532 light chains to germline VL1-40*01.

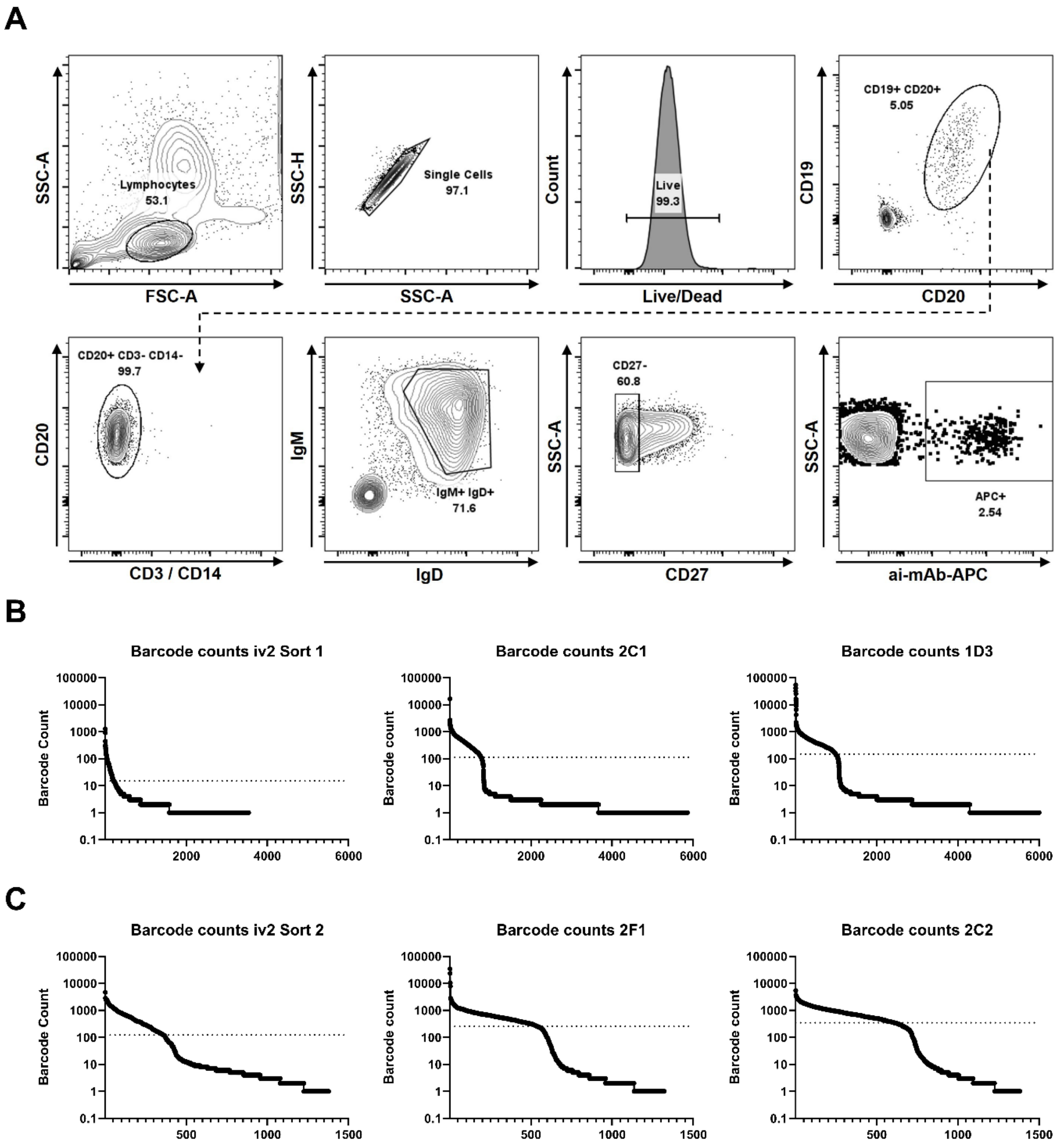

Figure S2: Gating strategy to sort ai-mAb specific naïve B cells and barcode threshold setting. Related to Figure 1. (**A**) Gating as follows: Single cells → Lymphocytes → Live cells → CD3$^-$ CD14$^-$ → CD19$^+$ CD20$^+$ → IgD$^+$ IgM$^+$ → ai-mAb-APC$^+$ B cells. Cell percentages of parent gate are shown, representative staining plots from 1 group of sorted cells. (**B & C**) ai-mAbs were labeled with APC conjugated to a unique oligonucleotide feature barcode and used to stain naïve PBMC in two cocktails containing iv2, 2C1, 1D3 (**B**) and iv2, 2F1, and 2C2 (**C**). Individual B cell libraries were generated using the chromium platform from 10X Genomics. Each panel shows the number of times the indicated feature barcode was observed for a given cell plotted from highest to lowest where each dot represents a single B cell. The thresholds, represented by the horizontal dotted line in each panel, were estimated by segmented generalized linear models using the *chngpt* R package[S2]. BCR sequences from B cells above

the threshold were considered positive for a given ai-mAb. B cells above the threshold from the iv2 isotype control in each sort were discarded from subsequent analysis.

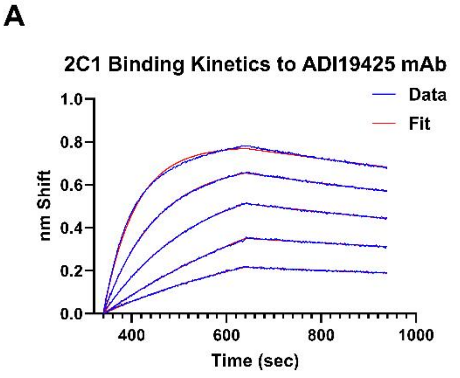

k_a(1/Ms)	k_a Error	k_d(1/s)	k_d Error	K_D (M)
2.92E+05	2.68E+03	4.42E-04	4.24E-06	**1.51E-09**

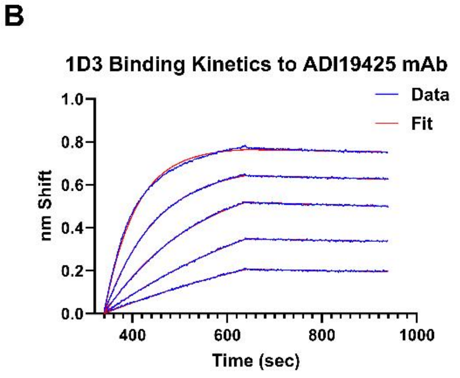

k_a(1/Ms)	k_a Error	k_d(1/s)	k_d Error	K_D (M)
3.07E+05	2.72E+03	1.15E-04	4.16E-06	**3.76E-10**

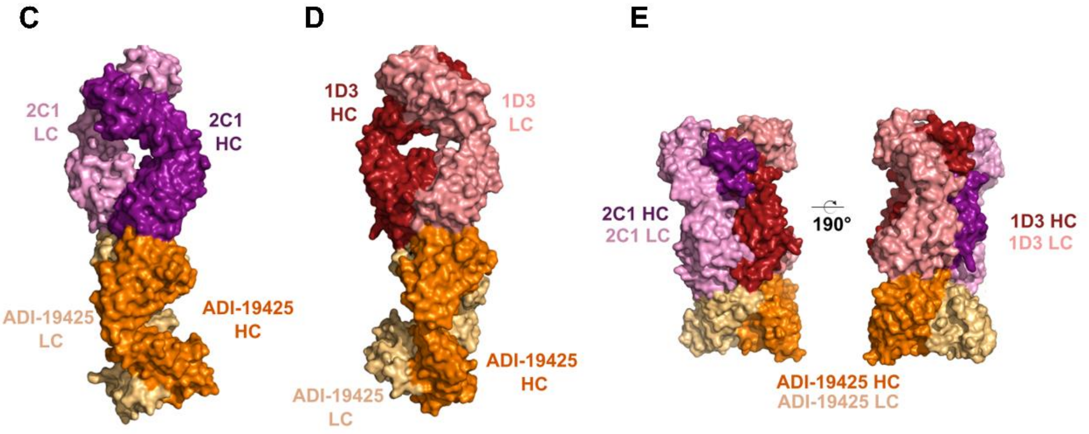

Figure S3: Binding kinetics of ai-mAbs to ADI-19425 Fab and Fab-Fab structures. Related to Figure 2. (**A** & **B**) Binding kinetics of recombinant 2C1 (**A**) or 1D3 (**B**) IgG to ADI-19425 Fab at the indicated concentrations were measured by biolayer interferometry. Blue lines represent observed data, and red lines are theoretical fit. The calculated kinetic parameters are shown below each plot. Data shown from a single biological replicate. (**C-E**) Full view of complexes between (**C**) 2C1 Fab (dark purple – heavy chain, light purple – light chain) and (**D**) 1D3 Fab (red – heavy chain, pink – light chain) with ADI-19425 Fab (orange – heavy chain, wheat – light chain). (**E**) Overlays of both complexes are shown aligned by the VH/VL region of ADI-19425 Fab and rotated on the y-axis by 190°.

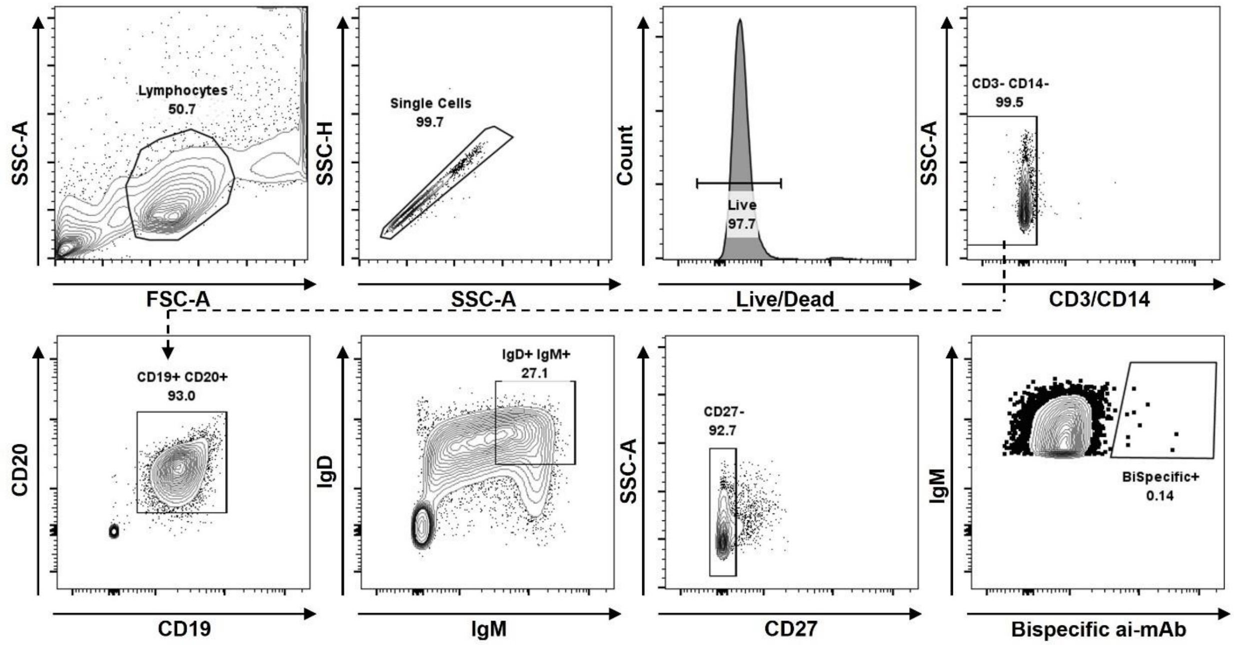

Figure S4: Gating strategy to identify and single cell sort ai-mAb specific naïve B cells. Related to Figure 4. Gating as follows: Lymphocytes → Single cells → Live cells → $CD3^-$, $CD14^-$ → $CD19^+$ $CD20^+$ → IgD^+ IgM^+ → $CD27^-$ → IgM^+ Bispecific ai-mAb^+. Percentages of parent gate indicated, representative gating from one sorting experiment.

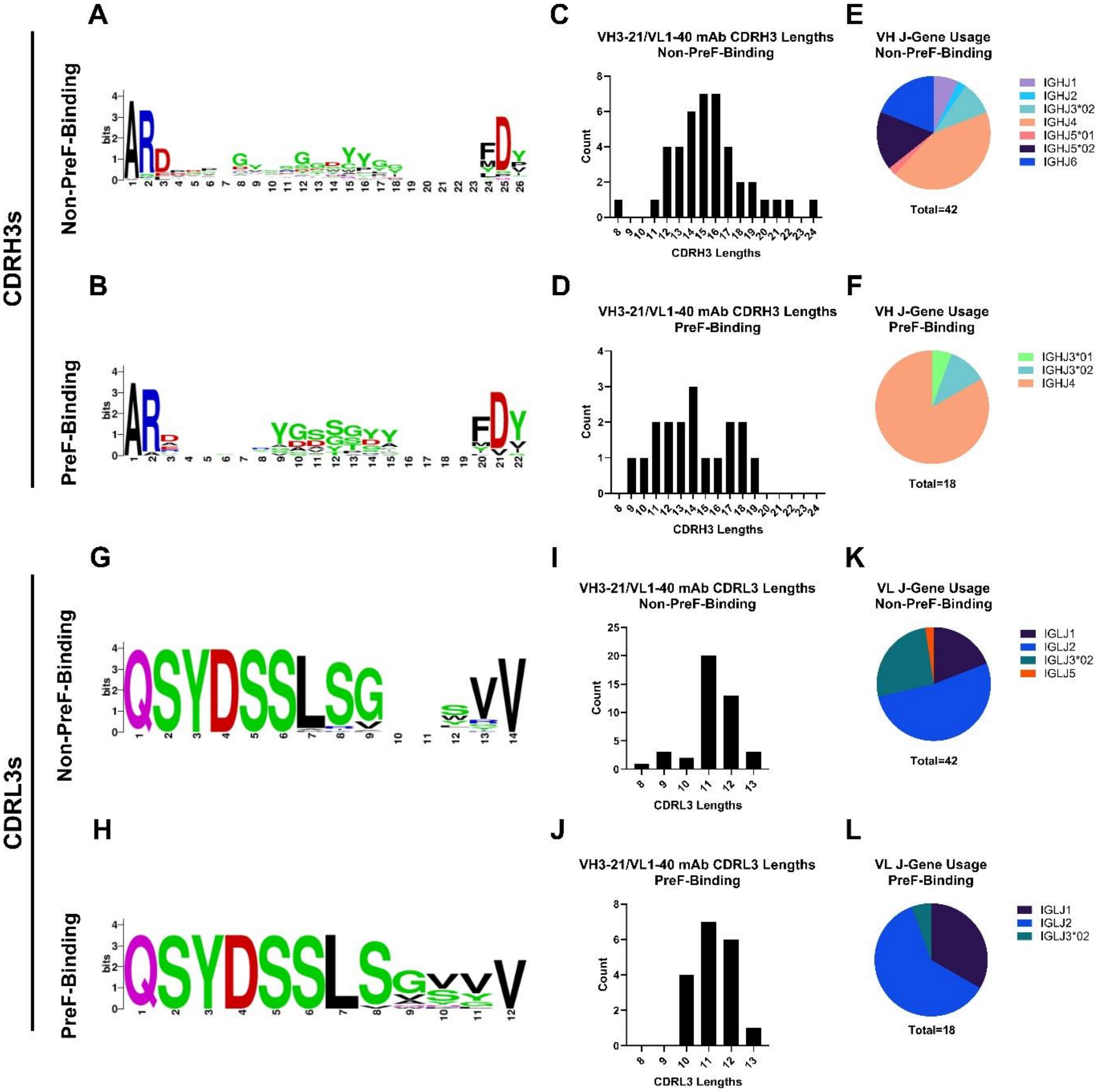

M

```
                 10
MLR_15     ARDD----YGDYGVVFDY
MLR_24     ARDPGYS-YGYGGYYFDY
MLR_55     ARDGYCSSTSCYDGGIDY
```

N

```
                 10
MLR_15     QSYDSSLSV--V
MLR_24     QSYDSSLSVNYV
MLR_55     QSYDSSLSV--V
```

Figure S5: CDR3 and J-gene analysis of ai-mAb-sorted VH3-21/VL1-40 mAbs. Related to Figure 4. (**A** & **B**) Sequence logo plots of non-preF-binding **A** and preF-binding **B** CDRH3s of sorted VH3-21/VL1-40 mAbs created with https://weblogo.berkeley.edu/logo.cgi. (**C** & **D**) Histograms of CDRH3 lengths of non-preF-binding **C** and preF-binding **D** VH3-21/VL1-40 mAbs. (**E** & **F**) Heavy chain J-gene usage of non-preF-binding **E** and preF-binding **F** VH3-21/VL1-40 mAbs. J-gene alleles with the same amino acid usage combined for analysis. (**G** & **H**) Sequence logo plots of non-preF-binding **G** and preF-binding **H** CDRL3s of sorted VH3-21/VL1-40 mAbs. (**I** & **J**) Histograms of CDRL3 lengths of non-preF-binding **I** and preF-binding **J** VH3-21/VL1-40 mAbs. J-gene alleles with the same amino acid usage combined for analysis (**K**

& **L**) Light chain J-gene usage of non-preF-binding **K** and preF-binding **L** VH3-21/VL1-40 B cells. (**M** & **N**) Alignments of CDRH3s **M** and CDRL3s **N** of RSV-neutralizing VH3-21/VL1-40 B cells.

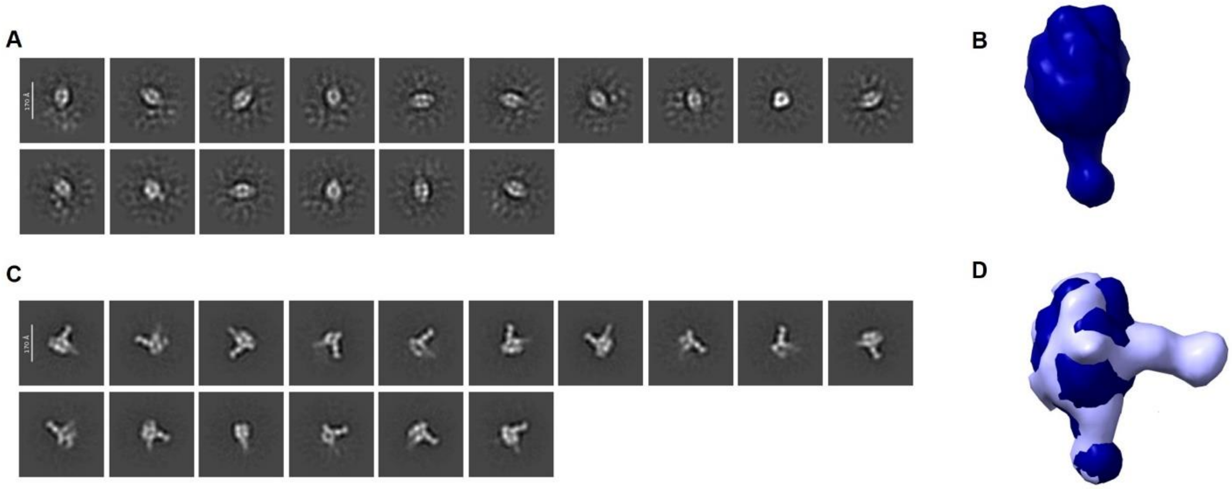

Figure S6: nsEM Images of DS-Cav1 and MLR_24 Fab – DS-Cav1 Complex. Related to Figure 4. (**A**) Representative 2D classes of unbound DS-Cav1[S3]. (**B**) A 20Å map of a crystal structure of RSV preF with a trimerization domain (PDB ID: 5TDG). (**C**) Representative 2D classes of the DS-Cav1 – MLR_24 Fab complex. (**D**) The 20Å map of the RSV preF crystal structure (dark blue) superimposed onto a representative 3D class of the DS-Cav1 – MLR_24 Fab complex (light purple).

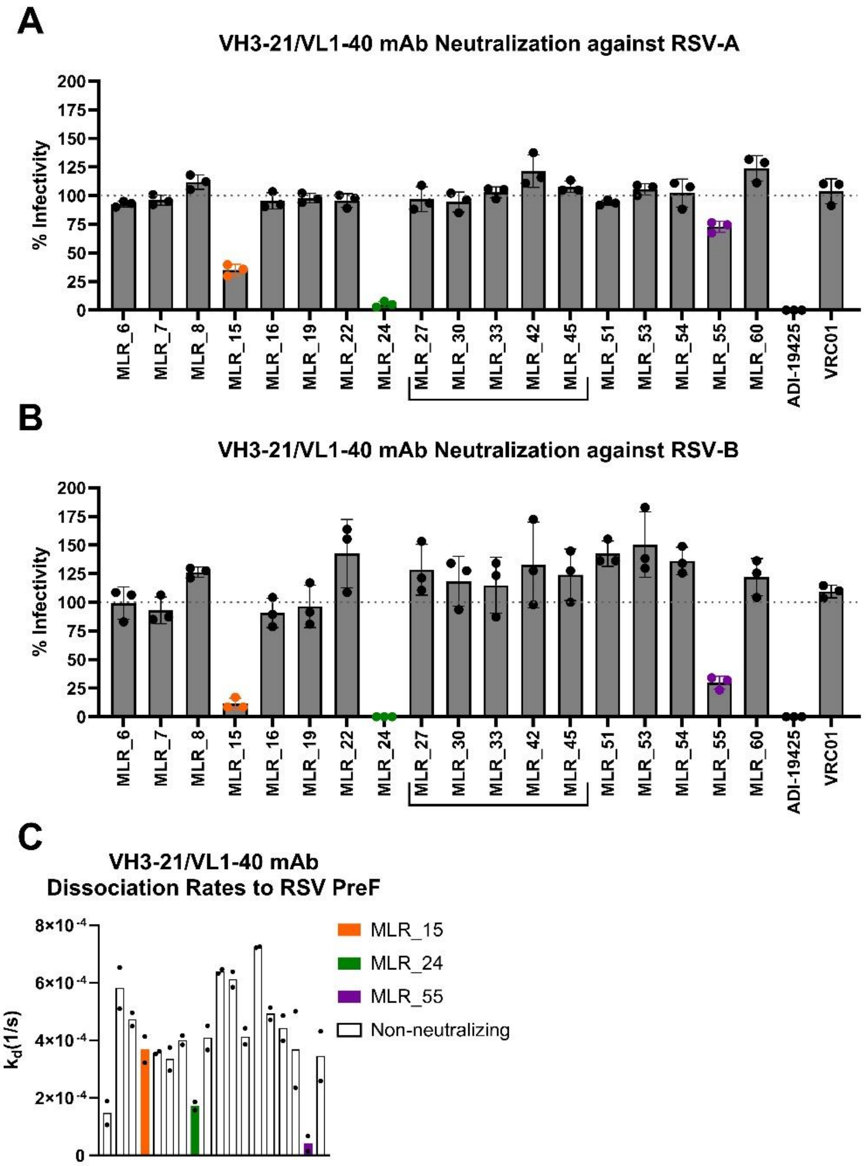

Figure S7: Neutralization screen of VH3-21/VL1-40 sorted mAbs against RSV-A / B and Steady State Affinity Measurements. Related to Figures 5 and 7**.** All recombinant VH3-21/VL1-40 preF-binding mAbs sorted with the bispecific ai-mAb were screened for neutralization activity against RSV-A (**A**) or RSV-B (**B**) at 500 µg/mL final dilution using a plaque reduction assay in triplicate. % Infectivity is the infectivity in the presence of mAb ÷ infectivity in the absence of mAb × 100%. Colored mAbs indicate those followed up for IC_{50} determination. VRC01[S4] is an irrelevant HIV mAb and included as a negative control. Bracketed mAbs were used in neutralization competition assays in **Fig. 7A & B**. Each point represents a technical replicate from a single experiment. Bars represent mean ± standard deviation. (**C**) Dissociation rates for VH3-21/VL1-40 mAbs averaged over the dilution series in **Fig. 5C**, coloring consistent as above. Each data point is is a biological replicate, bars indicate mean.

	ADI-19425 Fab - 1D3 Fab	ADI-19425 Fab - 2C1 Fab
**Data collection**		
Space group	P1	$P12_11$
Cell dimensions		
a, *b*, *c* (Å)	95.07, 149.98, 164.43	66.80, 66.93, 130.91
α, β, γ (°)	83.98, 89.90, 74.61	90, 103.74, 90
Resolution (Å)	49.45-2.72 (2.72 – 2.67)	50.00–2.44 (2.44-2.40)
R_{merge} [a]	0.116 (0.566)	0.242 (0.891)
<I/σ(I)>	4.0 (0.7)	15.4 (2.1)
$CC_{1/2}$	0.961 (0.319)	0.953 (0.844)
Completeness	92.5 (92.4)	98.4 (92.5)
Redundancy	1.9 (1.9)	6.6 (6.4)
**Refinement**		
Resolution (Å)	49.33 – 2.67 (2.77 – 2.67)	42.16 – 2.41 (2.50 – 2.41)
No. unique reflections	228772 (22653)	42798 (3991)
R_{work} [b] / R_{free} [c]	23.1/27.3 (32.6/37.2)	20.5/25.3 (26.9/33.1)
No. atoms	45020	6660
Protein	44852	6464
Water	178	166
Ligand	0	60
B-factors ($Å^2$)	56.24	34.79
Protein	56.31	34.80
Water	39.65	33.19
Ligand	0	41.86
RMS bond length (Å)	0.003	0.005
**Ramachadran Plot Statistics** [d]		
Residues	5948	861
Most Favored region	94.16	95.28
Allowed Region	5.48	4.37
Disallowed Region	0.36	0.35
Clashscore	6.10	6.66
**PDB ID**	8VS8	8VS7

[a] $R_{merge} = [\sum_h \sum_i |I_h - I_{hi}| / \sum_h \sum_i I_{hi}]$ where I_h is the mean of I_{hi} observations of reflection *h*. Numbers in parenthesis represent highest resolution shell. [b] R_{factor} and [c] $R_{free} = \sum||F_{obs}| - |F_{calc}|| / \sum|F_{obs}| \times 100$ for 95% of recorded data (R_{factor}) or 5% data (R_{free}). [d] Determined using MolProbity (10.1002/pro.3330)

Table S1: Data collection and refinement statistics for crystal structures

Name	Donor	RSV PreF Rmax (DS-Cav1)*	RSV-A Neut. IC_{50} (µg/mL)**	RSV-B Neut. IC_{50} (µg/mL)**	GenBank Accession Number (VH)	GenBank Accession Number (VL)
MLR_1	A	N.B.	N.N.	N.N.	PP429379	PP429439
MLR_2	A	N.B.	N.N.	N.N.	PP429380	PP429440
MLR_3	A	N.B.	N.N.	N.N.	PP429381	PP429441
MLR_4	A	N.B.	N.N.	N.N.	PP429382	PP429442
MLR_5	A	N.B.	N.N.	N.N.	PP429383	PP429443
MLR_6	A	0.311	N.N.	N.N.	PP429384	PP429444
MLR_7	A	0.834	N.N.	N.N.	PP429385	PP429445
MLR_8	A	0.437	N.N.	N.N.	PP429386	PP429446
MLR_9	A	N.B.	N.N.	N.N.	PP429387	PP429447
MLR_10	A	N.B.	N.N.	N.N.	PP429388	PP429448
MLR_11	A	N.B.	N.N.	N.N.	PP429389	PP429449
MLR_12	A	N.B.	N.N.	N.N.	PP429390	PP429450
MLR_13	A	N.B.	N.N.	N.N.	PP429391	PP429451
MLR_14	B	N.B.	N.N.	N.N.	PP429392	PP429452
MLR_15	B	1.017	130.57	21.75	PP429393	PP429453
MLR_16	B	0.333	N.N.	N.N.	PP429394	PP429454
MLR_17	B	N.B.	N.N.	N.N.	PP429395	PP429455
MLR_18	B	N.B.	N.N.	N.N.	PP429396	PP429456
MLR_19	B	0.465	N.N.	N.N.	PP429397	PP429457
MLR_20	B	N.B.	N.N.	N.N.	PP429398	PP429458
MLR_21	B	N.B.	N.N.	N.N.	PP429399	PP429459
MLR_22	B	0.303	N.N.	N.N.	PP429400	PP429460
MLR_23	B	N.B.	N.N.	N.N.	PP429401	PP429461
MLR_24	B	1.122	24.2	0.22	PP429402	PP429462
MLR_25	B	N.B.	N.N.	N.N.	PP429403	PP429463
MLR_26	B	N.B.	N.N.	N.N.	PP429404	PP429464
MLR_27	B	0.494	N.N.	N.N.	PP429405	PP429465
MLR_28	B	N.B.	N.N.	N.N.	PP429406	PP429466
MLR_29	B	N.B.	N.N.	N.N.	PP429407	PP429467
MLR_30	B	0.776	N.N.	N.N.	PP429408	PP429468
MLR_31	B	N.B.	N.N.	N.N.	PP429409	PP429469
MLR_32	B	N.B.	N.N.	N.N.	PP429410	PP429470
MLR_33	B	0.753	N.N.	N.N.	PP429411	PP429471
MLR_34	C	N.B.	N.N.	N.N.	PP429412	PP429472
MLR_35	C	N.B.	N.N.	N.N.	PP429413	PP429473
MLR_36	C	N.B.	N.N.	N.N.	PP429414	PP429474
MLR_37	C	N.B.	N.N.	N.N.	PP429415	PP429475
MLR_38	C	N.B.	N.N.	N.N.	PP429416	PP429476
MLR_39	C	N.B.	N.N.	N.N.	PP429417	PP429477
MLR_40	C	N.B.	N.N.	N.N.	PP429418	PP429478
MLR_41	C	N.B.	N.N.	N.N.	PP429419	PP429479
MLR_42	C	0.362	N.N.	N.N.	PP429420	PP429480
MLR_43	C	N.B.	N.N.	N.N.	PP429421	PP429481
MLR_44	C	N.B.	N.N.	N.N.	PP429422	PP429482
MLR_45	C	0.882	N.N.	N.N.	PP429423	PP429483
MLR_46	C	N.B.	N.N.	N.N.	PP429424	PP429484
MLR_47	C	N.B.	N.N.	N.N.	PP429425	PP429485
MLR_48	C	N.B.	N.N.	N.N.	PP429426	PP429486
MLR_49	C	N.B.	N.N.	N.N.	PP429427	PP429487
MLR_50	C	N.B.	N.N.	N.N.	PP429428	PP429488
MLR_51	C	0.500	N.N.	N.N.	PP429429	PP429489

MLR_52	C	N.B.	N.N.	N.N.	PP429430	PP429490
MLR_53	C	0.381	N.N.	N.N.	PP429431	PP429491
MLR_54	C	0.430	N.N.	N.N.	PP429432	PP429492
MLR_55	C	0.606	N.N.	98.21	PP429433	PP429493
MLR_56	C	N.B.	N.N.	N.N.	PP429434	PP429494
MLR_57	C	N.B.	N.N.	N.N.	PP429435	PP429495
MLR_58	C	N.B.	N.N.	N.N.	PP429436	PP429496
MLR_59	C	N.B.	N.N.	N.N.	PP429437	PP429497
MLR_60	C	0.426	N.N.	N.N.	PP429438	PP429498
*N.B., non-binder; **N.N. non-neutralizing.						

Table S2: Properties and accession numbers for sorted and produced VH3-21/VL1-40 mAbs

Supplemental References

1. Goodwin, E., Gilman, M.S.A., Wrapp, D., Chen, M., Ngwuta, J.O., Moin, S.M., Bai, P., Sivasubramanian, A., Connor, R.I., Wright, P.F., et al. (2018). Infants Infected with Respiratory Syncytial Virus Generate Potent Neutralizing Antibodies that Lack Somatic Hypermutation. Immunity *48*, 339-349.e335. 10.1016/j.immuni.2018.01.005.
2. Fong, Y., Huang, Y., Gilbert, P.B., and Permar, S.R. (2017). chngpt: threshold regression model estimation and inference. BMC Bioinformatics *18*, 454. 10.1186/s12859-017-1863-x.
3. McLellan, J.S., Chen, M., Joyce, M.G., Sastry, M., Stewart-Jones, G.B., Yang, Y., Zhang, B., Chen, L., Srivatsan, S., Zheng, A., et al. (2013). Structure-based design of a fusion glycoprotein vaccine for respiratory syncytial virus. Science *342*, 592-598. 10.1126/science.1243283.
4. Wu, X., Yang, Z.Y., Li, Y., Hogerkorp, C.M., Schief, W.R., Seaman, M.S., Zhou, T., Schmidt, S.D., Wu, L., Xu, L., et al. (2010). Rational design of envelope identifies broadly neutralizing human monoclonal antibodies to HIV-1. Science *329*, 856-861. 10.1126/science.1187659.
