# Supplementary material for: Targeting RSV-neutralizing B cell receptors with anti-idiotypic antibodies
Source: Cell Rep. 2024 Oct 8;43(10):114811. doi: 10.1016/j.celrep.2024.114811 (PMC11496930; doi:10.1016/j.celrep.2024.114811)
Supplement: Document S2. Article plus supplemental information [file mmc2.pdf]

Cell Reports

Article

Targeting RSV-neutralizing B cell receptors with anti-idiotypic antibodies

Graphical abstract

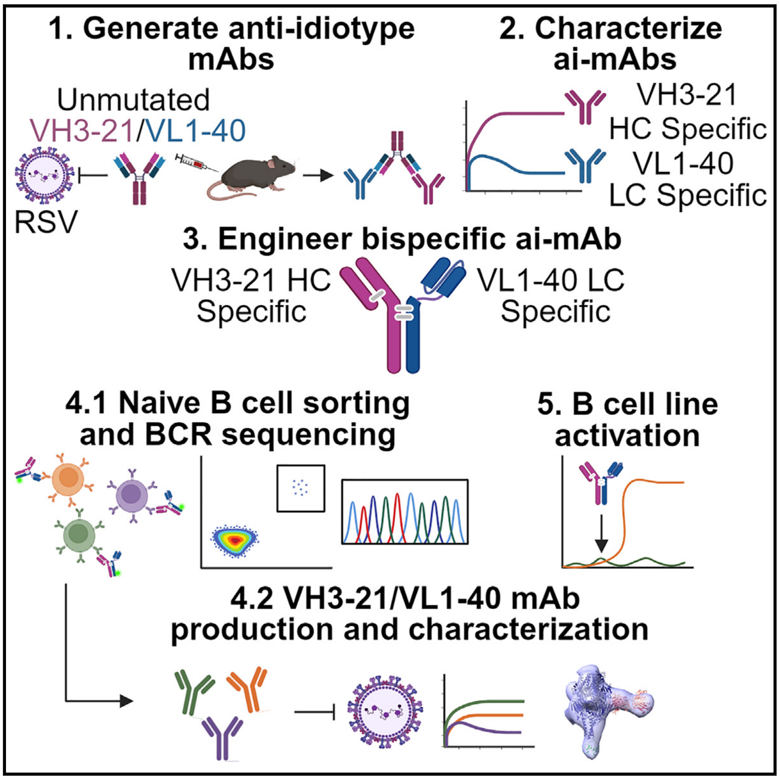

Authors

Samuel C. Scharffenberger, Yu-Hsin Wan, Leah J. Homad, ..., Jim Boonyaratanakornkit, Marie Pancera, Andrew T. McGuire

Correspondence

amcguire@fredhutch.org

In brief

Respiratory syncytial virus (RSV) is a leading cause of infant hospitalization and mortality with no approved vaccine for this population. Here, Scharffenberger et al. engineer a bispecific anti-idiotypic antibody-based immunogen to selectively target and activate B cells expressing unmutated VH3-21/VL1-40 BCRs hardwired to neutralize RSV.

Highlights

- Antibodies derived from VH3-21/VL1-40 gene paring are hardwired to neutralize RSV
- Developed a bispecific anti-idiotypic antibody (ai-mAb) to target VH3-21/VL1-40 BCRs
- ai-mAb specifically engages B cells encoding RSV-neutralizing antibodies
- ai-mAb selectively activates VH3-21/VL1-40 BCR$^+$ cell lines

Scharffenberger et al., 2024, Cell Reports *43*, 114811
October 22, 2024 © 2024 The Author(s). Published by Elsevier Inc.
https://doi.org/10.1016/j.celrep.2024.114811

CellPress

Cell Reports

CellPress
OPEN ACCESS

Article

Targeting RSV-neutralizing B cell receptors with anti-idiotypic antibodies

Samuel C. Scharffenberger,[1,2] Yu-Hsin Wan,[1] Leah J. Homad,[1] Gargi Kher,[1] Austin M. Haynes,[3] Bibhav Poudel,[1] Irika R. Sinha,[1] Nicholas Aldridge,[1] Ayana Pai,[1] Madeleine Bibby,[1] Crystal B. Chhan,[1,3] Amelia R. Davis,[1] Zoe Moodie,[1] Maria Belen Palacio,[4] Amelia Escolano,[4] M. Juliana McElrath,[1,5] Jim Boonyaratanakornkit,[1,3,5] Marie Pancera,[1] and Andrew T. McGuire[1,2,3,6,*]

[1]Vaccine and Infectious Disease Division, Fred Hutchinson Cancer Center, Seattle, WA 98109, USA
[2]Department of Laboratory Medicine and Pathology, University of Washington, Seattle, WA 98195, USA
[3]Department of Global Health, University of Washington, Seattle, WA 98195, USA
[4]Vaccine and Immunotherapy Center, Wistar Institute, Philadelphia, PA 19104, USA
[5]Department of Medicine, University of Washington, Seattle, WA 98195, USA
[6]Lead contact
*Correspondence: amcguire@fredhutch.org
https://doi.org/10.1016/j.celrep.2024.114811

SUMMARY

Respiratory syncytial virus (RSV) causes lower respiratory tract infections with significant morbidity and mortality at the extremes of age. Vaccines based on the viral fusion protein are approved for adults over 60, but infant protection relies on passive immunity via antibody transfer or maternal vaccination. An infant vaccine that rapidly elicits protective antibodies would fulfill a critical unmet need. Antibodies arising from the VH3-21/VL1-40 gene pairing can neutralize RSV without the need for affinity maturation, making them attractive to target through vaccination. Here, we develop an anti-idiotypic monoclonal antibody (ai-mAb) immunogen that is specific for unmutated VH3-21/VL1-40 B cell receptors (BCRs). The ai-mAb efficiently engages B cells with *bona fide* target BCRs and does not activate off-target non-neutralizing B cells, unlike recombinant pre-fusion (preF) protein used in current RSV vaccines. These results establish proof of concept for using an ai-mAb-derived vaccine to target B cells hardwired to produce RSV-neutralizing antibodies.

INTRODUCTION

Respiratory syncytial virus (RSV) is a common seasonal pathogen that generally causes mild respiratory symptoms in adults but can cause serious lower respiratory tract infection in infants and the elderly. RSV is responsible for ~60,000 recorded in-hospital deaths annually in children under 5[1,2] and accounts for a substantial hospitalization burden in infants as well as aged adults.[1,3,4] In 2023, two RSV vaccines, AREXVY (GSK) and ABRYSVO (Pfizer), were approved for use in adults over 60 years of age. Both are protein subunit vaccines based on the viral fusion protein (F). F can adopt a metastable pre-fusion (preF) state and a highly stable post-fusion state (postF). Most neutralizing antibodies in the serum of infected individuals map to preF, highlighting its importance for vaccine design.[5,6] Epitope mapping of anti-F monoclonal antibodies (mAbs) has identified distinct antigenic sites termed Ø–V. Some sites, such as Ø, III, and V, are only present on preF, while the others are present on both conformations.[5,7–14] Advances in antigen engineering have led to the development of stabilized preF immunogens,[15] which form the basis for AREXVY and ABRYSVO.[16]

Despite these important advances in RSV vaccine development, a vaccine for use in infants remains a critical unmet need. Prophylaxis for infants currently relies on passive immunity. Since 1998, the site II-directed mAb palivizumab, under the commercial name of SYNAGIS (Sobi), has been available for high-risk infants.[17,18] More recently, the extended half-life site Ø-specific mAb nirsevimab, under the commercial name Beyfortus (Sanofi), has been approved for all infants under 8 months of age entering their first RSV season.[10,19]

As an alternative prophylactic strategy, ABRYSVO is approved for use in pregnant individuals in the final month of gestation with the goal of protecting infants from birth through the first 6 months of age through transplacental transfer of protective antibodies.[16,20] Prophylactic strategies afford transient protection against lower respiratory tract disease in infants but are imperfect solutions because protection wanes as these antibodies are cleared. ABRYSVO is only administered after 32 weeks of gestation, providing a short window for implementation, and this timing may not be congruent with the seasonal pattern of RSV transmission. High costs prohibit the widespread use of passively transferred mAbs, particularly in low- and middle-income countries. While these prophylactic strategies are welcome tools for the prevention of RSV in the infant population, the development of an active vaccine that can rapidly establish durable protection in infants and toddlers could overcome the limitations of passive immunity.

© 2024 The Author(s). Published by Elsevier Inc.
This is an open access article under the CC BY license (http://creativecommons.org/licenses/by/4.0/).

Prevention of severe RSV infection in infants with palivizumab and nirsevimab suggests that neutralizing antibodies targeting F are important for protection against RSV-mediated disease in infants and indicates that a vaccine should seek to elicit high neutralizing antibody titers. However, raising protective antibodies in infants has historically been fraught with challenges. The earliest RSV vaccine was based on formalin-inactivated whole virus in which F preferentially adopted the postF conformation.[21] Unfortunately, this vaccine formulation led to enhanced respiratory disease in infants that required hospitalization and, in some cases, led to death.[22] This manifestation has been attributed in part to non-neutralizing anti-F antibodies.[21–23] Due to this failure, infant RSV vaccine research has proceeded cautiously.

In addition to issues with formulation, infant vaccination faces unique immunological challenges. For example, the infant immune system is inefficient at generating somatically mutated antibodies,[24,25] which are often required for high-affinity binding and/or neutralizing potency.[26] Moreover, maternal antibodies can interfere with *de novo* immune responses in infants. Maternal antibodies can bind to vaccine antigens and suppress the infant response to immunization, which can further complicate vaccination strategies.[27,28] Indeed, infants tend to elicit higher neutralizing titers following RSV infection when maternal antibody levels are low.[29,30]

Characterization of mAbs elicited by RSV infection in infants has provided critical insight into their humoral responses. The isolation of F-reactive mAbs from infected infants[9] has identified a class of potently neutralizing mAbs derived from the same antibody variable heavy (VH)- and variable light (VL)-chain genes: VH3-21 and VL1-40.[9] These VH3-21/VL1-40 mAbs mapped to an epitope on antigenic site III present only on preF[9,11] and remarkably, were able to potently neutralize RSV without undergoing affinity maturation.

The crystal structure of an unmutated neutralizing VH3-21/VL1-40 mAb in complex with preF revealed that the heavy-chain (HC) contacts are primarily made with complementarity-determining HC region 1 (CDRH1) and CDRH2, which are chromosomally encoded by VH3-21.[9] Similarly, the light chain (LC) contacts are made by complementarity-determining LC region 1 (CDRL1) and CDRL2 encoded by the VL1-40 gene. In addition to infected infants, B cells expressing F-reactive VH3-21/VL1-40 B cell receptors (BCRs) were also found in cord blood and in naive B cells from healthy adults.[9] Collectively these data indicate that unmutated VH3-21/VL1-40-derived antibodies are a public clonotype that is pre-configured to bind to and neutralize RSV, establishing them as an attractive, ubiquitous vaccine target.

We have demonstrated previously that vaccine antigens derived from anti-idiotypic mAbs (ai-mAbs) raised against unmutated precursor versions of neutralizing antibodies against HIV-1 and influenza could activate B cells *in vitro* and generate robust on-target B cell and serum responses using knockin mice *in vivo*.[31–35] Given that germline-encoded VH3-21/VL1-40 antibodies are preconfigured to neutralize RSV without the need for somatic mutation, we reasoned that they may be readily elicited by an anti-idiotype vaccine. To this end, we raised ai-mAbs by immunizing mice with preF-reactive, unmutated VH3-21/VL1-40 RSV-neutralizing mAbs. By integrating binding, B cell sorting, and structural analysis, we identified two ai-mAb candidates. One bound to VH3-21 HCs, and the other bound to VL1-40 LCs, both with high affinity and specificity. From these, we developed a bispecific ai-mAb that combined the binding properties of both. The bispecific ai-mAb was able to select for B cells capable of producing RSV-neutralizing antibodies from a diverse pool of naive B cells *ex vivo* and activate B cells engineered to express relevant target BCRs *in vitro.* These results provide proof of concept that an anti-idiotype vaccine has the potential to elicit RSV-neutralizing antibodies by specifically targeting and activating B cells expressing VH3-21/VL1-40 BCRs that are hardwired to neutralize RSV.

RESULTS

Isolation of monoclonal anti-idiotypic antibodies against mAbs encoded by VH3-21 and VL1-40 genes

Hybridomas were generated from mice immunized with a cocktail of three RSV F site III-directed neutralizing mAbs derived from unmutated VH3-21 and VL1-40 genes: ADI-19425, ADI-25532, and ADI-14337[9] (Figure S1). Four hybridoma supernatants (2C1, 2C2, 2F1, and 1D3) showed binding to the mAbs used to immunize and weaker or no binding to negative control mAbs. ai-mAbs were purified and evaluated for their ability to bind to a panel of recombinant mAbs by ELISA (Figure 1A). The panel included ADI-19425, ADI-25532, and ADI-14337 as well as chimeric antibodies with a VH3-21-encoded HC paired with a non-VL1-40 LC or a VL1-40-encoded LC but a non-VH3-21-derived HC and 37 other anti-Epstein-Barr virus (EBV) and anti-HIV-1 mAbs with diverse VH and VL gene usage.[33–36] All four ai-mAbs bound to ADI-19425, ADI-25532, and ADI-14337. 2C2, 2C1, and 2F1 bound to chimeric mAbs with a VL1-40 LC, while 1D3 bound to chimeric mAbs with a VH3-21 HC (Figure 1A). None of the ai-mAbs bound to any of the control antibodies (Figure 1A).

Fluorescently labeled ai-mAbs engage B cells expressing VH3-21- or VL1-40-derived BCRs in pools of naive B cells

We next sought to determine whether the ai-mAbs secreted by the hybridomas could recognize naive B cells expressing *bona fide* VH3-21/VL1-40 BCRs. 2C1, 2C2, 2F1, and 1D3 ai-mAbs were biotinylated and individually complexed with streptavidin-allophycocyanin (SA-APC) conjugated to a unique oligonucleotide barcode. To counter-screen against B cells that bind outside of the anti-idiotype paratope, we included a biotinylated isotype control, iv2,[35] conjugated to SA-APC with a unique oligonucleotide barcode. 2%–3% of naive B cells stained with each labeled ai-mAb, while <0.5% of B cells stained with the isotype control (Figure 1B). APC^+ naive B cells were sorted, and single-cell variable-diversity-joining (VDJ) antigen-barcode libraries were generated using paired HC/LC next-generation sequencing. The paired BCR sequences of cells sorted by each ai-mAb were identified by bioinformatic demultiplexing of the libraries (Figures S2B and S2C).[38] The percentage of ai-mAb sorted B cells that expressed VH3-21/VL1-40 pairs ranged from ~0.9% to ~5% (Figure 1C), which is marginally higher than would be expected by chance (0.1%–0.35%) based on high-throughput unbiased sequencing of paired BCRs from antigen-inexperienced B cells.[37]

CellPress
OPEN ACCESS

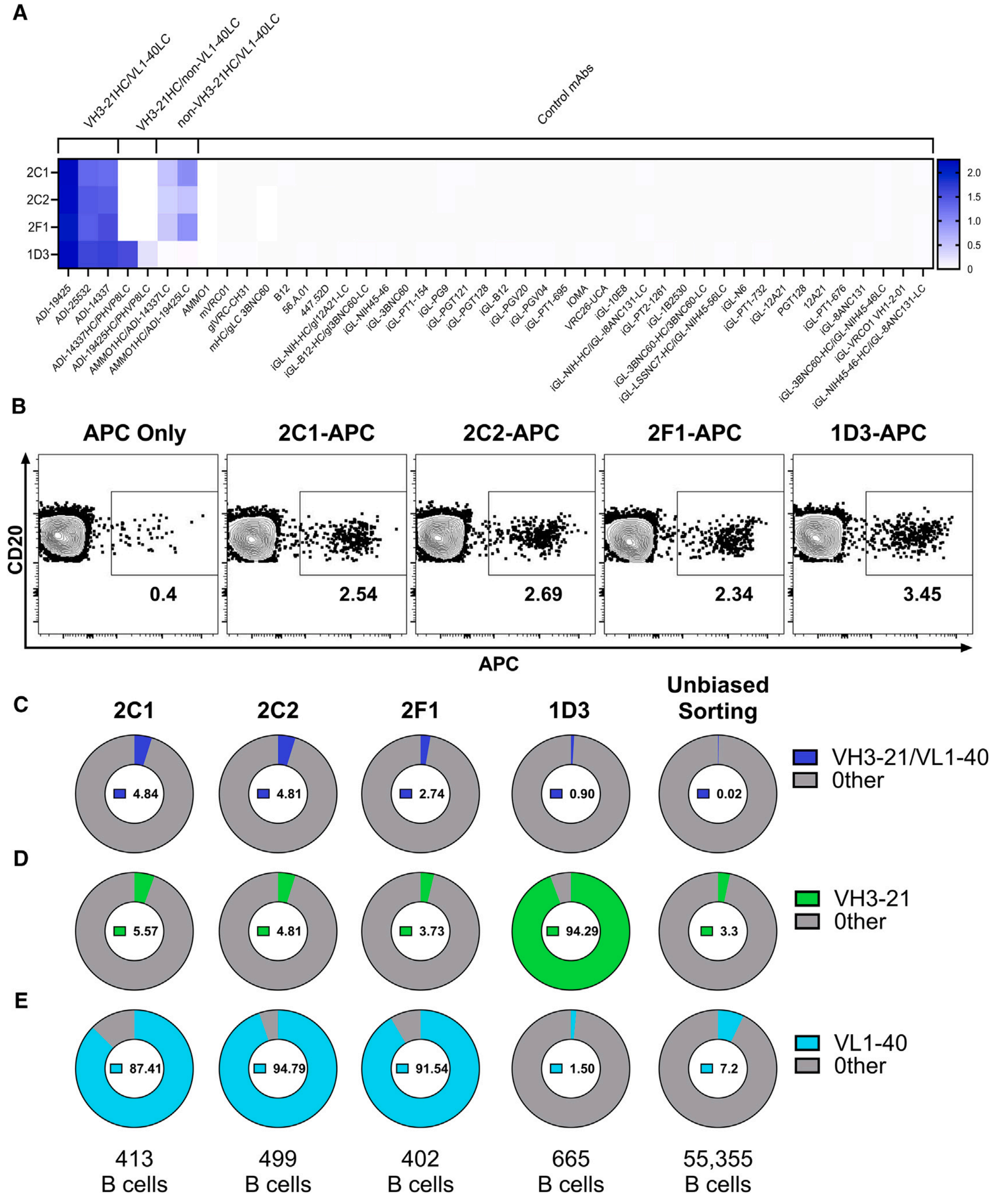

Figure 1. Characterization of anti-idiotypic antibodies specific for antibodies encoded by VH3-21 and VL1-40 genes

(A) IgGs purified from hybridoma supernatants 2C1, 2C2, 2F1, and 1D3 were evaluated for their ability to bind to a panel of recombinant mAbs by ELISA. The scale indicates the A_{450} signal as measured by ELISA. Data shown are average of duplicate wells from one biological replicate.

(B) ai-mAbs were fluorescently labeled with allophycocyanin (APC) conjugated to a unique oligonucleotide barcode and used to stain naive B cells from human PBMCs. The frequency of APC^+ B cells is shown. The gating strategy is shown in Figure S2.

(C–E) APC^+ naive B cells were sorted, and the paired BCR transcripts were obtained through next-generation sequencing. Shown are (C) the percentage of VH3-21/VL1-40 pairs among all BCR sequences sorted by each ai-mAb bait (blue), (D) the percentage of BCRs expressing an HC derived from the VH3-21 gene sorted by each ai-mAb (green), and (E) the percentage of BCRs expressing an LC derived from the VL1-40 gene sorted by each ai-mAb bait (teal). The number of B cells analyzed is shown at the bottom of (C)–(E). The frequency of naive B cells expressing these genetic features identified by high-throughput unbiased sorting is included for comparison in[37] (C)–(E). Data are representative of one biological replicate for each ai-mAb in (B)–(E).

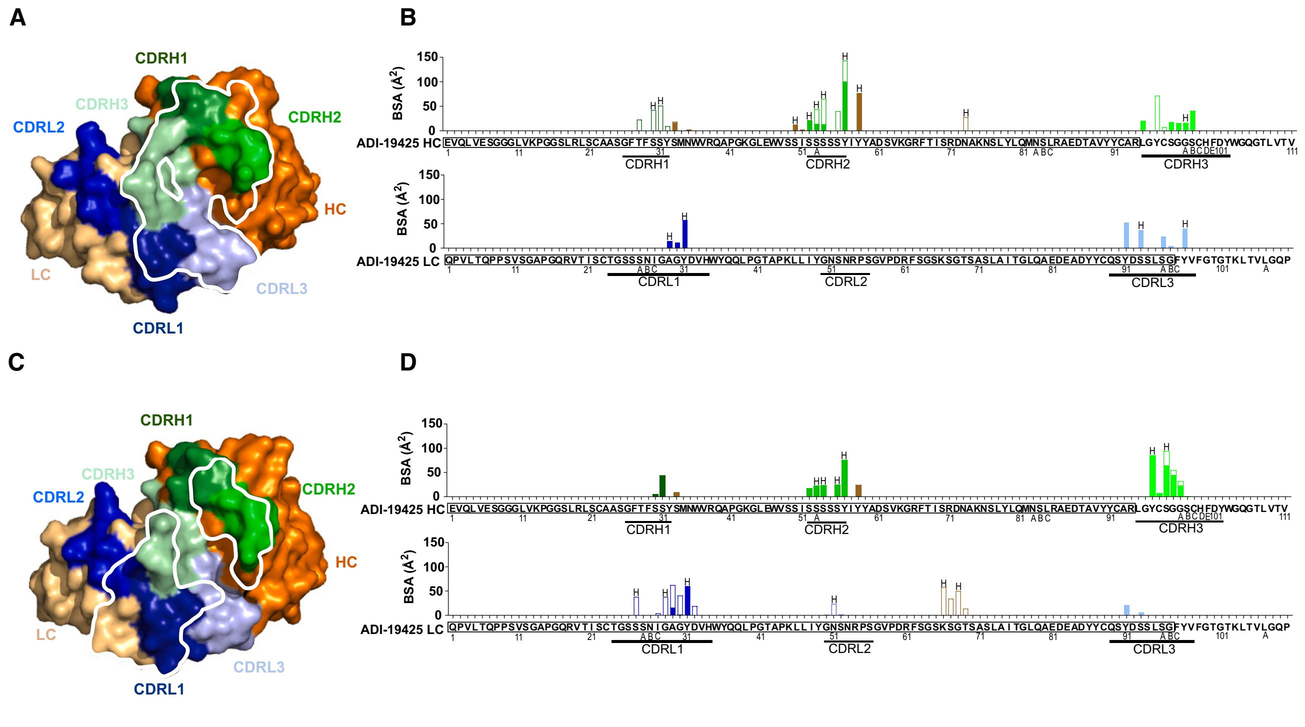

Figure 2. Crystal structures of ai-mAbs in complex with ADI-19425

(A and C) Surface representation of ADI-19425 Fab bound to (A) 1D3 Fab and (C) 2C1 Fab. Views are looking down on the ADI-19425 paratope. Residues within 5 Å of (A) 1D3 or (C) 2C1 are outlined in white.

(B and D) Buried surface area (BSA) plots shown as stacked bar graphs of ADI-19425 HC (top) and ADI-19425 LC (bottom) bound by (B) 1D3 Fab or (D) 2C1 Fab. Interactions with the HC of either 1D3 or 2C1 are shown as solid-colored bars, while interactions with the LC of either 1D3 or 2C1 are shown as empty bars. Residues participating in hydrogen-bond interactions are labeled with an "H." ADI-19425 HC and LC sequences are shown underneath the graph. VH3-21 and VL1-40 sequences are boxed.

See also Figure S3 and Table S1.

Approximately 95% of B cells sorted with 1D3 as bait expressed VH3-21 HCs (Figure 1D), and ∼85%–95% of B cells sorted with 2C1, 2C2, and 2F1 expressed VL1-40-encoded LCs (Figure 1E). Sequencing of the hybridomas revealed that 2C1, 2C2, and 2F1 were all clonal variants. Collectively these results indicate that the epitope of 1D3 is primarily encoded by the VH3-21 gene, and that of 2C1, 2C2, and 2F1 is primarily encoded by the VL1-40 gene.

Crystal structures of ADI-19425 complexed with 1D3 and 2C1

To understand the molecular basis behind the observed interactions between the ai-mAbs and BCRs derived from VH3-21 and VL1-40, we sought to obtain their co-crystal structures with ADI-19425, a VH3-21/VL1-40-derived RSV neutralizing mAb for which the co-crystal structure with preF has been solved previously.[9] 1D3 and 2C1 were produced as recombinant immunoglobulin Gs (IgGs) and their binding to the ADI-19425 antigen binding fragment (Fab) was confirmed by biolayer interferometry (BLI) (K_D = 0.4 nM for 1D3 and 1.5 nM for 2C1; Figures S3A and S3B). The ai-mAbs were then digested into Fabs and complexed with ADI-19425 Fab to enable structural analysis. We solved the crystal structures of the ADI-19425 Fab in complex with the 1D3 Fab at 2.67 Å and the ADI-19425 Fab in complex with the 2C1 Fab at 2.41 Å (Figures 2A, 2C, S3C, and S3D; Table S1). ADI-19425 binds 1D3 with a total buried surface area (BSA) of ∼1,019 Å^2, primarily through its HC with ∼579 Å^2 (57% of total BSA) from the VH3-21-encoded region and ∼190 Å^2 (19% of total BSA) from the CDRH3. The remainder, ∼208 Å^2 (20% of total BSA), is contributed by the ADI-19425 VL1-40-encoded germline gene and ∼42 Å^2 (4% of total BSA) from the lambda J gene (Figures 2A and 2B). ADI-19425 binds 2C1 with a total BSA of ∼994 Å^2, ∼471 Å^2 (47% of the total BSA) of which is contributed from its VL1-40-encoded germline gene, while ∼248 Å^2 (25% of the total BSA) comes from its VH3-21. The CDRH3 contributes ∼275 Å^2 (28%) of the total BSA (Figures 2C and 2D).

Superposition of the crystal structures showed that both ai-Fabs bound the ADI-19425 head on with some overlap (Figures S3C–S3E). Five ADI-19425 HC residues (Tyr97–Gly 100A, Kabat numbering) and one LC residue (Tyr91) interact with both ai-Fabs. These structural analyses support the observation that 1D3 and 2C1 preferentially engage BCRs derived from the VH3-21 and VL1-40 genes, respectively.

Generation of a VH3-21/VL1-40-directed bispecific ai-mAb

Based on the ai-mAb binding and B cell sorting results (Figure 1), none of the ai-mAbs show the desired specificity for

CellPress
OPEN ACCESS

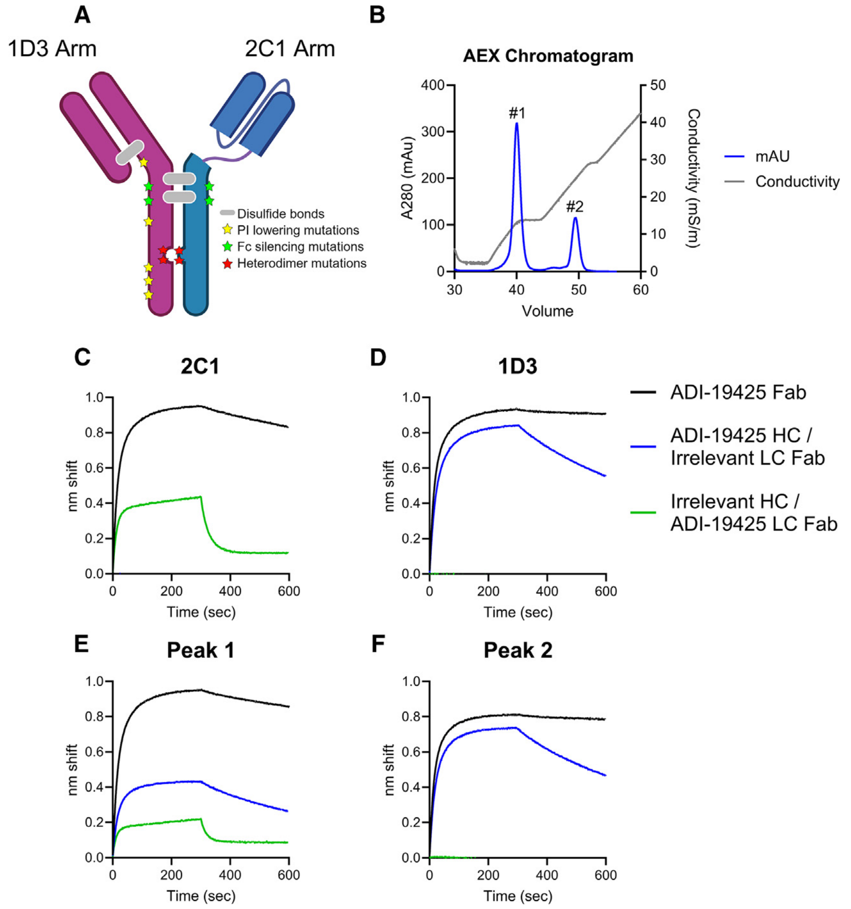

Figure 3. Engineering a VH3-21/VL1-40 BCR-targeting bispecific ai-mAb

(A) Schematic of the bispecific platform used,[39] created with BioRender.

(B) Anion exchange (AEX) chromatogram of bispecific ai-mAb production. Representative data from one biological replicate are shown.

(C–F) Binding of ADI-19425 Fab and the indicated chimeric Fabs to recombinant 2C1-IgG (C), 1D3-IgG (D), IgG from peak 1 (E), or IgG from peak 2 (F) was measured by BLI. Representative data are from one biological replicate.

paired VH3-21/VL1-40 BCRs. To enhance the specificity of these ai-mAbs to target VH3-21/VL1-40 BCR pairs, we sought to engineer a bispecific ai-mAb that combines the binding properties of 2C1 and 1D3. We cloned the VH and VL sequences of 1D3 and 2C1 into an engineered heterodimeric Fc platform that permits the efficient purification of bispecific mAbs.[39] The bispecific format contains mutations in the Fc region that lower the isoelectric point (Figure 3A, yellow stars), promote heterologous Fc pairing (Figure 3A, red stars), and disrupt binding to Fc receptors (Figure 3A, green stars). The expression plasmids were co-transfected into HEK293E cells and purified by protein A affinity followed by anion exchange chromatography, the latter of which yielded two peaks eluting at 13.1 mS (∼40-mL elution volume) and 24.8 mS (∼49.5-mL elution volume) (Figure 3B). We evaluated the binding of each peak as well as the parental recombinant 2C1 and 1D3 ai-mAbs to the VH3-21/VL1-40 encoded anti-RSV Fab ADI-19425 as well as chimeric Fabs by BLI (Figures 3C–3F). 2C1 bound ADI-19425 and the ADI-19425LC chimera (Figure 3C), while 1D3 bound ADI-19425 and the ADI-19425HC chimera (Figure 3D). The ai-mAb that eluted in the first peak of the anion exchange column bound ADI-19425 and both chimeras, while the ai-mAb from the second peak only bound ADI-19425 and the ADI-19425HC chimera. We therefore conclude that the desired bispecific ai-mAb eluted in the first peak. We carried out subsequent experiments with the desired 2C1-1D3 bispecific ai-mAb from the ∼13 mS/m peak.

The fluorescently labeled bispecific ai-mAb efficiently engages *bona fide* VH3-21/VL1-40 BCRs

We used the fluorescently labeled bispecific ai-mAb as bait in single-B cell sorting experiments (Figure 4A). Naive B cells that were bound to the labeled bispecific ai-mAb (Figure 4A right) were single cell sorted, and their VH and VL transcripts were recovered by RT-PCR[40] and sequenced to determine their gene usage using ImMunoGeneTics immunoglobulin search tool (IMGT/V-QUEST).[41,42] Sequencing results from three independent sorting experiments, each using peripheral blood mononuclear cells (PBMC) from a unique donor were combined for analysis. In total, 270 B cells were sorted, yielding 211 productive HCs, 216 productive LCs, and 159 productive paired BCR sequences (Figure 4B). 86% and 44% of the B cells sorted with the bispecific expressed VH3-21 HCs and VL1-40 LCs, respectively, representing 26.2- and 7.6-fold enrichment above the estimated gene frequencies determined by unbiased high-throughput paired sequencing of paired BCRs from naive B cells from three unique donors[37] (Figure 4B).

Importantly, the bispecific ai-mAb was able to efficiently engage B cells expressing the desired VH3-21/VL1-40 BCR gene pairing. Among ai-mAb-sorted B cells for which paired sequences of the HC and LC were recovered, 40% expressed VH3-21/VL1-40 pairs, representing a 207-fold enhancement over the estimated gene frequencies of 0.19% (Figure 4B).[37] This enrichment demonstrates that the bispecific antibody has a high-degree of specificity for natively paired VH3-21/VL1-40 BCRs.

mAbs from bispecific ai-mAb-sorted VH3-21/VL1-40 B cells cross-react with preF RSV F

In all, 64 discrete VH3-21/VL1-40 BCR$^+$ B cells were recovered over three independent sorting experiments. To determine the functional properties of the BCRs, they were produced as recombinant mAbs. 60 of the 64 paired VH3-21/VL1-40 BCR

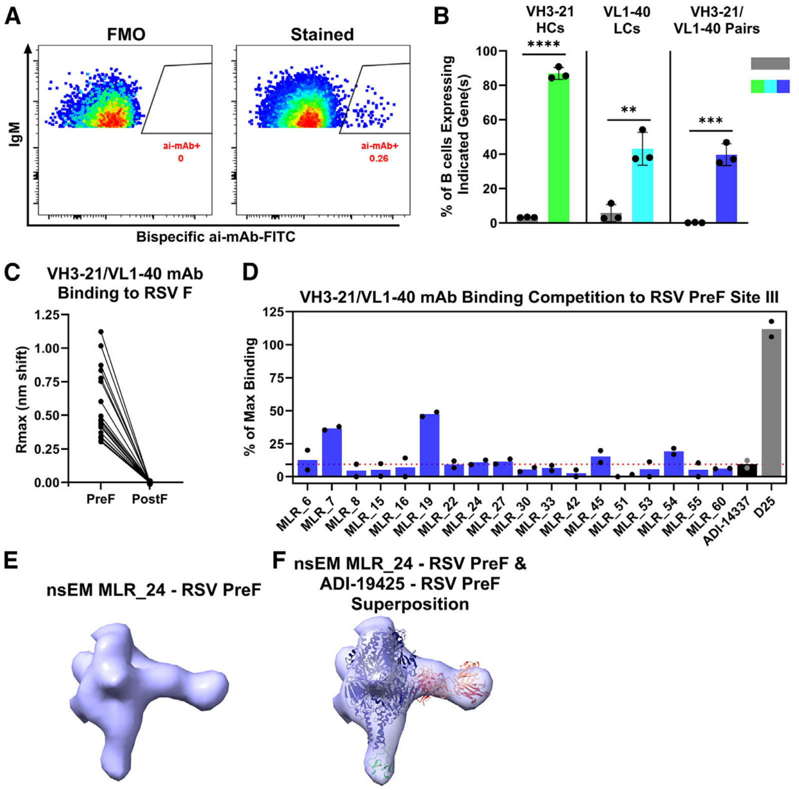

Figure 4. Bispecific ai-mAb-specific B cell sorting and characterization

(A) A fluorescently-labeled 1D3/2C1 bispecific ai-mAb was used to stain and sort naive human B cells; a representative sorting plot is shown, with FMO representing a fluorescence minus one (no ai-mAb) staining control. See Figure S4 for the gating strategy.

(B) From the sorted cells, the percentages of productive VH3-21 HCs (green) and VL1-40 LCs (teal) are shown. From successfully recovered pairs, the percentages of VH3-21/VL1-40 (blue) are shown. Each data point represents the frequency of gene usage from 3 independent sorting experiments. Bars represent the mean ± standard deviation. Naive BCRs obtained from unbiased high-throughput sequencing of 3 unique donors[37] are shown for comparison (gray). Significant differences were determined using Student's two-tailed t tests. $^{**}p = 0.001$–0.01, $^{***}p = 0.0001$–0.001, $^{****}p < 0.0001$.

(C) VH3-21/VL1-40 BCRs from (B) were expressed as recombinant mAbs and tested for binding to RSV preF and postF by BLI. Data shown are average from 2 biological replicates.

(D) mAbs from (C) were tested for their ability to compete for binding with ADI-14337[9] to preF by BLI. Each data point represents a biological replicate, and the bars represent mean. The red dotted line represents the average ADI-14337 competition against itself. D25 is an RSV preF site Ø mAb[12] and is included as a negative control.

(E) Side view of a representative nsEM 3D class of the DS-Cav1-MLR_24 Fab complex.

(F) Superposition of the crystal structure of ADI-19425 Fab bound to preF[9] (PDB: 6APD) into the representative 3D volume. The crystal structure is colored as follows: RSV preF (midnight blue), ADI-19425 HC (dark red), and ADI-19425 LC (salmon). Density was also seen for the T4 fibritin trimerization domain, which is modeled by the foldon region of the GlyProPro(10)-foldon crystal structure (PDB: 1NAY) in green.

See also Figures S4–S6 and Table S2.

sequences were successfully expressed as recombinant mAbs and screened for binding to RSV preF (DS-Cav1)[15] and postF[43] by BLI. 18 of 60 or 30% of the sorted germline VH3-21/VL1-40 mAbs bound preF but not postF (Figure 4C). In agreement with Goodwin et al., we were unable to identify a unique CDHR3 or CDRL3 signature among preF-binding VH3-21/VL1-40 mAbs, except for a slight preference toward usage of glycine, serine, and tyrosine residues at CDRH3 positions 96–100c[9] (Figure S5B). There was an enrichment for IGHJ4 gene usage in preF-binding mAbs (83.33%) vs. non-binding mAbs (42.86%; Figures S5E and S5F) and a slight enrichment for IGLJ2 gene usage in preF-binding mAbs (61.11%) vs. non-binding mAbs (52.38%; Figures S5K and S5L).

The majority of VH3-21/VL1-40 derived RSV-binding mAbs target antigenic site III on RSV preF.[5,9,11,44] To evaluate whether the VH3-21/VL1-40 mAbs sorted with the bispecific ai-mAb also target this site, competitive binding assays to RSV preF were performed by BLI. 16 of the 18 VH3-21/VL1-40 mAbs that bound RSV preF showed near-complete inhibition by ADI-14337 binding to preF, consistent with site III binding (Figure 4D). Two mAbs showed relatively lower inhibition by ADI-14337 for binding to preF, potentially targeting an epitope partially overlapped by that of ADI-14337 (MLR_7 and MLR_19; Figure 4D). Negative-stain electron microscopy analysis of one representative Fab in complex with DS-Cav1, MLR_24, which showed the highest binding signal to preF (Figure 4C), revealed that it binds preF comparably to ADI-19425 Fab[9] (Figures 4E and 4F). However, we were only able to resolve 1 Fab bound per trimer (Figures 4E, 4F, and S6), which differs from the AM22/ADI-19425/PR-DM crystal structure, which showed binding of 3 site III Fabs per trimer. Collectively, these data demonstrate that the bispecific ai-mAb is capable of selectively engaging B cells expressing VH3-21/VL1-40 BCRs that recognize a site III epitope present on preF.

Unmutated VH3-21/VL1-40 mAbs sorted with the bispecific ai-mAb neutralize RSV subtypes A and B

The sorted VH3-21/VL1-40 mAbs were evaluated for neutralizing activity against RSV subtypes A (A2) and B (B1) in plaque reduction assays.[44] All preF-binding mAbs were initially tested at 500 μg/mL (Figures S7A and S7B). Three (MLR_15, MLR_24, and MLR_55) showed neutralizing activity and were titrated to determine their half-maximal inhibitory concentration (IC_{50}; Figures 5A and 5B). MLR_15 exhibited weak (130.6 μg/mL) activity against RSV-A, while MLR_24 was more potent (24.2 μg/mL) (Figures 5A and 5B). These mAbs showed a similar hierarchy but were more potent against RSV-B (MLR_15, 21.75 μg/mL; MLR_24, 0.22 μg/mL). MLR_55 weakly neutralized RSV-B (98.2 μg/mL) (Figures 5A and 5B). MLR_24 was ~100-fold

CellPress
OPEN ACCESS

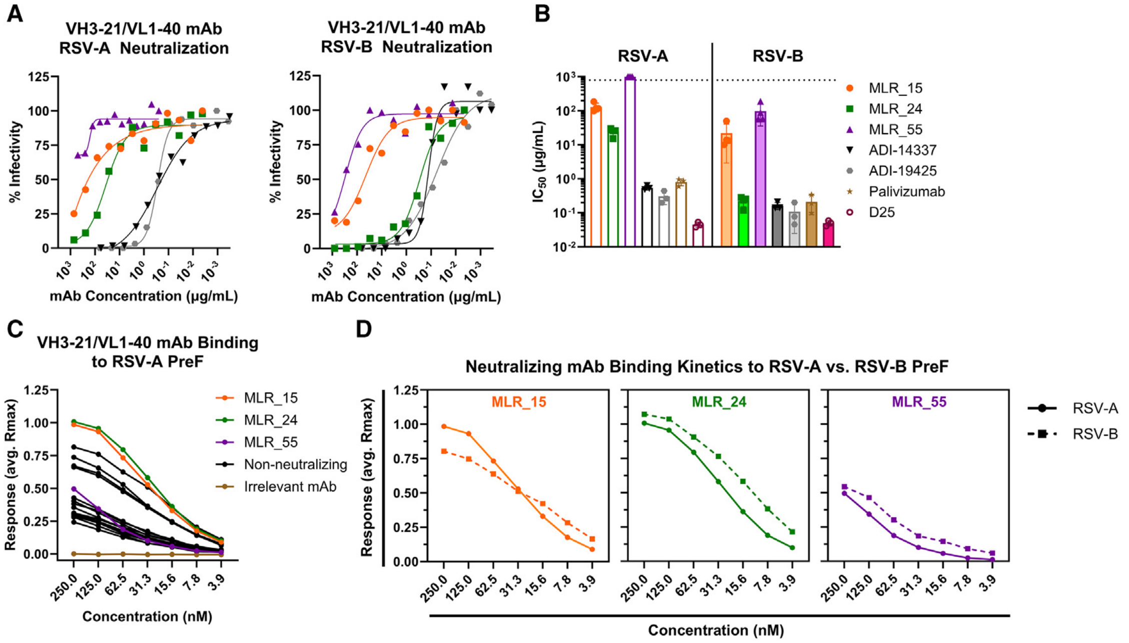

E

mAb	RSV-A PreF K_D (M)	RSV-B PreF K_D (M)
MLR_15	2.49E-09	1.41E-09
MLR_24	1.29E-09	4.58E-10
MLR_55	3.36E-09	2.97E-09

Figure 5. VH3-21/VL1-40 mAb RSV neutralization and affinity

(A) Neutralization curves of selected VH3-21/VL1-40 mAbs against RSV-A (left) and RSV-B (right). Points shown are the mean of 2 technical replicates from a single biological replicate.

(B) Mean IC_{50} values of selected mAbs against RSV-A and RSV-B. Each dot represents a biological replicate (n = 3–4), and bars represent mean ± standard deviation. The dotted line represents maximum tested concentration.

(C) Steady-state analysis of VH3-21/VL1-40 mAb binding response to RSV-A preF measured by BLI. Each data point represents the mean response from two biological replicates.

(D) Steady-state kinetic analysis of neutralizing VH3-21/VL1-40 mAbs binding to RSV-A (solid lines) and RSV-B (dashed lines) preF measured by BLI. Each data point represents the mean response from two biological replicates.

(E) Apparent affinity of neutralizing VH3-21/VL1-40 mAbs to RSV-A/RSV-B preF.

See also Figure S7.

more potent against RSV-B than RSV-A, and it showed potency comparable to other unmutated VH3-21/VL1-40 mAbs, ADI-14337 and ADI-19425, and to palivizumab against RSV-B[9,17] (Figures 5A and 5B). In our assay, the state-of-the-art prophylactic D25 (niresvimab) showed the most potent neutralization, ∼500-fold more potent than MLR_24 against RSV-A and ∼4.5-fold more potent than MLR_24 against RSV-B (Figure 5B). Among the population of naive VH3-21/VL1-40 antibodies sorted with the bispecific ai-mAb, 3 of 60 (5%) had neutralizing activity against RSV, demonstrating that an anti-idiotype immunogen can target RSV-neutralizing B cells and further illustrating the feasibility of using this vaccine platform to target defined, neutralizing B cell lineages.

The binding kinetics of VH3-21/VL1-40 mAbs dictate neutralization potential

We next assessed whether differences in binding strength of the VH3-21/VL1-40 mAbs could discriminate between those that neutralize and those that do not. To this end, their relative steady-state binding to RSV-A preF was measured by BLI. mAbs MLR_15 and MLR_24 demonstrated the highest binding responses over a range of mAb dilutions (Figure 5C, orange and green), while the MLR_55 response was lower (Figure 5C, purple). An examination of the dissociation rates (K_D) from this dataset revealed that MLR_55 had the slowest dissociation rate of all expressed mAbs (Figure S7C). According to this analysis, the neutralizing mAbs bifurcate from the non-neutralizers based on overall binding avidity and/or slow dissociation rates, indicating that binding kinetics are a key determinant of neutralization potency among VH3-21/VL1-40 mAbs. Interestingly, MLR_15 and MLR_55 utilized the same LC sequence, suggesting that HC differences accounted for the observed variation in neutralization potency and apparent affinity to RSV preF (Figures S5M and S5N).

To further refine the avidity analysis of the neutralizing mAbs (MLR_15, MLR_24, and MLR_55), the steady-state binding to

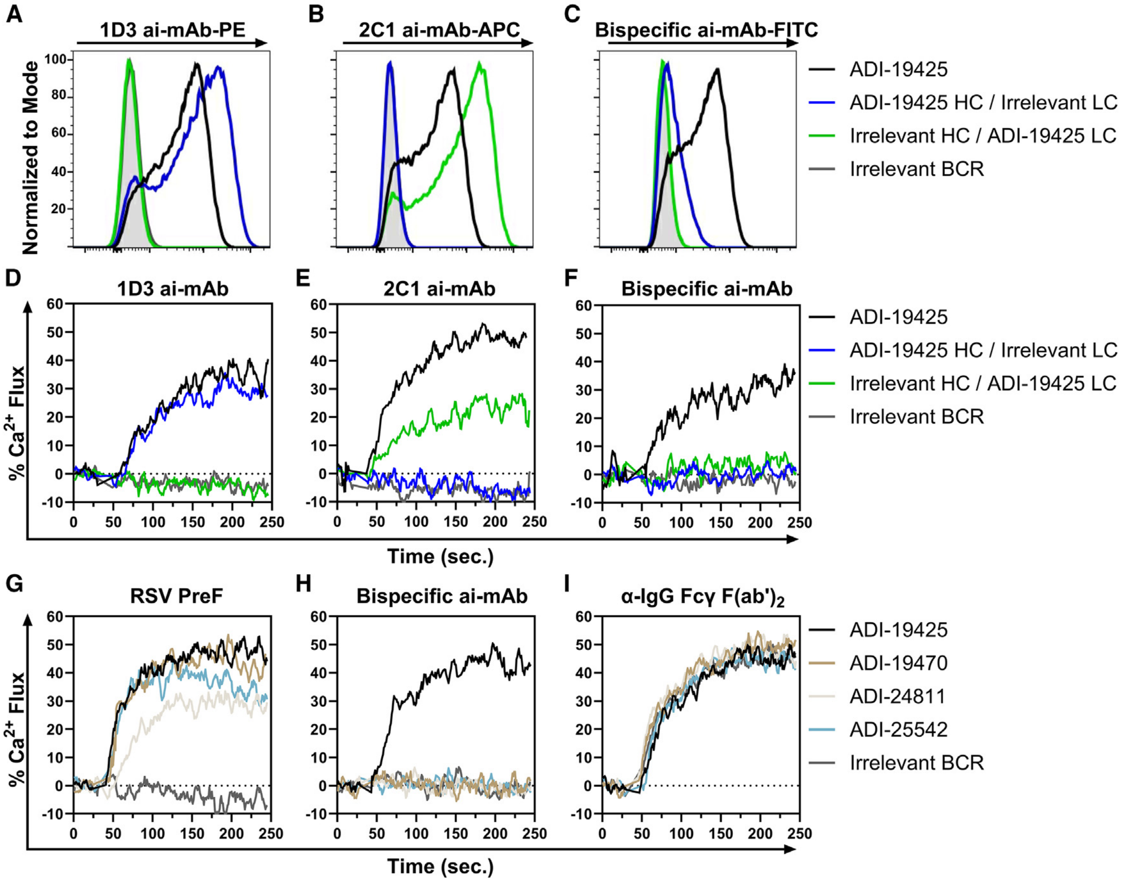

Figure 6. Activation of on- and off-target BCR-expressing cell lines by calcium flux assays

(A–C) Staining of DG75 B cell lines transduced to express the indicated BCRs, shown as histograms, with signals normalized to mode. Shown is a representative plot of a single biological replicate.

(A) Staining with the 1D3 ai-mAb.

(B) Staining with the 2C1 ai-mAb.

(C) Staining with the bispecific ai-mAb.

(D–F) Calcium flux in response to the addition of the 1D3 ai-mAb D, 2C1 ai-mAb E, or the 1D3/2C1 bispecific ai-mAb F as immunogen. Signals shown are representative of 2 biological replicates.

(G–I) Calcium flux in non-neutralizing RSV preF-specific BCR-expressing cell lines as indicated. Cell lines were stimulated with RSV-A preF (G), the bispecific ai-mAb (H), and, as a positive control, an α-IgG Fcγ F(ab′)$_2$ (I). Signals shown are representative of 2 biological replicates.

RSV-B[45] preF and RSV-A was compared (Figure 5D, dotted vs. solid line). This analysis revealed that the neutralizing VH3-21/VL1-40 mAbs MLR_24 and MLR_55 bound more strongly to RSV-B preF than to RSV-A preF at every concentration tested (Figure 5D). At the higher end of the dilution series, MLR_15 showed higher binding to RSV-A preF, but this reversed at the lower end of the dilution series, while the half-maximal binding was nearly equivalent for both preF variants (Figure 5D, left), consistent with the smaller discrepancy between the neutralization potency against RSV-A and RSV-B (Figures 5A and 5B). We also determined the apparent affinity (K_D) for each neutralizing mAb against RSV-A/RSV-B preF (Figure 5E). All mAbs demonstrated higher apparent affinity for RSV-B vs. RSV-A, with MLR_24 showing the greatest difference in apparent affinity (2.8-fold). The stronger binding to RSV-B preF by these mAbs supports their higher potency to neutralize this subtype.

The bispecific ai-mAb selectively activates B cells with desired BCR gene pairing

Previous studies have demonstrated that naive B cells sorted with an antigen are representative of those that will respond to a matched immunization in humans.[46–48] Because the bispecific ai-mAb can engage natively paired BCRs with the VH3-21/VL1-40 gene pairing (Figure 4), we hypothesized that this could be used as an immunogen to activate these target B cells. However, the binding and sorting data (Figures 3 and 4) also indicate that the bispecific ai-mAb can bind off-target BCRs that have either the target HC or LC but without the desired pairing. Since B cell activation requires cross-linking of adjacent cell-surface BCRs, we hypothesized that the bispecific ai-mAb would preferentially activate paired target B cells. To test this directly, we generated human B cell lines stably expressing the ADI-19425 BCR, the ADI-19425 HC paired with an irrelevant LC, or an irrelevant HC paired

CellPress
OPEN ACCESS

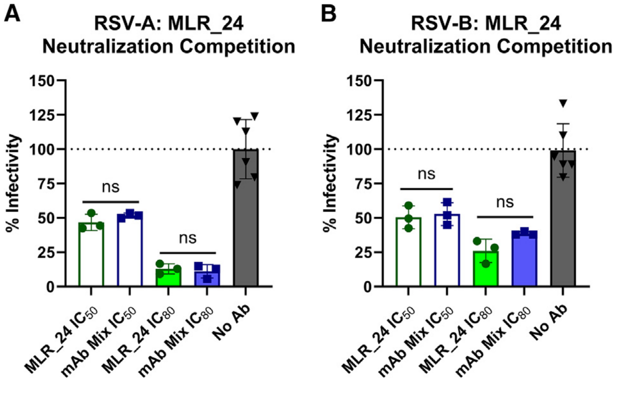

Figure 7. RSV neutralization competition assays

MLR_24 was formulated at its IC_{50} (green, open) or IC_{80} (green, shaded) concentration alone or in combination with 5 non-neutralizing, pre-F-binding mAbs at matched concentrations "mAb Mix" (blue open, blue shaded) (Figures S7A and S7B) and tested for inhibition of RSV-A (A) and RSV-B (B) in a plaque reduction assay. Each dot represents a single biological replicate carried out in triplicate, and bars represent mean ± standard deviation. Significance was determined by Mann-Whitney tests comparing MLR_24 to mAb Mix at each concentration.

with the ADI-19425 LC. The B cell lines were stained with fluorescently labeled 1D3 and 2C1 ai-mAbs as well as the 1D3/2C1 bispecific ai-mAb (Figures 6A–6C). 1D3 bound to B cells expressing the ADI-19425 BCR (black) and the B cells expressing the ADI-19425HC chimera BCR (blue), while 2C1 bound to the ADI-19425 BCR and the ADI-19425LC BCR chimera (green) (Figures 6A and 6B). The bispecific ai-mAb showed bright staining of the ADI-19425 cells (Figure 6C, black) but only modest staining of the ADI-19425HC chimera cell line (Figure 6C, blue). The bispecific ai-mAb did not bind the ADI-19425LC chimeric cells, likely because the chimeric BCR lacks contact residues encoded by the VH3-21 HC (Figures 2C and 2D), resulting in an increased dissociation rate and weaker binding (Figures 3C and 3E).

We next measured the ability of the mono- and bispecific ai-mAbs to activate the BCR-expressing cell lines from Figures 6A–6C using a flow cytometry-based calcium flux assay (Figures 6D–6F).[49,50] 1D3 and 2C1 activated cells expressing the ADI-19425 BCR as well as the VH3-21 and VL1-40 chimeric BCRs, respectively (Figures 6D and 6E). In contrast, the bispecific ai-mAb only activated the ADI-19425 cell line (Figure 6F). None of the ai-mAbs activated DG-75 cells expressing a non-VH3-21/non-VL1-40 control BCR (Figures 6D–6F, gray).[35]

To further evaluate the specificity of the bispecific ai-mAb to activate B cells harboring the VH3-21/VL1-40 gene pair, we engineered three additional B cell lines expressing unmutated, non-neutralizing, non-site III, preF-specific BCRs, none of which are derived from VH3-21 or VL1-40 (ADI-19470, ADI-24811, and ADI-25542).[9] All cell lines, including ADI-19425, were activated by the addition of RSV-A preF or a positive control; however, only ADI-19425 was activated by the addition of the bispecific ai-mAb (Figures 6G–6I). Collectively, these data indicate that the bispecific ai-mAb preferentially activates B cells with paired VH3-21/VL1-40 BCRs but not off-target preF-specific non-neutralizing B cells that are activated by currently approved protein subunit vaccines based on preF.

Unmutated VH3-21/VL1-40 mAb maintains neutralization potency in the presence of preF-binding, non-neutralizing mAbs

The calcium flux experiments demonstrate that the ai-mAb can engage and activate target-neutralizing VH3-21/VL1-40 B cells, suggesting that these B cells could respond to immunization with the ai-mAb and secrete neutralizing antibodies. Assuming this is true, the B cell sorting experiments demonstrate that the ai-mAb also readily recognizes non-neutralizing VH3-21/VL1-40 B cells, which would similarly respond to immunization. We therefore sought to determine whether the presence of an excess of non-neutralizing VH3-21/VL1-40 mAbs affects the potency of neutralizing VH3-21/VL1-40 mAbs. To test this experimentally, plaque reduction assays were set up against RSV-A and RSV-B where MLR_24 was present at a concentration equivalent to its IC_{50} or IC_{80} along with a combination of 5 non-neutralizing, preF-binding mAbs at equal concentrations. The potency of MLR_24 against RSV-A and RSV-B was unaffected by a 5-fold excess of non-neutralizing preF-binding mAbs (Figures 7A and 7B), indicating that the neutralizing activity of VH3-21/VL1-40 mAbs is maintained in the presence of an excess of non-neutralizing, site III-directed VH3-21/VL1-40 mAbs.

DISCUSSION

Here we describe the isolation and characterization of a bispecific molecule derived from anti-idiotypic antibodies designed to target a conserved class of B cells capable of producing antibodies that potently neutralize RSV without undergoing affinity maturation. The bispecific ai-mAb readily engages B cells expressing target BCRs among diverse pools of naive B cells *ex vivo* and selectively activates target B cells *in vitro*, providing proof of concept that the bispecific ai-mAb could be used to elicit protective antibodies if used as an immunogen.

Our binding and sorting experiments determined that the bispecific ai-mAb can also bind off-target cells, such as those with either a VH3-21 HC or a VL1-40 LC. However, the bispecific design is intended to promote specific BCR cross-linking and activation of target B cells through engagement of a VH3-21 HC with the 1D3 arm and a VL1-40 LC on an adjacent BCR with the 2C1 arm while enhancing the strength of the binding interaction through avidity. In support of this, the calcium flux assays demonstrate specific activation of B cells expressing on-target BCRs. Moreover, the bispecific ai-mAb failed to activate B cells expressing non-neutralizing, F-reactive BCRs that are otherwise activated by a clinically tested preF vaccine immunogen, highlighting the ability of the bispecific ai-mAb to selectively activate B cells specific for a known neutralizing epitope on F.

Through naive B cell sorting experiments, we demonstrated that the bispecific ai-mAb readily engages B cells expressing the desired germline-encoded VH3-21/VL1-40 BCRs, and they were enriched >200-fold above the estimated gene pair frequencies present in the naive B cell repertoire.[37] When produced as recombinant mAbs, 30% showed measurable binding to preF, while none bound postF. Nearly all of these mAbs mapped to site III, consistent with the binding profile of the VH3-21/VL1-40 antibody class. Among the F-binding VH3-21/VL1-40 mAbs,

three (5%) neutralized RSV subtypes A and B, demonstrating that a non-F immunogen can readily target RSV-neutralizing BCRs in relevant samples. A recent study similarly used ai-mAbs to isolate primary B cells capable of producing neutralizing antibodies against influenza, providing additional proof of concept that ai-mAbs can be used to target neutralization-competent B cell subsets with defined genetic features.[51]

Although only 5% of B cells sorted by the bispecific ai-mAb were neutralizing, based on the frequency of the VH3-21/VL1-40 gene pair in the naive B cell repertoire, we estimate that the bispecific ai-mAb is capable of engaging 1 neutralizing B cell in ~5,500 to 22,000 B cells.[37] Given that previous studies have shown that B cells sorted with germline-targeting immunogens are representative of those that respond to immunization in humans,[46–48,52] these data suggest that vaccination with the bispecific ai-mAb will elicit a VH3-21/VL1-40-dominated response that will include RSV-binding and neutralizing antibodies.

Our results demonstrate that not all germline VH3-21/VL1-40 mAbs bind to preF, and of these, only a fraction are capable of neutralizing RSV. This is in agreement with a study by Goodwin et al.[9] that found that 40% of naive, preF-reactive, VH3-21/VL1-40 (or the related VH3-11/VL1-40) B cells from cord blood or naive B cells from adults were able to neutralize RSV. The lower percentage observed here may be due to differences in the method used to select cells for analysis: using a preF probe for sorting vs. targeting the gene pairing itself. Although the bispecific ai-mAb engaged B cells expressing VH3-21/VL1-40 BCRs that were both neutralizing and non-neutralizing, mAbs derived from the neutralizing B cells bound more tightly to preF, suggesting that they would have a competitive advantage in binding F over the non-neutralizing mAbs. This notion is supported by the observation that MLR_24 maintained neutralization potency even in the presence of an excess of non-neutralizing, VH3-21/VL1-40 preF-binding mAbs.

Previous studies have found that VH3-21/VL1-40-derived mAbs neutralize both subtypes of RSV with comparable potency, likely due to the relative conservation of site III on preF.[5,9,11,53,54] However, the VH3-21/VL1-40 mAbs isolated here, and the ADI-19425 control mAb, exhibited overall higher potency against RSV-B than RSV-A (~6-fold for MLR_15, ~100-fold for MLR_24, and ~3-fold for ADI-19425). This trend (average IC_{50} of 3.73 μg/mL against RSV-A vs. 2.98 μg/mL for RSV-B) was observed among the 25 unmutated VH3-21/VL1-40 mAbs isolated by Goodwin et al.[9] that showed neutralization potency. The larger discrepancy seen in this study can in part be attributed to the difference in neutralization assays (microneutralization vs. plaque reduction) across studies and may not be the case among all F-reactive VH3-21/VL1-40 BCR^+ B cells that could be targeted by the bispecific ai-mAb. Nevertheless, the ai-mAb efficiently targeted the naive B cells of the VH3-21/VL1-40 BCR class, including those that are neutralizing.

In our plaque-reduction assays, MLR_24, the most potent mAb isolated here, was ~4.5- and ~500-fold less potent than the current state-of-the-art prophylactic mAb D25 (nirsevimab) against RSV-B and RSV-A, respectively. Passive transfer studies in susceptible animals with the mAbs isolated here could define whether and at what levels these mAbs would need to be present for protection,[11,12,44] providing a benchmark for future vaccine studies. Despite only isolating mAbs with moderate potency against RSV-A, it is plausible that the ai-mAb could engage BCRs encoding more potent mAbs. This notion is supported by the observation that the ai-mAb binds with high affinity to ADI-19425 and activates B cells expressing this BCR. The fact that we did not identify more potent mAbs here could be attributed to inefficiencies in B cell sorting and VH and VL amplicon recovery as well as the inherent undersampling of the B cell repertoire by analyzing peripheral blood samples. Nevertheless, our data demonstrate that the ai-mAb is capable of engaging BCRs that correspond to RSV-neutralizing mAbs.

A theoretical drawback of using ai-mAbs to target B cells is that the activated B cells may enter germinal centers, undergo somatic mutation, and gain affinity for the ai-mAb at the cost of losing affinity for preF. Although some B cells could plausibly suffer this fate, it is also likely that a substantial portion of activated B cells, including those that encode neutralizing antibodies, will rapidly become antibody-secreting plasmablasts.[55–58] In support of this, we have shown previously that a single immunization with a high-affinity ai-mAb rapidly induced a robust on-target plasmablast response and corresponding high-titer serum antibody responses that persisted for 6 months in a murine adoptive transfer model.[33] Further, elevated levels of early memory B cells were also identified in this ai-mAb immunization group.[33] Another theoretical consequence of using ai-mAbs as immunogens is that they may bind and mediate killing through Fc-mediated functions. To ameliorate this possibility, we included mutations in our bispecific design to ablate Fc binding.[39]

Based on the naive B cell sorting analyses by Goodwin et al.[9] and this study, both the ai-mAb and preF can engage and activate unmutated VH3-21/VL1-40 RSV-neutralizing B cells. The bispecific ai-mAb differs from F in that it specifically engages site-III directed B cells and avoids activating non-neutralizing B cells directed at other epitopes on F. This approach has potential advantages over F-based vaccine strategies to protect infants from RSV infection. An ai-mAb with high affinity and specificity for VH3-21/VL1-40-class antibodies may be particularly well suited to elicit neutralizing antibodies and establish immunological memory in the first 6 months of life while the infant immune system has diminished ability to generate somatically mutated antibodies.[25,59] Because the bispecific ai-mAb is antigenically distinct from RSV, it may circumvent interference of the infant immune response caused by epitope masking and antigen clearance mediated by maternal antibodies.[27,28,30] The strategy to elicit high titers of anti-F antibodies in pregnant people may prove to be a double-edged sword in this regard; high titers will provide transient protection to infants while at the same time hampering their humoral response to vaccination. Vaccination of infants with an antigenically disparate ai-mAb could augment protection afforded by maternal vaccination or prophylactic mAb transfer by eliciting a *de novo* serum neutralizing response that persists beyond the point when transferred antibodies no longer provide protection.

Collectively, this highlights potential advantages of an ai-mAb-based vaccine strategy as it can selectively target a B cell class pre-programmed to bind a conserved antigenic site on preF and neutralize RSV without the need for affinity maturation, and it is

antigenically distinct from RSV, circumventing epitope masking by maternal antibodies. The bispecific ai-mAb described here represents a step toward a safe and effective infant vaccine to prevent severe RSV infection.

Limitations

Our analysis was restricted to *in vitro* and *ex vivo* investigation. Although the calcium flux assays here indicate that the ai-mAb can activate target cells of interest, these experiments cannot predict the fate of VH3-21/VL1-40 B cells following immunization *in vivo.* The development of a small animal model that expresses target VH3-21/VL1-40 BCRs at relevant frequencies would enable *in vivo* analysis in the future. This would further enable us to determine the fate of activated B cells, their BCRs, and the antibodies they secrete and whether protective levels of serum antibodies can be achieved by ai-mAb immunization.

Another limitation of this study was the limited number of donors screened with the bispecific ai-mAb in sorting studies (n = 3), and the corresponding number of unmutated VH3-21/VL1-40 mAbs assayed for binding and neutralization potential.

RESOURCE AVAILABILITY

Lead contact

Requests for resources and reagents should be directed to and will be fulfilled by Andrew T. McGuire (amcguire@fredhutch.org).

Materials availability

All materials generated here are available upon request under a material transfer agreement from the corresponding author (amcguire@fredhutch.org). The pTT3 vectors are used under license from the National Research Council of Canada.

Data and code availability

- Data presented in this study are deposited in the following databases. Coordinates and structure factors for both ADI-19425 Fab–1D3 Fab and ADI-19425 Fab–2C1 Fab structures have been deposited in the Protein Data Bank (PDB) under accession codes 8VS8 (ADI-19425 Fab–1D3 Fab) and 8VS7 (ADI-19425 Fab–2C1 Fab). In addition, the following publicly available datasets are mentioned: PDB: 1D5I,[60] 5I76,[61] 6P67,[62] 6APC,[11] 6APD,[9] and 5TDG.[63] Accession numbers for produced VH3-21/VL1-40 mAbs can be found in Table S2. All other data are available upon request from the lead contact.
- This paper does not report original code.
- Any additional information required to reanalyze the data reported in this work is available from the lead contact upon request.

ACKNOWLEDGMENTS

We thank the study participants who provided the PBMC samples that were used in this study. This research was supported by NIAID R21AI156063 (awarded to A.T.M.) as well as The Bill and Melinda Gates Foundation INV-036995 (awarded to A.E.). This research was also supported by the Genomics & Bioinformatics (RRID: SCR_022606), Flow Cytometry (RRID: SCR_022613), Antibody Technology (RRID: SCR_022608), and Electron Microscopy (RRID: SCR_022611) Shared Resources of the Fred Hutchinson Cancer Center/University of Washington/Seattle Children's Cancer Consortium (P30 CA015704).

We thank the J.B. Pendleton Charitable Trust for its generous support of Formulatrix robotic instruments. We thank Stephen Voght for assistance with writing this manuscript. We thank Yehudi Bloch for helping solve the ADI19425-1D3 complex structure. X-ray diffraction data were collected at the Berkeley Center for Structural Biology beamline 5.0.2. The Berkeley Center for Structural Biology is supported in part by the Howard Hughes Medical Institute. The Advanced Light Source is a Department of Energy Office of Science User Facility under contract DE-AC02-05CH11231. The Pilatus detector on 5.0.2. was funded under National Institutes of Health grant S10 OD021832. The ALS-ENABLE beamlines are supported in part by the National Institutes of Health, National Institute of General Medical Sciences grant P30 GM124169. X-ray diffraction data were also derived from work performed at Argonne National Laboratory (ANL), Structural Biology Center (SBC), ID-19 at the Advance Photon Source (APS) under US Department of Energy, Office of Biological and Environmental Research contract DE-AC02-06CH11357. The content is solely the responsibility of the authors and does not necessarily represent the official views of the National Institutes of Health. BioRender was used to create the graphical abstract for this manuscript.

AUTHOR CONTRIBUTIONS

Conceptualization, A.T.M. and S.C.S.; investigation, S.C.S., Y.-H.W., L.J.H., G.K., A.M.H., B.P., I.R.S., N.A., A.P., M.B., C.B.C., and M.B.P.; formal analysis, S.C.S., Y.-H.W., L.J.H., G.K., A.M.H., B.P., I.R.S., Z.M., and A.T.M.; writing – original draft, S.C.S., L.J.H., G.K., A.M.H., M.P., and A.T.M.; writing – review and editing, all authors; visualization, S.C.S., G.K., A.M.H., and A.T.M.; supervision, A.T.M., J.B., M.B.P., A.E., and M.J.M.; funding acquisition, A.T.M., M.B.P., and A.E.

DECLARATION OF INTERESTS

A.T.M. and S.C.S. are co-inventors on a patent application 63/635,785 pertaining to the ai-mAbs described here.

STAR★METHODS

Detailed methods are provided in the online version of this paper and include the following:

- KEY RESOURCES TABLE
- EXPERIMENTAL MODEL AND STUDY PARTICIPANT DETAILS
  - Human subjects
  - Cell lines
- METHOD DETAILS
  - Recombinant antibodies
  - Generation of anti-idiotypic monoclonal antibodies
  - ELISA screening of anti-idiotypic antibodies
  - Recombinant protein expression
  - Generation of a VH3-21/VL1-40 targeting bispecific ai-mAb
  - B cell sorting
  - Biolayer interferometry
  - Generation of B cell lines
  - Ai-mAb staining of BCR-transduced cell lines
  - B cell activation in BCR-transduced cell lines
  - Fab-Fab complex formation
  - Crystallization and data collection
  - Structure solution, refinement, and analysis
  - Negative stain electron microscopy and analysis
  - Virus production
  - Plaque reduction neutralization assays
  - Plaque reduction neutralization competition assays
- QUANTIFICATION AND STATISTICAL ANALYSIS

SUPPLEMENTAL INFORMATION

Supplemental information can be found online at https://doi.org/10.1016/j.celrep.2024.114811.

Received: April 5, 2024
Revised: July 23, 2024
Accepted: September 16, 2024
Published: October 8, 2024

REFERENCES

1. Shi, T., McAllister, D.A., O'Brien, K.L., Simoes, E.A.F., Madhi, S.A., Gessner, B.D., Polack, F.P., Balsells, E., Acacio, S., Aguayo, C., et al. (2017). Global, regional, and national disease burden estimates of acute lower respiratory infections due to respiratory syncytial virus in young children in 2015: a systematic review and modelling study. Lancet (London, England) *390*, 946–958. https://doi.org/10.1016/S0140-6736(17)30938-8.
2. Lozano, R., Naghavi, M., Foreman, K., Lim, S., Shibuya, K., Aboyans, V., Abraham, J., Adair, T., Aggarwal, R., Ahn, S.Y., et al. (2012). Global and regional mortality from 235 causes of death for 20 age groups in 1990 and 2010: a systematic analysis for the Global Burden of Disease Study 2010. Lancet (London, England) *380*, 2095–2128. https://doi.org/10.1016/S0140-6736(12)61728-0.
3. Widmer, K., Griffin, M.R., Zhu, Y., Williams, J.V., and Talbot, H.K. (2014). Respiratory syncytial virus- and human metapneumovirus-associated emergency department and hospital burden in adults. Influenza Other Respir. Viruses *8*, 347–352. https://doi.org/10.1111/irv.12234.
4. Hall, C.B., Weinberg, G.A., Iwane, M.K., Blumkin, A.K., Edwards, K.M., Staat, M.A., Auinger, P., Griffin, M.R., Poehling, K.A., Erdman, D., et al. (2009). The burden of respiratory syncytial virus infection in young children. N. Engl. J. Med. *360*, 588–598. https://doi.org/10.1056/NEJMoa0804877.
5. Gilman, M.S.A., Castellanos, C.A., Chen, M., Ngwuta, J.O., Goodwin, E., Moin, S.M., Mas, V., Melero, J.A., Wright, P.F., Graham, B.S., et al. (2016). Rapid profiling of RSV antibody repertoires from the memory B cells of naturally infected adult donors. Sci. Immunol. *1*, eaaj1879. https://doi.org/10.1126/sciimmunol.aaj1879.
6. Ngwuta, J.O., Chen, M., Modjarrad, K., Joyce, M.G., Kanekiyo, M., Kumar, A., Yassine, H.M., Moin, S.M., Killikelly, A.M., Chuang, G.Y., et al. (2015). Prefusion F-specific antibodies determine the magnitude of RSV neutralizing activity in human sera. Sci. Transl. Med. *7*, 309ra162. https://doi.org/10.1126/scitranslmed.aac4241.
7. Ruckwardt, T.J., Morabito, K.M., and Graham, B.S. (2019). Immunological Lessons from Respiratory Syncytial Virus Vaccine Development. Immunity *51*, 429–442. https://doi.org/10.1016/j.immuni.2019.08.007.
8. Battles, M.B., and McLellan, J.S. (2019). Respiratory syncytial virus entry and how to block it. Nat. Rev. Microbiol. *17*, 233–245. https://doi.org/10.1038/s41579-019-0149-x.
9. Goodwin, E., Gilman, M.S.A., Wrapp, D., Chen, M., Ngwuta, J.O., Moin, S.M., Bai, P., Sivasubramanian, A., Connor, R.I., Wright, P.F., et al. (2018). Infants Infected with Respiratory Syncytial Virus Generate Potent Neutralizing Antibodies that Lack Somatic Hypermutation. Immunity *48*, 339–349.e5. https://doi.org/10.1016/j.immuni.2018.01.005.
10. Hammitt, L.L., Dagan, R., Yuan, Y., Baca Cots, M., Bosheva, M., Madhi, S.A., Muller, W.J., Zar, H.J., Brooks, D., Grenham, A., et al. (2022). Nirsevimab for Prevention of RSV in Healthy Late-Preterm and Term Infants. N. Engl. J. Med. *386*, 837–846. https://doi.org/10.1056/NEJMoa2110275.
11. Corti, D., Bianchi, S., Vanzetta, F., Minola, A., Perez, L., Agatic, G., Guarino, B., Silacci, C., Marcandalli, J., Marsland, B.J., et al. (2013). Cross-neutralization of four paramyxoviruses by a human monoclonal antibody. Nature *501*, 439–443. https://doi.org/10.1038/nature12442.
12. Kwakkenbos, M.J., Diehl, S.A., Yasuda, E., Bakker, A.Q., van Geelen, C.M.M., Lukens, M.V., van Bleek, G.M., Widjojoatmodjo, M.N., Bogers, W.M.J.M., Mei, H., et al. (2010). Generation of stable monoclonal antibody-producing B cell receptor-positive human memory B cells by genetic programming. Nat. Med. *16*, 123–128. https://doi.org/10.1038/nm.2071.
13. McLellan, J.S., Chen, M., Leung, S., Graepel, K.W., Du, X., Yang, Y., Zhou, T., Baxa, U., Yasuda, E., Beaumont, T., et al. (2013). Structure of RSV fusion glycoprotein trimer bound to a prefusion-specific neutralizing antibody. Science *340*, 1113–1117. https://doi.org/10.1126/science.1234914.
14. Wen, X., Mousa, J.J., Bates, J.T., Lamb, R.A., Crowe, J.E., Jr., and Jardetzky, T.S. (2017). Structural basis for antibody cross-neutralization of respiratory syncytial virus and human metapneumovirus. Nat. Microbiol. *2*, 16272. https://doi.org/10.1038/nmicrobiol.2016.272.
15. McLellan, J.S., Chen, M., Joyce, M.G., Sastry, M., Stewart-Jones, G.B.E., Yang, Y., Zhang, B., Chen, L., Srivatsan, S., Zheng, A., et al. (2013). Structure-based design of a fusion glycoprotein vaccine for respiratory syncytial virus. Science *342*, 592–598. https://doi.org/10.1126/science.1243283.
16. Kampmann, B., Madhi, S.A., Munjal, I., Simões, E.A.F., Pahud, B.A., Llapur, C., Baker, J., Pérez Marc, G., Radley, D., Shittu, E., et al. (2023). Bivalent Prefusion F Vaccine in Pregnancy to Prevent RSV Illness in Infants. N. Engl. J. Med. *388*, 1451–1464. https://doi.org/10.1056/NEJMoa2216480.
17. The IMpact-RSV Study Group (1998). Humanized Respiratory Syncytial Virus Monoclonal Antibody, Reduces Hospitalization From Respiratory Syncytial Virus Infection in High-risk Infants. Pediatrics *102*, 531–537.
18. Homaira, N., Rawlinson, W., Snelling, T.L., and Jaffe, A. (2014). Effectiveness of Palivizumab in Preventing RSV Hospitalization in High Risk Children: A Real-World Perspective. Int. J. Pediatr. *2014*, 571609. https://doi.org/10.1155/2014/571609.
19. Muller, W.J., Madhi, S.A., Seoane Nuñez, B., Baca Cots, M., Bosheva, M., Dagan, R., Hammitt, L.L., Llapur, C.J., Novoa, J.M., Saez Llorens, X., et al. (2023). Nirsevimab for Prevention of RSV in Term and Late-Preterm Infants. N. Engl. J. Med. *388*, 1533–1534. https://doi.org/10.1056/NEJMc2214773.
20. (2023). FDA Approves First Vaccine for Pregnant Individuals to Prevent RSV in Infants. In F.O.o.M. AffairsF.O.o.M. Affairs (FDA). https://www.fda.gov/news-events/press-announcements/fda-approves-first-vaccine-pregnant-individuals-prevent-rsv-infants.
21. Killikelly, A.M., Kanekiyo, M., and Graham, B.S. (2016). Pre-fusion F is absent on the surface of formalin-inactivated respiratory syncytial virus. Sci. Rep. *6*, 34108. https://doi.org/10.1038/srep34108.
22. KIM, H.W., CANCHOLA, J.G., BRANDT, C.D., PYLES, G., CHANOCK, R.M., JENSEN, K., and PARROTT, R.H. (1969). RESPIRATORY SYNCYTIAL VIRUS DISEASE IN INFANTS DESPITE PRIOR ADMINISTRATION OF ANTIGENIC INACTIVATED VACCINE12. Am. J. Epidemiol. *89*, 422–434. https://doi.org/10.1093/oxfordjournals.aje.a120955.
23. KAPIKIAN, A.Z., MITCHELL, R.H., CHANOCK, R.M., SHVEDOFF, R.A., and STEWART, C.E. (1969). AN EPIDEMIOLOGIC STUDY OF ALTERED CLINICAL REACTIVITY TO RESPIRATORY SYNCYTIAL (RS) VIRUS INFECTION IN CHILDREN PREVIOUSLY VACCINATED WITH AN INACTIVATED RS VIRUS VACCINE. Am. J. Epidemiol. *89*, 405–421. https://doi.org/10.1093/oxfordjournals.aje.a120954.
24. Prelog, M. (2013). Differential approaches for vaccination from childhood to old age. Gerontology *59*, 230–239. https://doi.org/10.1159/000343475.
25. Siegrist, C.-A., and Aspinall, R. (2009). B-cell responses to vaccination at the extremes of age. Nat. Rev. Immunol. *9*, 185–194. https://doi.org/10.1038/nri2508.
26. Bates, J.T., Keefer, C.J., Utley, T.J., Correia, B.E., Schief, W.R., and Crowe, J.E. (2013). Reversion of somatic mutations of the respiratory syncytial virus-specific human monoclonal antibody Fab19 reveal a direct relationship between association rate and neutralizing potency. J. Immunol. *190*, 3732–3739. https://doi.org/10.4049/jimmunol.1202964.
27. Siegrist, C.A. (2003). Mechanisms by which maternal antibodies influence infant vaccine responses: review of hypotheses and definition of main determinants. Vaccine *21*, 3406–3412. https://doi.org/10.1016/s0264-410x(03)00342-6.
28. Vono, M., Eberhardt, C.S., Auderset, F., Mastelic-Gavillet, B., Lemeille, S., Christensen, D., Andersen, P., Lambert, P.-H., and Siegrist, C.-A. (2019). Maternal Antibodies Inhibit Neonatal and Infant Responses to Vaccination by Shaping the Early-Life B Cell Repertoire within Germinal Centers. Cell Rep. *28*, 1773–1784.e5. https://doi.org/10.1016/j.celrep.2019.07.047.
29. Shinoff, J.J., O'Brien, K.L., Thumar, B., Shaw, J.B., Reid, R., Hua, W., Santosham, M., and Karron, R.A. (2008). Young Infants Can Develop Protective Levels of Neutralizing Antibody after Infection with Respiratory Syncytial Virus. J. Infect. Dis. *198*, 1007–1015. https://doi.org/10.1086/591460.

30. Murphy, B.R., Graham, B.S., Prince, G.A., Walsh, E.E., Chanock, R.M., Karzon, D.T., and Wright, P.F. (1986). Serum and nasal-wash immunoglobulin G and A antibody response of infants and children to respiratory syncytial virus F and G glycoproteins following primary infection. J. Clin. Microbiol. *23*, 1009–1014. https://doi.org/10.1128/jcm.23.6.1009-1014.1986.

31. Avnir, Y., Prachanronarong, K.L., Zhang, Z., Hou, S., Peterson, E.C., Sui, J., Zayed, H., Kurella, V.B., McGuire, A.T., Stamatatos, L., et al. (2017). Structural Determination of the Broadly Reactive Anti-IGHV1-69 Anti-idiotypic Antibody G6 and Its Idiotope. Cell Rep. *21*, 3243–3255. https://doi.org/10.1016/j.celrep.2017.11.056.

32. Avnir, Y., Tallarico, A.S., Zhu, Q., Bennett, A.S., Connelly, G., Sheehan, J., Sui, J., Fahmy, A., Huang, C.Y., Cadwell, G., et al. (2014). Molecular signatures of hemagglutinin stem-directed heterosubtypic human neutralizing antibodies against influenza A viruses. PLoS Pathog. *10*, e1004103. https://doi.org/10.1371/journal.ppat.1004103.

33. Dosenovic, P., Pettersson, A.-K., Wall, A., Thientosapol, E.S., Feng, J., Weidle, C., Bhullar, K., Kara, E.E., Hartweger, H., Pai, J.A., et al. (2019). Anti-idiotypic antibodies elicit anti-HIV-1–specific B cell responses. J. Exp. Med. *216*, 2316–2330. https://doi.org/10.1084/jem.20190446.

34. Bancroft, T., DeBuysscher, B.L., Weidle, C., Schwartz, A., Wall, A., Gray, M.D., Feng, J., Steach, H.R., Fitzpatrick, K.S., Gewe, M.M., et al. (2019). Detection and activation of HIV broadly neutralizing antibody precursor B cells using anti-idiotypes. J. Exp. Med. *216*, 2331–2347. https://doi.org/10.1084/jem.20190164.

35. Seydoux, E., Wan, Y.H., Feng, J., Wall, A., Aljedani, S., Homad, L.J., MacCamy, A.J., Weidle, C., Gray, M.D., Brumage, L., et al. (2021). Development of a VRC01-class germline targeting immunogen derived from anti-idiotypic antibodies. Cell Rep. *35*, 109084. https://doi.org/10.1016/j.celrep.2021.109084.

36. Snijder, J., Ortego, M.S., Weidle, C., Stuart, A.B., Gray, M.D., McElrath, M.J., Pancera, M., Veesler, D., and McGuire, A.T. (2018). An Antibody Targeting the Fusion Machinery Neutralizes Dual-Tropic Infection and Defines a Site of Vulnerability on Epstein-Barr Virus. Immunity *48*, 799–811.e9. https://doi.org/10.1016/j.immuni.2018.03.026.

37. DeKosky, B.J., Lungu, O.I., Park, D., Johnson, E.L., Charab, W., Chrysostomou, C., Kuroda, D., Ellington, A.D., Ippolito, G.C., Gray, J.J., and Georgiou, G. (2016). Large-scale sequence and structural comparisons of human naive and antigen-experienced antibody repertoires. Proc. Natl. Acad. Sci. USA *113*, E2636–E2645. https://doi.org/10.1073/pnas.1525510113.

38. Fong, Y., Huang, Y., Gilbert, P.B., and Permar, S.R. (2017). chngpt: threshold regression model estimation and inference. BMC Bioinf. *18*, 454. https://doi.org/10.1186/s12859-017-1863-x.

39. Moore, G.L., Bernett, M.J., Rashid, R., Pong, E.W., Nguyen, D.H.T., Jacinto, J., Eivazi, A., Nisthal, A., Diaz, J.E., Chu, S.Y., et al. (2019). A robust heterodimeric Fc platform engineered for efficient development of bispecific antibodies of multiple formats. Methods *154*, 38–50. https://doi.org/10.1016/j.ymeth.2018.10.006.

40. Tiller, T., Meffre, E., Yurasov, S., Tsuiji, M., Nussenzweig, M.C., and Wardemann, H. (2008). Efficient generation of monoclonal antibodies from single human B cells by single cell RT-PCR and expression vector cloning. J. Immunol. Methods *329*, 112–124. https://doi.org/10.1016/j.jim.2007.09.017.

41. Brochet, X., Lefranc, M.P., and Giudicelli, V. (2008). IMGT/V-QUEST: the highly customized and integrated system for IG and TR standardized V-J and V-D-J sequence analysis. Nucleic Acids Res. *36*, W503–W508. https://doi.org/10.1093/nar/gkn316.

42. Giudicelli, V., Brochet, X., and Lefranc, M.P. (2011). IMGT/V-QUEST: IMGT standardized analysis of the immunoglobulin (IG) and T cell receptor (TR) nucleotide sequences. Cold Spring Harb. Protoc. *2011*, 695–715. https://doi.org/10.1101/pdb.prot5633.

43. McLellan, J.S., Yang, Y., Graham, B.S., and Kwong, P.D. (2011). Structure of respiratory syncytial virus fusion glycoprotein in the postfusion conformation reveals preservation of neutralizing epitopes. J. Virol. *85*, 7788–7796. https://doi.org/10.1128/JVI.00555-11.

44. Cabán, M., Rodarte, J.V., Bibby, M., Gray, M.D., Taylor, J.J., Pancera, M., and Boonyaratanakornkit, J. (2023). Cross-protective antibodies against common endemic respiratory viruses. Nat. Commun. *14*, 798. https://doi.org/10.1038/s41467-023-36459-3.

45. Joyce, M.G., Bao, A., Chen, M., Georgiev, I.S., Ou, L., Bylund, T., Druz, A., Kong, W.P., Peng, D., Rundlet, E.J., et al. (2019). Crystal Structure and Immunogenicity of the DS-Cav1-Stabilized Fusion Glycoprotein From Respiratory Syncytial Virus Subtype B. Pathog. Immun. *4*, 294–323. https://doi.org/10.20411/pai.v4i2.338.

46. Jardine, J.G., Kulp, D.W., Havenar-Daughton, C., Sarkar, A., Briney, B., Sok, D., Sesterhenn, F., Ereño-Orbea, J., Kalyuzhniy, O., Deresa, I., et al. (2016). HIV-1 broadly neutralizing antibody precursor B cells revealed by germline-targeting immunogen. Science *351*, 1458–1463. https://doi.org/10.1126/science.aad9195.

47. Havenar-Daughton, C., Sarkar, A., Kulp, D.W., Toy, L., Hu, X., Deresa, I., Kalyuzhniy, O., Kaushik, K., Upadhyay, A.A., Menis, S., et al. (2018). The human naive B cell repertoire contains distinct subclasses for a germline-targeting HIV-1 vaccine immunogen. Sci. Transl. Med. *10*, eaat0381. https://doi.org/10.1126/scitranslmed.aat0381.

48. Leggat, D.J., Cohen, K.W., Willis, J.R., Fulp, W.J., deCamp, A.C., Kalyuzhniy, O., Cottrell, C.A., Menis, S., Finak, G., Ballweber-Fleming, L., et al. (2022). Vaccination induces HIV broadly neutralizing antibody precursors in humans. Science *378*, eadd6502. https://doi.org/10.1126/science.add6502.

49. Hoot, S., McGuire, A.T., Cohen, K.W., Strong, R.K., Hangartner, L., Klein, F., Diskin, R., Scheid, J.F., Sather, D.N., Burton, D.R., and Stamatatos, L. (2013). Recombinant HIV envelope proteins fail to engage germline versions of anti-CD4bs bNAbs. PLoS Pathog. *9*, e1003106. https://doi.org/10.1371/journal.ppat.1003106.

50. McGuire, A.T., Dreyer, A.M., Carbonetti, S., Lippy, A., Glenn, J., Scheid, J.F., Mouquet, H., and Stamatatos, L. (2014). HIV antibodies. Antigen modification regulates competition of broad and narrow neutralizing HIV antibodies. Science *346*, 1380–1383. https://doi.org/10.1126/science.1259206.

51. Olia, A.S., Prabhakaran, M., Harris, D.R., Cheung, C.S.F., Gillespie, R.A., Gorman, J., Hoover, A., Morano, N.C., Ourahmane, A., Srikanth, A., et al. (2024). Anti-idiotype isolation of a broad and potent influenza A virus-neutralizing human antibody. Front. Immunol. *15*, 1399960. https://doi.org/10.3389/fimmu.2024.1399960.

52. Lee, J.H., Toy, L., Kos, J.T., Safonova, Y., Schief, W.R., Havenar-Daughton, C., Watson, C.T., and Crotty, S. (2021). Vaccine genetics of IGHV1-2 VRC01-class broadly neutralizing antibody precursor naïve human B cells. NPJ Vaccines *6*, 113. https://doi.org/10.1038/s41541-021-00376-7.

53. Mas, V., Nair, H., Campbell, H., Melero, J.A., and Williams, T.C. (2018). Antigenic and sequence variability of the human respiratory syncytial virus F glycoprotein compared to related viruses in a comprehensive dataset. Vaccine *36*, 6660–6673. https://doi.org/10.1016/j.vaccine.2018.09.056.

54. Wen, X., Suryadevara, N., Kose, N., Liu, J., Zhan, X., Handal, L.S., Williamson, L.E., Trivette, A., Carnahan, R.H., Jardetzky, T.S., and Crowe, J.E. (2023). Potent cross-neutralization of respiratory syncytial virus and human metapneumovirus through a structurally conserved antibody recognition mode. Cell Host Microbe *31*, 1288–1300.e6. https://doi.org/10.1016/j.chom.2023.07.002.

55. Paus, D., Phan, T.G., Chan, T.D., Gardam, S., Basten, A., and Brink, R. (2006). Antigen recognition strength regulates the choice between extrafollicular plasma cell and germinal center B cell differentiation. J. Exp. Med. *203*, 1081–1091. https://doi.org/10.1084/jem.20060087.

56. Sutton, H.J., Gao, X., Kelly, H.G., Parker, B.J., Lofgren, M., Dacon, C., Chatterjee, D., Seder, R.A., Tan, J., Idris, A.H., et al. (2024). Lack of affinity signature for germinal center cells that have initiated plasma cell differentiation. Immunity *57*, 245–255.e5. https://doi.org/10.1016/j.immuni.2023.12.010.

57. MacLennan, I.C.M., Toellner, K.M., Cunningham, A.F., Serre, K., Sze, D.M.Y., Zúñiga, E., Cook, M.C., and Vinuesa, C.G. (2003). Extrafollicular antibody responses. Immunol. Rev. *194*, 8–18. https://doi.org/10.1034/j.1600-065x.2003.00058.x.

58. Chan, T.D., Gatto, D., Wood, K., Camidge, T., Basten, A., and Brink, R. (2009). Antigen affinity controls rapid T-dependent antibody production by driving the expansion rather than the differentiation or extrafollicular migration of early plasmablasts. J. Immunol. *183*, 3139–3149. https://doi.org/10.4049/jimmunol.0901690.

59. Ridings, J., Dinan, L., Williams, R., Roberton, D., and Zola, H. (1998). Somatic mutation of immunoglobulin V(H)6 genes in human infants. Clin. Exp. Immunol. *114*, 33–39. https://doi.org/10.1046/j.1365-2249.1998.00694.x.

60. Wu, X., Yang, Z.Y., Li, Y., Hogerkorp, C.M., Schief, W.R., Seaman, M.S., Zhou, T., Schmidt, S.D., Wu, L., Xu, L., et al. (2010). Rational design of envelope identifies broadly neutralizing human monoclonal antibodies to HIV-1. Science *329*, 856–861. https://doi.org/10.1126/science.1187659.

61. Balachandran, N., Oba, D.E., and Hutt-Fletcher, L.M. (1987). Antigenic cross-reactions among herpes simplex virus types 1 and 2, Epstein-Barr virus, and cytomegalovirus. J. Virol. *61*, 1125–1135. https://doi.org/10.1128/JVI.61.4.1125-1135.1987.

62. Minor, W., Cymborowski, M., Otwinowski, Z., and Chruszcz, M. (2006). HKL-3000: the integration of data reduction and structure solution–from diffraction images to an initial model in minutes. Acta Crystallogr. D Biol. Crystallogr. *62*, 859–866. https://doi.org/10.1107/S0907444906019949.

63. Zhang, B., Chen, L., Silacci, C., Thom, M., Boyington, J.C., Druz, A., Joyce, M.G., Guzman, E., Kong, W.P., Lai, Y.T., et al. (2017). Protection of calves by a prefusion-stabilized bovine RSV F vaccine. NPJ Vaccines *2*, 7. https://doi.org/10.1038/s41541-017-0005-9.

64. Adams, P.D., Afonine, P.V., Bunkóczi, G., Chen, V.B., Davis, I.W., Echols, N., Headd, J.J., Hung, L.W., Kapral, G.J., Grosse-Kunstleve, R.W., et al. (2010). PHENIX: a comprehensive Python-based system for macromolecular structure solution. Acta Crystallogr. D Biol. Crystallogr. *66*, 213–221. https://doi.org/10.1107/s0907444909052925.

65. Emsley, P., and Cowtan, K. (2004). Coot: model-building tools for molecular graphics. Acta Crystallogr. D Biol. Crystallogr. *60*, 2126–2132. https://doi.org/10.1107/s0907444904019158.

66. Emsley, P., Lohkamp, B., Scott, W.G., and Cowtan, K. (2010). Features and development of Coot. Acta Crystallogr. D Biol. Crystallogr. *66*, 486–501. https://doi.org/10.1107/S0907444910007493.

67. Krissinel, E., and Henrick, K. (2007). Inference of macromolecular assemblies from crystalline state. J. Mol. Biol. *372*, 774–797. https://doi.org/10.1016/j.jmb.2007.05.022.

68. Krissinel, E., and Henrick, K. (2007). Protein interfaces, surfaces and assemblies service PISA at European Bioinformatics Institute. J. Mol. Biol. 372, 774–797.

69. Suloway, C., Pulokas, J., Fellmann, D., Cheng, A., Guerra, F., Quispe, J., Stagg, S., Potter, C.S., and Carragher, B. (2005). Automated molecular microscopy: the new Leginon system. J. Struct. Biol. *151*, 41–60. https://doi.org/10.1016/j.jsb.2005.03.010.

70. Punjani, A., Rubinstein, J.L., Fleet, D.J., and Brubaker, M.A. (2017). cryoSPARC: algorithms for rapid unsupervised cryo-EM structure determination. Nat. Methods *14*, 290–296. https://doi.org/10.1038/nmeth.4169.

71. Meng, E.C., Goddard, T.D., Pettersen, E.F., Couch, G.S., Pearson, Z.J., Morris, J.H., and Ferrin, T.E. (2023). UCSF ChimeraX: Tools for structure building and analysis. Protein Sci. *32*, e4792. https://doi.org/10.1002/pro.4792.

72. Meyer, L., López, T., Espinosa, R., Arias, C.F., Vollmers, C., and DuBois, R.M. (2019). A simplified workflow for monoclonal antibody sequencing. PLoS One *14*, e0218717. https://doi.org/10.1371/journal.pone.0218717.

73. Jaffe, D.B., Shahi, P., Adams, B.A., Chrisman, A.M., Finnegan, P.M., Raman, N., Royall, A.E., Tsai, F., Vollbrecht, T., Reyes, D.S., and McDonnell, W.J. (2022). enclone: precision clonotyping and analysis of immune receptors. Preprint at bioRxiv. https://doi.org/10.1101/2022.04.21.489084.

74. Sathiyamoorthy, K., Hu, Y.X., Möhl, B.S., Chen, J., Longnecker, R., and Jardetzky, T.S. (2016). Structural basis for Epstein-Barr virus host cell tropism mediated by gp42 and gHgL entry glycoproteins. Nat. Commun. *7*, 13557. https://doi.org/10.1038/ncomms13557.

75. McGuire, A.T., Hoot, S., Dreyer, A.M., Lippy, A., Stuart, A., Cohen, K.W., Jardine, J., Menis, S., Scheid, J.F., West, A.P., et al. (2013). Engineering HIV envelope protein to activate germline B cell receptors of broadly neutralizing anti-CD4 binding site antibodies. J. Exp. Med. *210*, 655–663. https://doi.org/10.1084/jem.20122824.

76. Kabsch, W. (2010). XDS. Acta Crystallogr. D Biol. Crystallogr. *66*, 125–132. https://doi.org/10.1107/s0907444909047337.

77. Mundorff, E.C., Hanson, M.A., Varvak, A., Ulrich, H., Schultz, P.G., and Stevens, R.C. (2000). Conformational effects in biological catalysis: an antibody-catalyzed oxy-cope rearrangement. Biochemistry *39*, 627–632. https://doi.org/10.1021/bi9924314.

78. Kim, J.H., Sim, D.W., Park, D., Jung, T.G., Lee, S., Oh, T., Ha, J.R., Seok, S.H., Seo, M.D., Kang, H.C., et al. (2016). Bacterial production and structure-functional validation of a recombinant antigen-binding fragment (Fab) of an anti-cancer therapeutic antibody targeting epidermal growth factor receptor. Appl. Microbiol. Biotechnol. *100*, 10521–10529. https://doi.org/10.1007/s00253-016-7717-z.

79. Hixon, J.A., Andrews, C., Kashi, L., Kohnhorst, C.L., Senkevitch, E., Czarra, K., Barata, J.T., Li, W., Schneider, J.P., Walsh, S.T.R., and Durum, S.K. (2020). New anti-IL-7Rα monoclonal antibodies show efficacy against T cell acute lymphoblastic leukemia in pre-clinical models. Leukemia *34*, 35–49. https://doi.org/10.1038/s41375-019-0531-8.

80. DeLano, W.L. (2002). The PyMOL molecular graphics system. http://www.pymol.org/.

81. Harrington, B., and Engelen, J. (2004). Inkscape. URL: http://www.inkscape.org.

STAR★METHODS

KEY RESOURCES TABLE

REAGENT or RESOURCE	SOURCE	IDENTIFIER
Antibodies		
Recombinant MLR Antibodies	This study	N/A
Recombinant ADI-19425	Goodwin et al.[9]	N/A
Recombinant ADI-14337	Goodwin et al.[9]	N/A
Recombinant ADI-25532	Goodwin et al.[9]	N/A
Recombinant AMMO1	Snijder et al.[36]	N/A
Recombinant AMMO1HC/ADI-19425LC	This study	N/A
Recombinant ADI-19425HC/CL40LC	This study	N/A
Recombinant ADI-14337HC/PHVP8LC	This study	N/A
Recombinant ADI-19425HC/PHVP8LC	This study	N/A
Recombinant AMMO1HC/ADI-14337LC	This study	N/A
Recombinant mVRC01	Wu et al.[60]	N/A
Recombinant E1D1	Balachandran et al.[61]	N/A
Recombinant D25	Kwakkenbos et al.[12]	N/A
Recombinant palivizumab	Homaira et al.[18]	N/A
Anti-human CD32	BD Biosciences	Cat# 551900; RRID:AB_39429
Anti-human CD16	BD Biosciences	Cat# 550383; RRID:AB_393649
Anti-human CD19-BV711	BioLegend	Cat# 302246; RRID:AB_2562065
Anti-human CD27-PE-Cy7	eBioscience	Cat# 25-0271-82; RRID:AB_1724035
Anti-human CD14-FITC	BD Pharmingen	Cat# 557153; RRID:AB_396589
Anti-human CD3-FITC	BD Pharmingen	Cat# 556611; RRID:AB_396484
Anti-human CD20-AF700	BioLegend	Cat# 302322; RRID:AB_493753
Anti-human IgD-PerCP-Cy5.5	eBioscience	Cat# 46-9868-42; RRID:AB_2573920
Anti-human IgM-BV605	BioLegend	Cat# 314524; RRID:AB_2562374
Anti-human CD14-PE	BD Pharmingen	Cat# 555398; RRID:AB_395799
Anti-human CD3-PE	BD Pharmingen	Cat# 556612; RRID:AB_396485
Anti-human Fcγ-PE	Jackson ImmunoResearch	Cat# 109-117-008; RRID:AB_2632442
Anti-IgG Fcγ	Jackson ImmunoResearch	Cat# 109-006-008; RRID:AB_2337546
Goat anti-mouse IgG, human ads-HRP	SouthernBiotech	Cat# 1030-05; RRID:AB_2619742
Bacterial and virus strains		
RSV subtype strain A2-GFP	ViraTree	Cat# RSV-GFP1
RSV subtype strain B1-GFP	ViraTree	Cat# RSVB-GFP3
Biological samples		
Cryopreserved PBMC samples	Collected from adults without HIV recruited at the Seattle HIV Vaccine Trials Unit, referred to as Seattle Assay Control (SAC) Cohort under the Fred Hutchinson Cancer Center IRB approved SAC protocol FHIRB0005567	N/A
Chemicals, peptides, and recombinant proteins		
EZ-Link NHS-PEG4-Biotin Kit	ThermoFisher Scientific	Cat# A39259
Endoproteinase Lys-C	New England BioLabs	Cat# P8109S
Methylcellulose	Sigma-Aldrich	Cat# M0387
Critical commercial assays		
Fixable Viability Dye-V500	eBioscience	Cat# 65-0866-14

(*Continued on next page*)

Continued		
REAGENT or RESOURCE	SOURCE	IDENTIFIER
Total SeqC-0960 APC streptavidin	BioLegend	Cat# 405157
Total SeqC-0959 APC streptavidin	BioLegend	Cat# 405159
Total SeqC-0958 APC streptavidin	BioLegend	Cat# 405293
Total SeqC-0957 APC streptavidi	BioLegend	Cat# 405285
Total SeqC-0956	BioLegend	Cat# 405293
Zenon Human IgG Labeling Kit - APC	ThermoFisher Scientific	Cat# Z25451
Zenon Human IgG Labeling Kit - PE	ThermoFisher Scientific	Cat# Z25455
Chromium Single Cell Human BCR Amplification Kit	10X Genomics	Cat# 1000253
5′ Feature Barcode Kit	10X Genomics	Cat# 1000256
Dual Index Kit TT Set A	10X Genomics	Cat# 1000215
Human B Cell Enrichment Kit	Stem Cell	Cat# 19054
DyLight 488 NHS Ester	Thermo Scientific	Cat# 46403
5x In-Fusion HD Enzyme Premix	Takara Bio	Cat# 639650
Pierce Protein G IgG Binding Buffer	ThermoFisher Scientific	Cat# 21011
Protein G Agarose	ThermoFisher Scientific	Cat# 15920010
Pierce IgG Elution Buffer	ThermoFisher Scientific	Cat# 21004
Monarch Total RNA Miniprep Kit	New England Biolabs	Cat# T2010S
Topo Cloning Kit	ThermoFisher Scientific	Cat# 450245
293 Transfection Reagent	EMD Millipore	Cat# 72181
Protein A resin	GoldBio	Cat# P-400-50
Ni-NTA resin	Thermo Scientific	Cat# 88221
Superscript IV	Invitrogen	Cat# 18091050
Platinum SuperFi II DNA Polymerase	Invitrogen	Cat# 12368050
Monarch Gel Extraction Kit	New England BioLabs	Cat# T1020S
Monarch PCR & DNA Clean Up Kit	New England BioLabs	Cat# T1030S
Fluo-4 Direct calcium indicator	Invitrogen	Cat# F10471
Clear Strategy I and II	Molecular Dimensions	N/A
ProPlex	Molecular Dimensions	N/A
MCSG1-3	Molecular Dimensions	N/A
WPS1 and 2	Rigaku	N/A
Xtal HT	Hampton Research	N/A
Additive screen	Hampton Reserach	N/A
Morpheus	Molecular Dimensions	N/A
SureBlue Reserve TMB Microwell Peroxidase Subtrate	SeraCare	Cat# 5120-0081
Deposited data		
VH3-21/VL1-40 antibody variable gene sequences	This study	GenBank: PP429379-PP429498
ADI-19425-2C1 complex	This study	PDB: 8VS7
ADI-19425-1C3 complex	This study	PDB: 8VS8
Experimental models: Cell lines		
293-6E cells	National Research Council, Canada	RRID: CVCL_HF20
293T cells	–	RRID:CVCL_0063
DG-75 cells	ATCC	Cat# CLR-2625; RRID:CVCL_0244
HEp-2 cells	ATCC	Cat# CCL-23; RRID:CVCL_1906
Vero cells	ATCC	Cat# CCL-81; RRID:CVCL_0059
Oligonucleotides		
Primers for antibody nested PCR and sequencing	Tiller et al.[40]	N/A

(*Continued on next page*)

Continued

REAGENT or RESOURCE	SOURCE	IDENTIFIER
Recombinant DNA		
pTT3 IgG1 expression vector with human constants	Snijder et al.[36]	N/A
pRSV-A preF (DS-Cav1)	McLellan et al.[15]	N/A
pRSV-A postF	McClellan et al.[43]	N/A
pRSV-B preF	Joyce et al.[45]	N/A
pChain A bispecific ai-mAb	This study, Moore et al.[39]	N/A
pChain B bispecific ai-mAb	This study, Moore et al.[39]	N/A
pTwist Lenti SFFV Puro WPRE-ADI-19425-BCR	This study	N/A
pTwist Lenti SFFV Puro WPRE-ADI-19425HC/AMMO1LC-BCR	This study	N/A
pTwist Lenti SFFV Puro WPRE-iv8HC/ADI-19425LC-BCR	This study	N/A
pTwist Lenti SFFV Puro WPRE-ADI-19470-BCR	This study	N/A
pTwist Lenti SFFV Puro WPRE-ADI-24811-BCR	This study	N/A
pTwist Lenti SFFV Puro WPRE-ADI-25542-BCR	This study	N/A
psPAX2	Addgene	Cat# 12260; RRID:Addgene_12260
pMD2.G	Addgene	Cat# 12259, RRID:Addgene_12259
Software and algorithms		
FlowJo 10.8.1 or later software package	Tree Star	N/A
Cell Ranger 3.0.0	10X Genomics	N/A
Enclone	10X Genomics	N/A
R Studio	R Foundation for Statistical Computing	N/A
chngpt	Fong et al.	N/A
IMGT V-quest	Bochet et al.	N/A
ForteBio BLI Analysis Software	Sartorious	N/A
Prism 8.0 or later software package	Graph Pad Software	N/A
XDS	Kabsch et al.	N/A
ALS Beamline 5.0.2	Lawrence Berkeley National Laboratory	N/A
Advanced Photon Source Beamline 19-ID	Advanced Photon Source	N/A
HKL3000	Minor et al.[62]	N/A
Phaser and Phenix	Adams et al.[64]	N/A
Coot	Emsley et al.,[65] Emsley et al.[66]	N/A
PyMOL	Delano	N/A
Inkscape	Harrington and Engelen	N/A
PISA	Krissinel,[67] Krissinel[68]	N/A
Leginon Software	Suloway et al.[69]	N/A
CryoSPARC v4.4.1	Punjani et al.[70]	N/A
ChimeraX	Meng et al.[71]	N/A
ImageJ	LOCI, University of Wisconsin	N/A
Other		
BD FACSymphony A5 and S6	BD Biosciences	N/A
NovaSeq	Illumina	N/A
NextSeq	Illumina	N/A
Clonepix	Molecular Devices	N/A
Amicon Filter Unit (15mLs)	EMD Millipore	Cat# UFC903024
HiLoad 16/600 Superdex S200	Millipore Sigma	Cat# GE28-9893-35

(*Continued on next page*)

Continued		
REAGENT or RESOURCE	SOURCE	IDENTIFIER
Bio-Rad NGC FPLC System	Bio-Rad	N/A
Enrich SEC 650	Bio-Rad	Cat# 7801650
Hi-Trap Q HP	Cytiva	Cat# 17115301
BD FACSAria II Cell Sorter	BD	N/A
Octet Red 96E	Sartorious	N/A
Anti-human IgG Capture Sensors (AHC)	Sartorious	Cat# 18-5060
Streptavidin Capture Sensors (SA)	Sartorious	Cat# 18-5019
Formulatrix NT8 Liquid Handler	Formulatrix	N/A
Rock Imager	Formulatrix	N/A
Talos L120C Transmission Electron Microscope	Talos	N/A
Typhoon Imager	GE Life Sciences	N/A
SpectraMax M2 Plate Reader	Molecular Devices	N/A

EXPERIMENTAL MODEL AND STUDY PARTICIPANT DETAILS

Human subjects

PBMC were collected from adults without HIV who were recruited at the Seattle HIV Vaccine Trials Unit (Seattle, Washington, USA) as part of the study "Establishing Immunologic Assays for Determining HIV-1 Prevention and Control", also referred to as Seattle Assay Control (SAC) Cohort. All participants signed informed consent, and the Fred Hutchinson Cancer Center (Seattle, Washington, USA) Institutional Review Board approved the SAC protocol (FHIRB0005567) prior to study initiation. Donors were selected randomly and no considerations were made for age or sex.

Cell lines

All cell lines were incubated at 37°C in the presence of 5% CO_2 and were not tested for mycoplasma contamination. 293-6E cells (human female) and suspension adapted 293T cells (human female) were maintained in Freestyle 293 media (ThermoFisher Cat# 12338018) with gentle shaking. DG-75 cells (human male) were maintained in RPMI (Corning Cat# 15-040-CV) + 10% FBS, 2 mM L-glutamine, 100 U/mL penicillin, and 100 μg/mL streptomycin (cRPMI). HEp-2 (human male) and Vero cells (African green monkey) were maintained in DMEM (Gibco Cat#11965-092) + 10% FBS, 100 U/mL penicillin, and 100 μg/mL streptomycin (cDMEM).

METHOD DETAILS

Recombinant antibodies

The heavy and light chain variable regions of ADI-19425 (VH GenBank: MG524063, VL GenBank: MG524528), ADI-25532 (VH GenBank: MG524182, VL GenBank: MG524647) and ADI-14437 (VH GenBank: MG524251, VL GenBank: MG524716) were codon optimized, cloned into pTT3-based IgG1 expression vectors with human constant regions[36] using 5x In-Fusion HD Enzyme Premix (Takara Bio) according to the manufacturer's instructions.

BCRs from VH3-21/VL1-40$^+$ B cells were expressed as recombinant antibodies as described above. Accession numbers for produced VH3-21/VL1-40 mAbs can be found in Table S2.

Generation of anti-idiotypic monoclonal antibodies

Mice were injected 3 or 5 times with a cocktail of ADI-19425, ADI-25532 and ADI-14337. Three days after the final injection, spleens were harvested and used to generate hybridomas at the Fred Hutchinson Antibody Technology Center. Hybridoma supernatants were initially screened for binding to a panel of mAbs including ADI-19425, ADI-25532, ADI-14337, the anti-EBV mAb AMMO1, a chimeric mAb with a VL1-40 light chain and a non-VH3-21 heavy chain (AMMO1-HC/ADI-19425LC) and a chimeric mAb with a VH3-21 heavy chain and a non-VL1-40 light chain (ADI-19425HC/CL40 light chain). Hybridomas from wells containing supernatant that showed a strong binding signal to ADI-19425, ADI-25532, ADI-14337 and weaker or no binding to the control mAbs were plated onto semi-solid media and single colonies were used to establish monoclonal cell lines using a Clonepix instrument. To purify mAbs, hybridomas were cultured in serum free media cells and cellular debris were removed by centrifugation at 4,000 × *g* followed by filtration through a 0.22 μm filter. The clarified supernatant was diluted with an equal volume of Pierce Protein G IgG Binding Buffer and then passed over a Protein G Agarose column, washed with 5 column volumes of Protein G IgG Binding Buffer and then eluted in 1 mL fractions of Pierce IgG Elution Buffer, pH 2.0 into 0.1 mL of Tris HCl, pH 8.0. Purified mAbs were then concentrated and buffer exchanged into PBS using an Amicon filter unit. Aliquots of purified mAbs were biotinylated

using the EZ-Link NHS-PEG4-Biotin kit at a theoretical 1:1 ratio of mAb to biotin. Purified mAbs were flash frozen and stored at −20°C until use.

To produce recombinant anti-idiotypic mAbs, RNA was extracted from 1 × 10^6 cells using the Monarch Total RNA Miniprep Kit and cDNA encoding the heavy and light chain variable regions of the murine hybridomas were by reverse transcribed and amplified using the procedures outlined in Meyer et al.[72] Amplicons were sanger sequenced directly or TOPO cloned and then sequenced. The sequences were codon optimized and cloned into pTT3-based IgG expression vectors with human constant regions[36] using 5x In-Fusion HD Enzyme Premix, expressed in 293-6E cells and purified using Protein A chromatography.

ELISA screening of anti-idiotypic antibodies

50 μL/well of target mAbs at 2μg/mL (ADI-19425, ADI-14337 etc.) in ELISA coating buffer (0.1 M NaHCO3 pH 9.4–9.6) were added to 96-well half volume plates (Thermo Scientific Cat# 3455) and incubated overnight at 4°C. The plates were then washed 3 times with ELISA wash buffer (PBS with 0.02% Tween 20). Plates were then blocked with 100 μL/well with ELISA blocking buffer (10% nonfat milk with 0.03% Tween 20) for 2 h at 37°C. After blocking, plates were washed 3 times with ELISA wash buffer. Followed washing, purified IgG from ai-mAb hybridoma supernatants were added to each well at 2μg/mL in 50ul in ELISA blocking buffer and plates were incubated for 1 h at 37°C. Following primary hybridoma ai-mAb staining, plates were washed 3 times with ELISA wash buffer and then stained with goat anti-mouse IgG human ads-HRP (Southern Biotech) at a 1:30,000 dilution in 50 μL/well of ELISA blocking buffer and incubated for 1 h at 37°C. Plates were then washed 3 times in ELISA wash buffer and developed with 50 μL/well of SureBlue Reserve TMB Microwell Peroxidase substrate (SeraCare) for 5 min, followed by neutralization with 50 μL/well of 1 N sulfuric acid. A_{450} of each well was read on a Molecular Devices SpectraMax M2 plate reader.

Recombinant protein expression

Plasmids encoding antibody heavy and light chains were transfected into 293-6E cells at a density of 10^6 cells/mL in Freestyle 293 media (ThermoFisher) using the 293Free transfection reagent (EMD Millipore) according to the manufacturer's instructions. Expression was carried out in Freestyle 293 media for 6 days after which cells and cellular debris were removed by centrifugation at 4,000 × *g* followed by filtration through a 0.22 μm filter. Clarified cell supernatant containing recombinant mAbs were passed over Protein A resin (GoldBio), pre-equilibrated with phosphate buffered saline (PBS; 1 mM KH_2PO_4, 155 mM NaCl, 3mM Na_2HPO_4) washed with 10 column volumes of PBS and then eluted in 1 mL aliquots of Pierce IgG Elution Buffer, pH 2.0 (ThermoFisher) into 0.1 mL of Tris HCl, pH 8.0. Purified antibodies were concentrated, and buffer exchanged into PBS using an Amicon filter unit.

Plasmids encoding RSV-A preF (DS-Cav1) and postF were kind gifts of Jason McLellan, and the plasmid encoding RSV strain B1 preF was a kind gift of Peter Kwong.[15,43,45] All RSV viral protein-encoding plasmids were transfected into 293-6E cells as described above. Imidazole and sodium chloride were added to the cellular supernatant prior to resin capture at final concentrations of 0.01M and 0.5M respectively. Clarified cell supernatants were then passed over Ni-NTA resin (Thermo Scientific), pre-equilibrated with NTA-A buffer (500mM NaCl, 10mM Tris, 0.02% azide, 10mM Imidazole, pH 7.1), washed with 10 column volumes of NTA-A buffer and then eluted in 1mL aliquots of NTA-B buffer (500mM NaCl, 10mM Tris, 0.02% azide, 500mM Imidazole, pH 8). Purified viral proteins were then concentrated and purified by size exclusion chromatography using a HiLoad 16/600 Superdex S200 (Milipore Sigma) column fitted to a BioRad NGC system. Final fractions containing the RSV proteins were concentrated, analyzed by SDS-Page and BLI, and flash frozen for future use.

ADI-19425, 1D3 and 2C1 IgGs were digested into Fabs using Endoproteinase Lys-C (New England BioLabs) by adding 1μg of Lys-C per 10mg of IgG and the mixture incubated overnight at 37°C. Following digestion, mixtures were incubated with Protein A resin for 1-2 h to remove any remaining Fc or undigested IgG. Resulting Fabs were further purified using an Enrich SEC 650 (Bio-Rad) column and concentrated.

Generation of a VH3-21/VL1-40 targeting bispecific ai-mAb

A recombinant VH3-21 and VL1-40 targeting bispecific ai-mAb with a "silent Fc" was developed using the platform developed by Moore et al.[39] Codon optimized cDNA encoding a signal peptide, the 1D3 variable heavy chain region and the human IgG1 constant domain harboring N208D, Q295E, N384D, Q418E, N421D, L368D, K370S, E233P, L234V, L235A, G236del, and S267K mutations (Chain A) was synthesized by Twist Biosciences and cloned into pTT3. A leader peptide followed by a 2C1 scFv (VH- $GKPGS_4$ -VL) fused to Human IgG1 residues 216 through 447 containing a C220S, E357Q, and S364K mutations (Chain B) was synthesized by Twist Biosciences and cloned into pTT3. pTT3 plasmids encoding Chain A, Chain B and the 1D3 light chain were co-transfected into 293-6E cells and purified using Protein A chromatography as described in "recombinant protein expression". Purified antibodies were then concentrated, buffer exchanged into 50 mM Tris pH 8.5, and then purified on a Hi-Trap Q HP (Cytiva) anion exchange column pre-equilibrated with 50 mM Tris, pH 8.5 and eluted over a gradient to 50% elution buffer (50 mM Tris, 1 M NaCl, pH 8.5) over 10 column volumes. Fractions containing the bispecific antibody were concentrated and then further purified by size exclusion chromatography on a HiLoad 16/600 Superdex S200 (Milipore Sigma) column. Final fractions containing the bispecific antibody were analyzed by SDS-PAGE, tested for binding specificity, concentrated, buffer exchanged into PBS, snap frozen and stored at −80°C until use.

B cell sorting

Bulk human B cell sorting

Cryopreserved PBMCs were thawed and resuspended in 200μL of EasySep buffer (1X PBS, 2% heat inactivated fetal bovine serum, 1μM EDTA). B cells were isolated using the Human B Cell Enrichment Kit (Stem Cell) according to the manufacturer's instructions. Enriched B cells were resuspended in 200μL EasySep buffer and incubated with 10μL rat serum, 10μL Mouse Serum, 10μL mouse-*anti*-human CD32 (BD), 10μL mouse-*anti*-human CD23 (BD) and 10μL mouse-*anti*-human CD16 (BD). Cells were then washed with EasySep buffer and resuspended in 200μL EasySep buffer containing CD19-BV711 (BioLegend) at a 1:200 dilution, CD27-PE-Cy7 (eBioscience) at a 1:600 dilution, CD14-FITC (BD Pharmingen) at a 1:60 dilution, CD3-FITC (BD Pharmingen) at a 1:60 dilution, CD20-AF700 (BioLegend) at a 1:300 dilution, IgD-PerCP-Cy5.5 (eBioscience) at a 1:120 dilution, IgM-BV605 (BioLegend) at a 1:120 dilution, Fixable Viability Dye, V500 (eBioscience) at a 1:300 dilution, and i) biotinylated murine anti-idiotypic mAbs conjugated to Total SeqC-0960 APC streptavidin (BioLegend), Total SeqC-0959 APC streptavidin (BioLegend), Total SeqC-0958 APC streptavidin (BioLegend), Total SeqC-0957 APC streptavidin (BioLegend), or Total SeqC-0956 APC streptavidin (BioLegend), ii) recombinant anti-idiotypic mAbs labeled with APC and PE (separately labeled pools) using Zenon Human IgG Labeling Kits (ThermoFisher Scientific) murine anti-idiotypic antibodies labeled with Zenon-PE. Cells were then washed with 5mL of EasySep buffer and suspended in 0.5 mL of EasySep buffer and subjected to analysis on a FACSymphony S6 (BD Biosciences). Naive B cells were defined as live (V500-), $CD14^{-}$, $CD3^{-}$, $CD19^{+}$, $CD20^{+}$, IgM^{+}, IgD^{+}, $CD27^{-}$. Naive ai-mAb^{+} (APC^{+}, PE^{+}) B cells were bulk sorted and BCR libraries were prepared using the Chromium Single Cell Human BCR Amplification Kit (10X Genomics).

Libraries from naive B cells that were positive for murine anti-idiotypic mAbs conjugated to TotalseqC streptavidin APC were generated using the Chromium Single Cell Human BCR Amplification Kit (10X Genomics) and the 5′ Feature Barcode Kit (10X Genomics). All single cell libraries were separately indexed using the Dual Index Kit TT Set A (10X Genomics) and sequenced on an Illumina NovaSeq or NextSeq instrument at the Fred Hutch Genomics Core. Analysis of flow data was processed using FlowJo 10.8.1 software (Tree Star).

Analysis of barcoded NGS sequences

Sequence files were obtained from the Fred Hutchison Genomics Core. FASTQ data was analyzed using Cell Ranger (Version 3.0.0) on an independent computing cluster. Subsequently, enclone[73] was used for clustering and visualization of B cell clonotypes in association with specific barcodes. Both enclone and Cell Ranger are programs developed and published by 10x Genomics used for downstream analysis of 10x compatible sequencing datasets (Pleasanton, CA). Briefly, Cell Ranger was run using sequencing data mentioned above in order to demultiplex using unique molecular identifier (UMI), index, and unique 10x compatible barcodes (total-SeqC Barcodes) present in the sequencing data. The output of Cell Ranger is demultiplexed data compatible with downstream use in enclone software. Enclone was then used to enumerate and visualize the grouping of VDJ sequences in association with respective 10x feature barcode. By extension, BCRs were assigned to the ai-mAB they were tagged with during sorting. Datasets produced in enclone were exported as CSV files and R studio was used to visualize data further.

Analysis was limited to B cells with a single heavy-light chain pair. The feature barcode counts for each antigen were analyzed using the *chngpt* package for R statistical software (R Foundation for Statistical Computing, Vienna, Austria) for a segmented generalized linear model[38] to determine a threshold for positivity (Figure S2). B cells above the threshold were considered positive for a given ai-mAb. We included the iv2 mAb labeled with a barcoded APC-barcode conjugate as a control for non-specific binding to murine IgG constant regions, or the SA-APC reagents. B cells that were above the threshold for the iv2 barcode were excluded from subsequent analysis.

Single B cell sorting and sequencing

In single B cell sorting experiments, B cells were thawed and enriched using magnetic beads from cryopreserved PBMCs as described above. Enriched B cells were resuspended in 200μL EasySep buffer and incubated with 10μL rat serum, 10μL mouse serum, 10μL mouse-*anti*-human CD32 (BD),10μL mouse-*anti*-human CD23 (BD) and 10μL mouse-*anti*-human CD16 (BD). Cells were then washed with EasySep buffer and resuspended in 200μL EasySep buffer containing CD19-BV711 (BioLegend) at a 1:200 dilution, CD27-PE-Cy7 (eBioscience) at a 1:200 dilution, $CD14^{-}$ PE (BD Pharmingen) at a 1:100 dilution, CD3-PE (BD Pharmingen) at a 1:100 dilution, CD20-AF700 (BioLegend) at a 1:250 dilution, IgD-PerCP-Cy5.5 (eBioscience) at a 1:100 dilution, IgM-BV605 (BioLegend) at a 1:100 dilution, and Fixable Viability Dye-V500 (eBioscience) at a 1:200 dilution. The bispecific antibody was conjugated to DyLight 488 NHS Ester (Thermo Scientific) according to the manufacturer's instructions, and used at a 1:5,000 dilution. After a 30-min incubation at 4°C, cells were washed with 5mLs of EasySep buffer, resuspended in 400μL PBS +2% BSA, and subjected to analysis on a FACSAria II (BD Biosciences). Naive B cells that stained positive for the bispecific antibody ($AF488^{+}$) were single-cell sorted into individual wells of 96 well plates and then were snap frozen on dry ice. Analysis of flow data was processed using FlowJo 10.8.1 software (Tree Star).

To sequence the VH/VL pairs from single sorted B cells, cDNA was generated using Superscript IV (Invitrogen) and the VH and VL sequences were recovered using gene-specific primers and cycling conditions previously described.[40] VH and VL amplicons were Sanger sequenced (Genewiz) and assigned antibody gene usage using IMGT/V-QUEST.[41]

Biolayer interferometry

BLI assays were performed on the Octet Red 96E Instrument (Sartorious) at 30°C with shaking at 1,000 RPM.

Bispecific ai-mAb-Fab binding screens

The purified bispecific antibody was captured using Anti-Human IgG capture (AHC, Sartorius) biosensors by immersing sensors into 250 μL kB buffer (1X PBS, 0.01% BSA, 0.02% Tween 20, and 0.005% NaN_3) with the bispecific ai-mAb at a concentration of 80nM for 200s. 1D3 IgG and 2C1 IgG were captured in the same fashion in parallel. After loading, the baseline signals were recorded for 60s in KB. The sensors were then immersed into wells containing ADI-19425 Fab, ADI-19425HC/AMMO1 LC chimeric Fab, iv8 HC/ADI-19425 LC chimeric Fab, or a negative control Fab at concentrations of 200nM for 300s (association phase). Following this, sensors were immersed in KB for an additional 300s (dissociation phase). The background signal obtained from sensors immersed in the negative control Fab was subtracted from each individual ai-mAb or the bispecific antibody.

Small scale mAb screens

Total antibody recovered from the small-scale production pipeline was assayed for binding to RSV-A preF (DS-Cav1) and postF by BLI.[15,43] Briefly, antibodies were captured by immersing AHC biosensors into 250μL of supernatant harvested from mAb-transfected 293E cultures on day 5 for 200s. After loading, baseline signals were recorded for 60s in KB. The sensors were then immersed in wells containing RSV-A preF or postF at concentrations of 140nM in 250ul KB for 300s. Following this, sensors were immersed in KB for an additional 300s. The binding screens included ADI-19425 and palivizumab as positive controls and VRC01[9,17,60] as a negative control.

Kinetic analysis of ai-mAbs and neutralizing VH3-21/VL1-40 mAbs

AHC sensors were immersed in KB containing 67nM of 1D3 IgG or 2C1 IgG for 300s. After loading, the baseline signal was recorded for 60s in KB. Next, sensors were immersed in wells containing ADI-19425 Fab at serial dilutions ranging from 250nM to 0nM for 300s (association), followed by dissociation in KB for an additional 300s. As a negative control, wells containing no mAb were used, and the background signal from these wells were subtracted from the mAb-containing wells. Curve fitting was performed using a 1:1 binding model and the ForteBio data analysis software. Mean K_a and K_D values were determined by averaging all binding curves that matched the theoretical fit with an R^2 value of >0.99. The average K_D for each ai-mAb was determined by dividing the average K_D by the average K_a.

Steady state kinetic analysis of sorted VH3-21/VL1-40 mAbs

Purified VH3-21/VL1-40 mAbs recovered from sorting experiments that showed measurable binding to RSV-A preF, were further analyzed for steady state binding kinetics by BLI. VH3-21/VL1-40 mAbs were immobilized on AHC sensors at a concentration of 67nM in KB for 300s. Following loading, baseline signals were recorded for 60s in KB. As the association step, sensors were immersed in wells containing serial dilutions of RSV-A preF or postF from 250 nM to 0nM in KB for 300s, followed by immersion into wells containing KB to measure dissociation for 300s. Double subtractions of parallel wells containing an irrelevant EBV mAb (E1D1)[74] as well as non-mAb loaded sensors associated in RSV-A preF were applied to each sample. Curve fitting was performed using a 1:1 binding model in the ForteBio data analysis software. Steady state Response values (average R_{max} values for final 5s of association step) were plotted for each mAb concentration in the dilution series. All experiments were performed twice, and the mean Response was plotted. Additionally, the average dissociation rates (K_D) measured over the whole concentration series under these conditions were plotted for each mAb.

For RSV-neutralizing VH3-21/VL1-40 mAbs (MLR_15, MLR_24, MLR_55), steady state binding kinetics were also calculated for RSV-B preF. The assay described above was repeated using RSV-B preF, after which curve fitting was performed using a 1:1 binding model and the ForteBio data analysis software. Steady state Response values (average R_{max} values for final 5s of association step) were plotted for each mAb concentration in the dilution series. In addition, average apparent affinity (bivalent K_D) was calculated for these mAbs as described above for ai-mAbs to ADI-19425 Fab.

Competition assays

VH3-21/VL1-40 mAbs were biotinylated using the EZ-Link NHS-PEG4-Biotin kit (ThermoFisher Scientific) at a theoretical 1:1 ratio of mAb to biotin. Biotinylated mAbs were immobilized on streptavidin biosensors (SA, Sartorious) at 100nM in KB buffer for 300s, after which a baseline signal was read for 60s in KB. Sensors were then immersed for 300s in wells containing 25nM of RSV-A preF that had been pre-incubated with 250nM of the previously characterized RSV mAb ADI-14337(site III) or VRC01 (control mAb) in KB for 1 h at 37°C.[9,60] Following this, the dissociation rate was measured in KB for 300s. Assays were repeated twice, and mean values shown. The following equation was used to calculate the % of Max Binding in the presence of competing mAbs:

$$\text{\% of Max Binding} = (R_{max}\ \text{preF} + \text{ADI} - 14337 \,/\, R_{max}\ \text{preF} + \text{VRC01}) \times 100$$

Generation of B cell lines

BCR expression constructs were designed as previously described.[49,75] cDNA consisting of a signal peptide-rearranged VDJ-IgG constant regions (membrane anchored splice variant)-furin cleavage site-T2A self-cleaving peptide-signal peptide-rearranged VK or VL and kappa or lambda constant regions were codon-optimized and synthesized, and cloned into pTwist Lenti SFFV Puro WPRE to create pTwist Lenti SFFV Puro WPRE-ADI-19425-BCR, pTwist Lenti SFFV Puro WPRE-ADI-19425HC/AMMO1LC-BCR, pTwist Lenti SFFV Puro WPRE-iv8HC/ADI-19425LC-BCR, pTwist Lenti SFFV Puro WPRE-ADI-19470-BCR, pTwist Lenti SFFV Puro WPRE-ADI-24811-BCR and pTwist Lenti SFFV Puro WPRE-ADI-25542-BCR. pTwist Lenti-based BCR expression constructs were co-transfected with psPAX2 (a gift from Didier Trono, Addgene) and pMD2.G (a gift from Didier Trono, Addgene) at a 4:2:1 ratio into suspension adapted 293T cells in Freestyle 293 media (ThermoFisher Scientific) using 293-Free Transfection reagent (Millipore

Sigma) according to the manufacturer's instructions. 4 days later the culture was centrifuged at 300 × *g* to remove cells and debris and the 1mL of viral supernatant was added to 4mLs of DG-75 (human male) cells at 1×10^6 cells/mL in cRPMI plus 2 μg/mL polybrene. 24–48 h post-transduction and indefinitely thereafter, puromycin was added to the cell cultures at a concentration of 1 μg/mL. Generation of the DG-75 cell line transduced with the gl12A21 BCR was previously described.[35]

Ai-mAb staining of BCR-transduced cell lines

Surface expression of BCR-transduced cell lines were confirmed by staining with fluorescently labeled ai-mAbs and subsequent analysis via flow cytometry. Briefly, parental ai-mAbs 1D3 and 2C1 were labeled with PE and APC, respectively, using the Zenon Human IgG Labeling Kits (ThermoFisher Scientific). These labeled antibodies in addition to the DyLight-488 labeled bispecific ai-mAb were used to surface stain BCR-transduced cell lines. BCR expression was assessed on a FACSymphony A5 (BD Biosciences) flow cytometer and analyzed using FlowJo 10.8.1 software (Tree Star).

B cell activation in BCR-transduced cell lines

Calcium mobilization in B cells stably expressing BCRs upon bispecific antibody stimulation was monitored as previously described.[49,50] Briefly, cells were loaded with Fluo-4 Direct calcium indicator (Invitrogen) and mixed 1:1 with complete RPMI at 37°C for 1 h. Cells were pelleted and stained with PE-conjugated anti-human Fcγ Fab (Jackson ImmunoResearch) (1:100 dilution in 100 μL complete RPMI plus Fluo-4 Direct) for 30 min. The cells were washed with 5 mL of complete RPMI and resuspended at $\sim2\times10^6$ cells/mL in complete RPMI and subjected to Ca^{2+} flux analysis at a medium flow rate on a FACSymphony A5 (BD Biosciences) flow cytometer. In all cases BCR transduced cells were mixed with untransduced cells as an internal control at a 5:2 ratio.

Levels of background fluorescence (Min_{FL}) were determined by averaging the background Fluo-4 absorbance in cells for 30s. After that, activation of B cells expressing exogenous BCRs by the various immunogens was determined by monitoring changes in Fluo-4 fluorescence associated with cells expressing the exogenous BCRs (PE^+ cells) for 210s. The bispecific ai-mAb, parental ai-mAbs (1D3, 2C1), and control mAb (VRC01), were added at a final concentration of 100 nM. RSV-A preF was added to final concentration of 1μM. An α-IgG Fcγ $F(ab')_2$ (Jackson ImmunoResearch) was added to a final concentration of 100nM as a positive control. Ionomycin was added to a final concentration of 150 nM for 60s following the immunogen addition, and maximum Fluo-4 fluorescence (Max_{FL}) was established by averaging the Fluo-4 fluorescence signal recorded during the last 10s. The percent of maximum Fluo-4 fluorescence at each time point *t* was determined using the formula: (Fluorescence at t-Min_{FL})/(Max_{FL}-Min_{FL}) × 100. This analysis was performed on both the BCR positive (anti-Human Fcγ-PE^+) and BCR negative cells (anti-Human Fcγ-PE^-) simultaneously. The background Fluo-4 fluorescence signal from the BCR negative cells was subtracted from that of the BCR positive population at each time point. All flow analysis was done with FlowJo 10.8.1 software (Tree Star).

Fab-Fab complex formation

ADI-19425 and 1D3 Fabs were mixed at a 1.5:1 (ADI-19425:1D3) molar ratio and kept at 4°C rotating overnight. The resulting complex was purified using HiLoad 16/600 Superdex S200 (Milipore Sigma) column, resulting in a major peak consisting of the Fab-Fab complex and a minor peak consisting of the excess ADI-19425 Fab. Fractions from the major peak were pooled and concentrated to 10 mg/mL. ADI-19425 and 2C1 Fabs were mixed at a 1.2:1 (ADI-19425:2C1) molar ratio and complexed and purified the same way as the ADI-19425 – 1D3 complex. The ADI-19425 – 2C1 complex was concentrated to 9.8 mg/mL.

Crystallization and data collection

For both complexes, crystallization conditions were screened at RT using the sitting drop vapor diffusion method with a Formulatrix NT8 drop setting and Rock Imager.

ADI-19425 Fab – 1D3 Fab complex screening was done with Clear Strategy I and II, MCSG1-3, ProPlex (Molecular Dimensions), WPS1 and 2 (Rigaku), Xtal HT, and an Additive screen (Hampton Research). Crystals from the MCSG2 B1 condition with Additive Screen H5 (0.1M MES pH 6.5, 12% w/v PEG 20K, 4% formamide) were further optimized using the hanging drop method at RT. Final crystals were grown in a solution of 0.1M MES pH 6.5, 12% PEG 20K, and 4% formamide. A cryoprotectant of 0.1M MES pH 6.5, 12% PEG 20K, 4% formamide and 40% ethylene glycol was used. Crystals diffracted to 2.67 Å. Data was collected at ALS beamline 5.0.2 and processed using XDS.[76] ADI-19425 Fab – 2C1 Fab complex screening was done with MCSG1-3, Morpheus, ProPlex (Molecular Dimensions), WPS1 (Rigaku) and Xtal HT (Hampton Research). Crystals from the WPS1 H1 condition (0.1M Imidazole pH 6.5, 10% PEG 3350, 30% MPD and 0.2M $(NH_4)_2SO_4$ were further optimized using the hanging drop method at RT. Final crystals were grown in a solution of 0.1M Imidazole pH 6.5, 12% PEG 3350, 30% MPD, and 0.2M $(NH_4)_2SO_4$. A cryoprotectant of 0.1M Imidazole pH 6.5, 12% PEG 3350, 30% MPD, and 30% ethylene glycol was used. Crystals diffracted to 2.41 Å. Data was collected at Advanced Photon Source beamline 19-ID and processed using HKL3000.[62] The data collection statistics are summarized in Table S1.

Structure solution, refinement, and analysis

The structures of both ADI-19425 Fab – 1D3 Fab and ADI-19425 Fab – 2C1 were solved by molecular replacement with Phaser in Phenix.[64] Chain H (heavy chain) of PDB ID: 1D5I [77] and Chain A (light chain) of PDB ID: 5I76 [78] were used as the search model for

CellPress
OPEN ACCESS

1D3 Fab. The anti-IL-7Ralpha 2B8 Fab of PDB ID: 6P67 [79] was used as the search model for 2C1 Fab. An existing ADI-19425 structure (PDB ID: 6APC [9]) was used as the search model for ADI-19425 Fab. All search models were divided into VL:VH and CL:CH1 components. Model building was done using Coot[65,66] and refinement was performed in Phenix.[64] The ADI-19425 Fab – 1D3 Fab structure had eight Fab-Fab complexes in the unit cell, but electron density was missing for four ADI-19425 CL:CH1 domains, which were not placed in the structure. The ADI-19425 Fab – 2C1 Fab structure had one Fab-Fab complex in the unit cell. The data collection and refinement statistics are summarized in Table S1. Structural figures were made in PyMOL[80] and Inkscape.[81] BSA analysis was done using PISA.[67,68] For the ADI-19425 Fab – 1D3 Fab complex structure, chains E, F, G and H were used for BSA analysis.

Negative stain electron microscopy and analysis

Purified DS-Cav1 was diluted to 0.03 mg/mL in Tris buffer saline (10mM Tris-HCl, 150mM NaCl, pH 7.5). 4μL of this sample was applied to formvar/carbon Ted Pella 400 mesh copper grids and stained with 2% (w/v) uranyl formate. Grids were imaged at 120kV on a Talos L120C Transmission Electron Microscope. Data was collected using the Leginon software[69] at 1.58 Å pixel size and 2mm spherical aberration with a 40 e/Å^2 total exposure dose. Micrographs were loaded into Cryosparc[70] and processed. Particles were picked using Blob Picker, and unbound particles and noise were filtered out until 10,059 particles remained.

Purified DS-Cav1 was spiked with 3.2 M excess Fab per DS-Cav1 trimer and grids were immediately stained and later imaged as described for the unbound DS-Cav1 protein. Micrographs were loaded into Cryosparc and processed. Particles were picked using Blob Picker, and unbound particles and noise were filtered out. 14,158 total particles were run through Ab-Initio and Homogeneous Refinement before a 3D class was generated. The 3D model was analyzed in ChimeraX.[71]

Virus production

Recombinant RSV subtype strain A2-GFP and subtype strain B1-GFP were obtained from ViraTree. Viruses were cultured and viral titers determined as previously described.[44] Briefly, RSV viruses were cultured on HEp-2 cells (human male) in cDMEM and harvested by physical disruption. Viral supernatant was purified from cell pellets by centrifugation at 4,000 × *g*, followed by decanting. Viral supernatant was aliquoted in 1mL vials, flash frozen, and stored at −80°C. Viral titers were determined through infection of Vero cells (African green monkey) in 24-well plates over serial dilutions of the virus, overlaying with DMEM including 0.8% methylcellulose (Sigma-Aldrich). Fluorescent scans were completed using a Typhoon imager (GE Life Sciences) at 5 days post-infection, and plaques were quantified using ImageJ (LOCI, University of Wisconsin).

Plaque reduction neutralization assays

As a primary screen of neutralization activity of VH3-21/VL1-40 mAbs, plaque reduction assays were run on Vero cells at a set mAb concentration of 500 μg/mL. Vero cells were seeded at 100,000 cells/mL in 24 well plates and cultured for 48–72 h. VH3-21/VL1-40 mAbs were diluted to 500 μg/mL in DMEM and then mixed with an equal volume of RSV-A-GFP or RSV-B-GFP diluted to 2,000 pfu/mL and incubated for 1 h at 37°C. Vero cells were then incubated with 100μL of the antibody/virus mixture for 1 h at 37°C, followed by addition of 500μL DMEM containing 0.8% methylcellulose. Fluorescent images were taken at 5 days post-infection using a Typhoon imager, and plaques were counted using ImageJ. Plaque counts were transformed into % infectivity by dividing the well counts by those from no antibody control wells and multiplying by 100%. Antibodies that had visible neutralization capacity, reduced % infectivity vs. no antibody containing wells, were followed up for IC_{50} titer calculation. All antibodies were run in triplicate over a single experiment for these initial screens.

mAb IC_{50} and IC_{80} values were further determined by plaque reduction assays. Vero cells were prepared as above, and monoclonal antibodies were serially diluted in 80μL of DMEM mixed with 80μL of RSV-A-GFP or RSV-B-GFP diluted to 2,000 pfu/mL and incubated for 1 h at 37°C. Vero cells were then incubated with 100μL of the antibody/virus mixture for 1 h at 37°C, followed by addition of 500μL DMEM containing 0.8% methylcellulose. Fluorescent images were taken at 5 days post-infection using a Typhoon imager, and plaques were counted using ImageJ. Plaque counts were transformed into % infectivity by dividing the well counts by those from the highest antibody dilution for each respective antibody multiplied by 100%. % infectivity was plotted as a function of antibody dilution. The neutralization curves were fit using the log(inhibitor) versus response - variable slope (four parameters) analysis in Prism 9.4.0. The half maximal inhibitory antibody dilution IC_{50} and IC_{80} were interpolated from the curve in Prism 9.5.0. Undetermined values were reported as 2x the highest concentration tested. Within each assay antibodies were run in duplicate, and assays were repeated 3–4 times.

Plaque reduction neutralization competition assays

IC_{50} and IC_{80} concentrations for MLR_24 against RSV-A and RSV-B were determined as above. MLR_24 at its IC_{50} and IC_{80} concentrations against RSV-A and RSV-B were prepared in 80μL of DMEM mixed with 80μL of RSV-A-GFP or RSV-B-GFP diluted to 2,000 pfu/mL in triplicate wells and incubated for 1 h at 37°C. In parallel, MLR_24 was mixed at its IC_{50} and IC_{80} concentrations against RSV-A and RSV-B with matched amounts of 5 preF-binding, non-neutralizing mAbs (MLR_27, MLR_30, MLR_33, MLR_42, MLR_45) in 80μL of DMEM mixed with 80μL of RSV-A-GFP or RSV-B-GFP diluted to 2,000 pfu/mL in triplicate wells and incubated for 1 h at 37°C. Vero cells were then incubated with 100μL of the antibody/virus mixture for 1 h at 37°C, followed by addition of 500μL DMEM containing 0.8% methylcellulose. Fluorescent images were taken at 5 days post-infection using a Typhoon imager, and

plaques were counted using ImageJ. Plaque counts were transformed into % infectivity by dividing the well counts by the average of those from infection of cells without antibody multiplied by 100%.

QUANTIFICATION AND STATISTICAL ANALYSIS

Data were analyzed, and graphs created using GraphPad Prism 9.5.0 software. Statistical tests used are included in figure legends where applicable as well as sample sizes and replicates.

Cell Reports, Volume *43*

Supplemental information

Targeting RSV-neutralizing B cell receptors

with anti-idiotypic antibodies

Samuel C. Scharffenberger, Yu-Hsin Wan, Leah J. Homad, Gargi Kher, Austin M. Haynes, Bibhav Poudel, Irika R. Sinha, Nicholas Aldridge, Ayana Pai, Madeleine Bibby, Crystal B. Chhan, Amelia R. Davis, Zoe Moodie, Maria Belen Palacio, Amelia Escolano, M. Juliana McElrath, Jim Boonyaratanakornkit, Marie Pancera, and Andrew T. McGuire

A

```
                         10        20        30        40        50        60        70        80        90       100       110       120  Accession
IGHV3-21*01   EVQLVESGGGLVKPGGSLRLSCAASGFTFSSYSMNWVRQAPGKGLEWVSSISSSSSYIYYADSVKGRFTISRDNAKNSLYLQMNSLRAEDTAVYYCAR------------------------     Number
ADI-19425 HC  ..................................................................................................LGYCSGGSCH-FDYWGQGTLVTVSS  MG524063
ADI-14337 HC  ..................................................................................................EYYDSSGYTNWFDPWGQGTLVTVSS  MG524251
ADI-25532 HC  Q....Q............................................................................................---GSSSSWYYFDYWGQGTLVTVSS  MG524182
```

B

```
                         10        20        30        40        50        60        70        80        90       100       110  Accession
IGLV1-40*01   QSVLTQPPSVSGAPGQRVTISCTGSSSNIGAGYDVHWYQQLPGTAPKLLIYGNSNRPSGVPDRFSGSKSGTSASLAITGLQAEDEADYYCQSYDSSLSG-------------    Number
ADI-19425 LC  .P.................................................................................................FYVFGTGTKLTVL  MG524528
ADI-14337 LC  .P................................................................................................A--WVFGGGTQLTVL  MG524647
ADI-25532 LC  ...V...............................................................................................--YVFGTGTKVTVL  MG524716
```

Figure S1: Alignment of unmutated VH3-21/VL1-40 mAbs used to generate anti-idiotypic hybridomas. Related to Figure 1. (**A**) Alignment of ADI-19425, ADI-14337, and ADI-25532[S1] heavy chains to germline VH3-21*01. (**B**) Alignment of ADI-19425, ADI-14337, and ADI-25532 light chains to germline VL1-40*01.

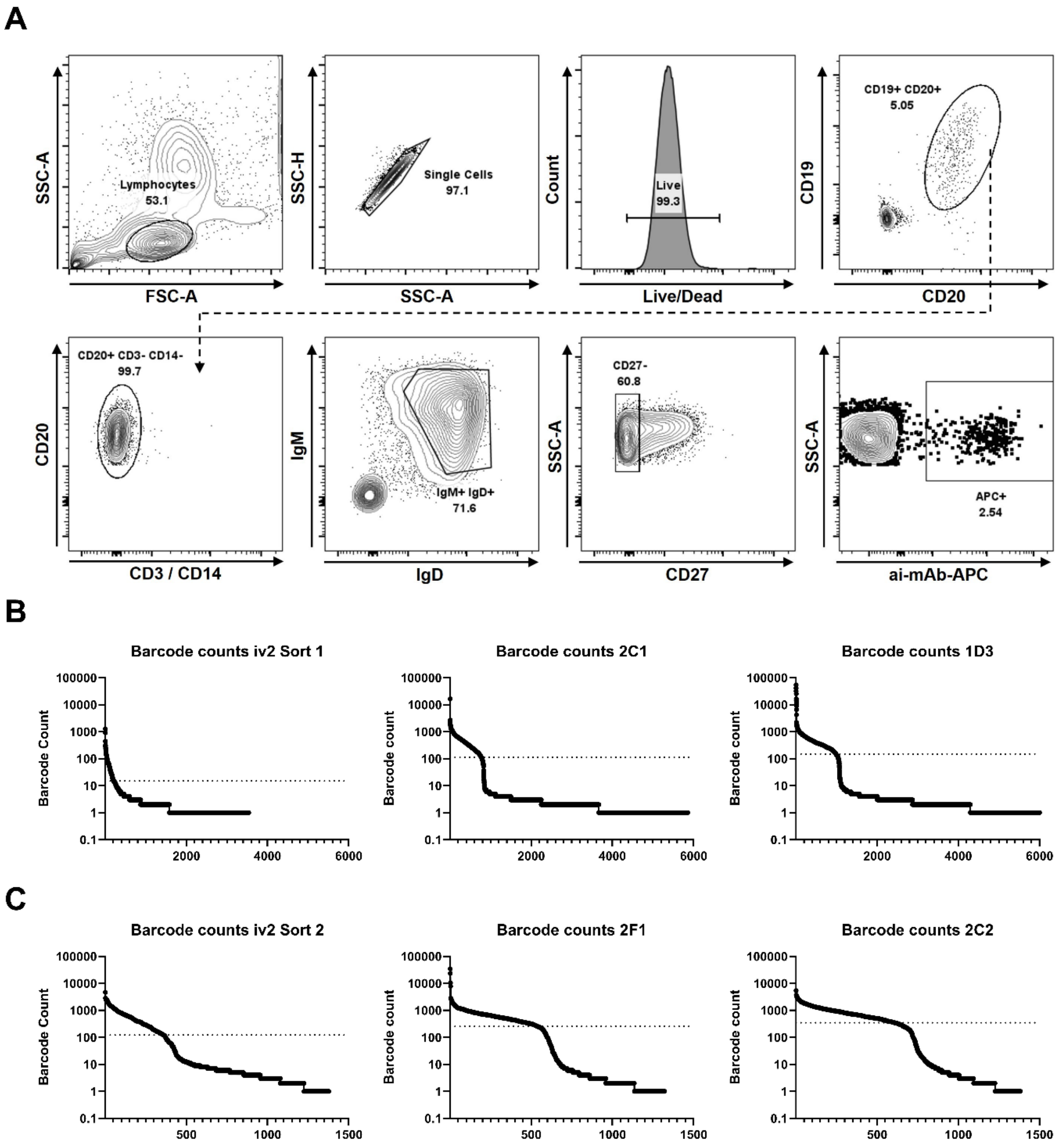

Figure S2: Gating strategy to sort ai-mAb specific naïve B cells and barcode threshold setting. Related to Figure 1. (**A**) Gating as follows: Single cells → Lymphocytes → Live cells → $CD3^-$ $CD14^-$ → $CD19^+$ $CD20^+$ → IgD^+ IgM^+ → ai-mAb-APC^+ B cells. Cell percentages of parent gate are shown, representative staining plots from 1 group of sorted cells. (**B & C**) ai-mAbs were labeled with APC conjugated to a unique oligonucleotide feature barcode and used to stain naïve PBMC in two cocktails containing iv2, 2C1, 1D3 (**B**) and iv2, 2F1, and 2C2 (**C**). Individual B cell libraries were generated using the chromium platform from 10X Genomics. Each panel shows the number of times the indicated feature barcode was observed for a given cell plotted from highest to lowest where each dot represents a single B cell. The thresholds, represented by the horizontal dotted line in each panel, were estimated by segmented generalized linear models using the *chngpt* R package[S2]. BCR sequences from B cells above

the threshold were considered positive for a given ai-mAb. B cells above the threshold from the iv2 isotype control in each sort were discarded from subsequent analysis.

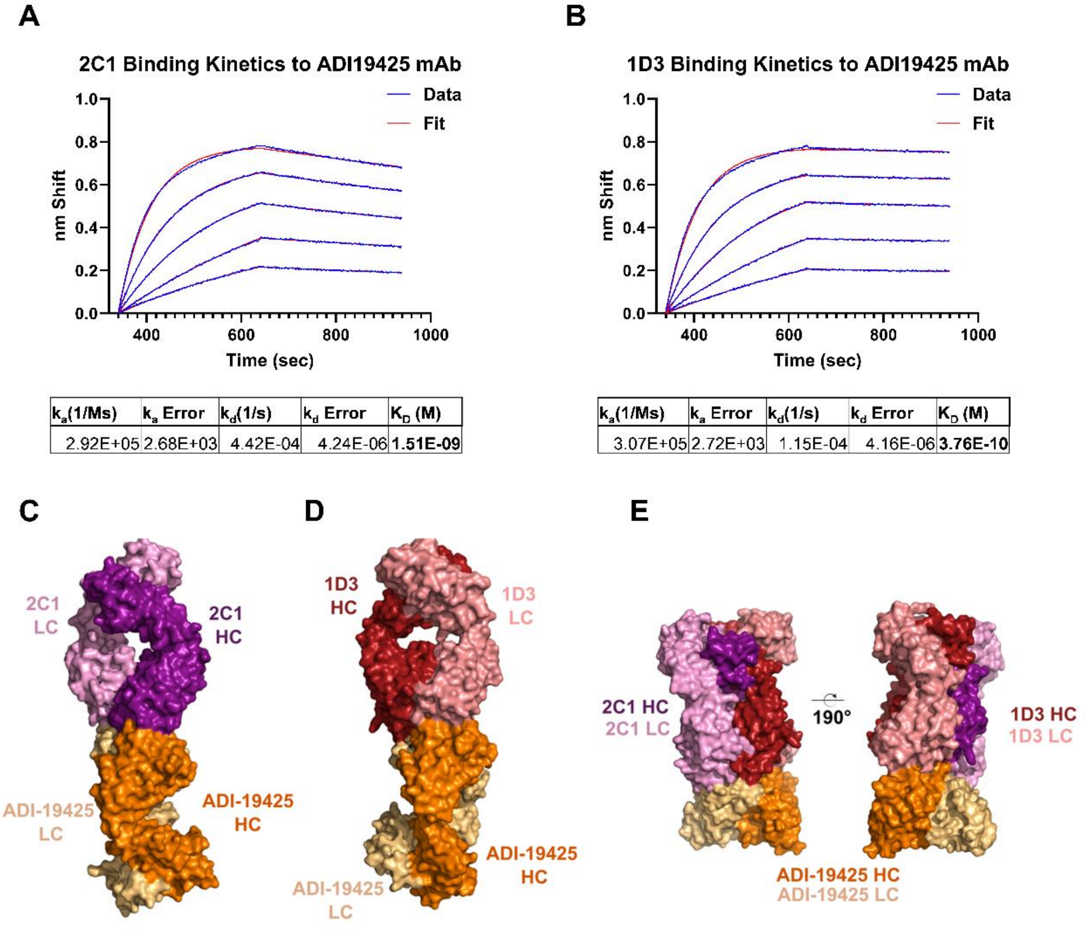

A

k_a(1/Ms)	k_a Error	k_d(1/s)	k_d Error	K_D (M)
2.92E+05	2.68E+03	4.42E-04	4.24E-06	**1.51E-09**

B

k_a(1/Ms)	k_a Error	k_d(1/s)	k_d Error	K_D (M)
3.07E+05	2.72E+03	1.15E-04	4.16E-06	**3.76E-10**

Figure S3: Binding kinetics of ai-mAbs to ADI-19425 Fab and Fab-Fab structures. Related to Figure 2. (**A** & **B**) Binding kinetics of recombinant 2C1 (**A**) or 1D3 (**B**) IgG to ADI-19425 Fab at the indicated concentrations were measured by biolayer interferometry. Blue lines represent observed data, and red lines are theoretical fit. The calculated kinetic parameters are shown below each plot. Data shown from a single biological replicate. (**C-E**) Full view of complexes between (**C**) 2C1 Fab (dark purple – heavy chain, light purple – light chain) and (**D**) 1D3 Fab (red – heavy chain, pink – light chain) with ADI-19425 Fab (orange – heavy chain, wheat – light chain). (**E**) Overlays of both complexes are shown aligned by the VH/VL region of ADI-19425 Fab and rotated on the y-axis by 190°.

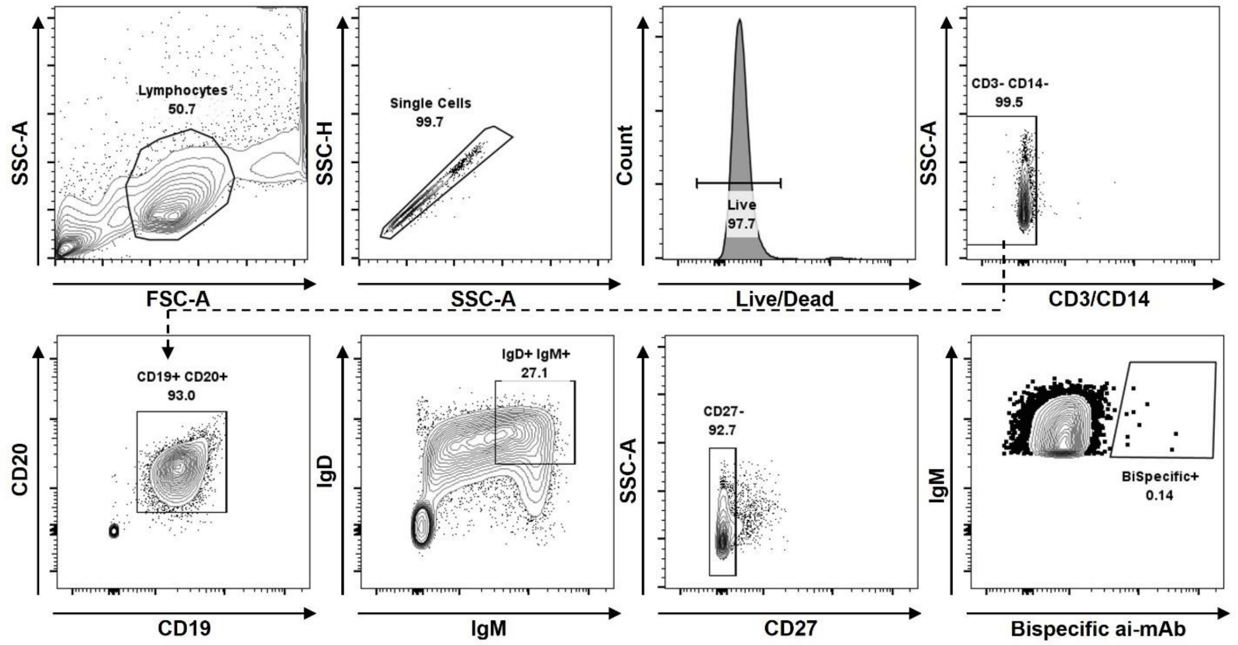

Figure S4: Gating strategy to identify and single cell sort ai-mAb specific naïve B cells. Related to Figure 4. Gating as follows: Lymphocytes → Single cells → Live cells → $CD3^-$, $CD14^-$ → $CD19^+$ $CD20^+$ → IgD^+ IgM^+ → $CD27^-$ → IgM^+ Bispecific ai-mAb$^+$. Percentages of parent gate indicated, representative gating from one sorting experiment.

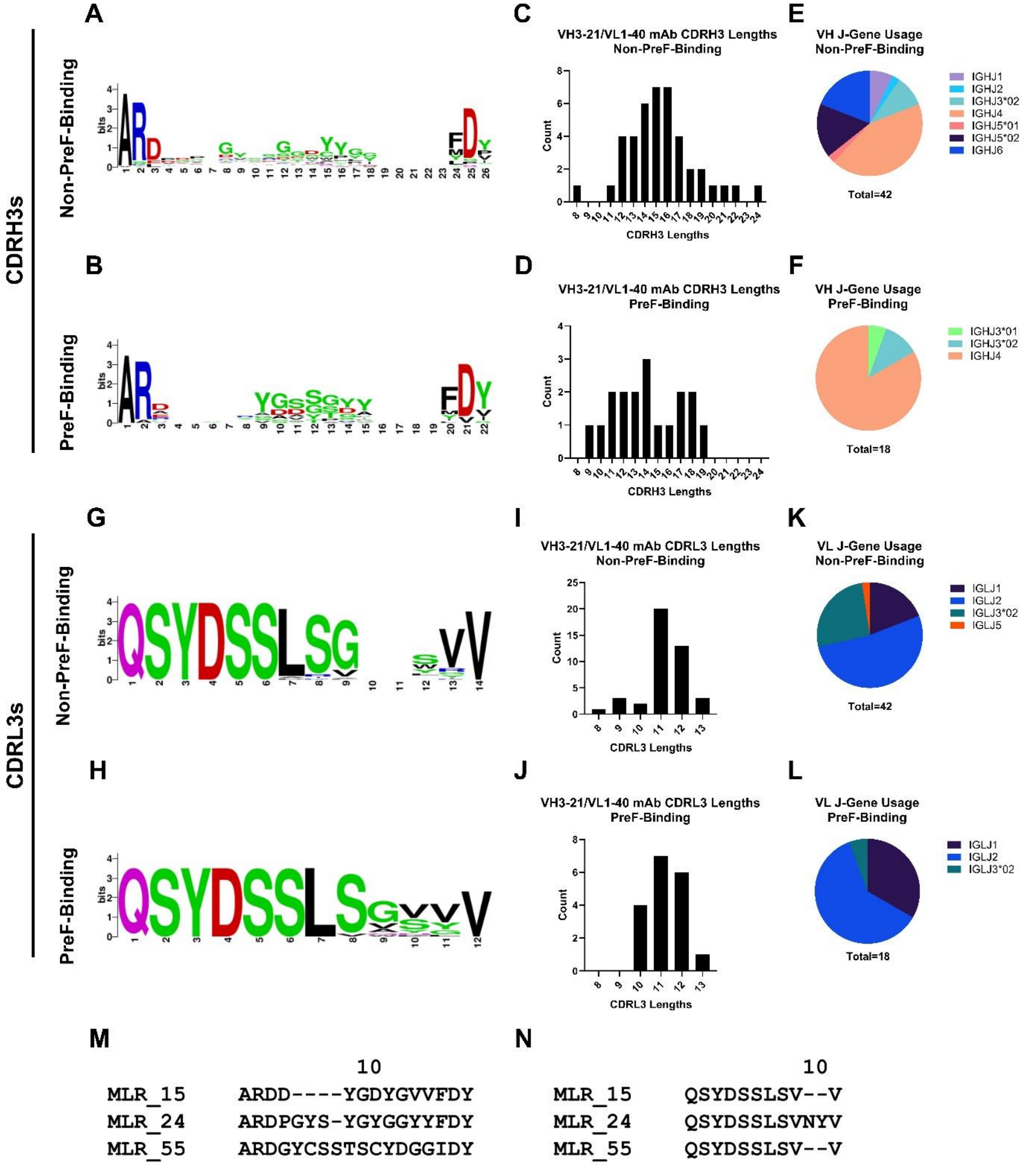

Figure S5: CDR3 and J-gene analysis of ai-mAb-sorted VH3-21/VL1-40 mAbs. Related to Figure 4. (**A** & **B**) Sequence logo plots of non-preF-binding **A** and preF-binding **B** CDRH3s of sorted VH3-21/VL1-40 mAbs created with https://weblogo.berkeley.edu/logo.cgi. (**C** & **D**) Histograms of CDRH3 lengths of non-preF-binding **C** and preF-binding **D** VH3-21/VL1-40 mAbs. (**E** & **F**) Heavy chain J-gene usage of non-preF-binding **E** and preF-binding **F** VH3-21/VL1-40 mAbs. J-gene alleles with the same amino acid usage combined for analysis. (**G** & **H**) Sequence logo plots of non-preF-binding **G** and preF-binding **H** CDRL3s of sorted VH3-21/VL1-40 mAbs. (**I** & **J**) Histograms of CDRL3 lengths of non-preF-binding **I** and preF-binding **J** VH3-21/VL1-40 mAbs. J-gene alleles with the same amino acid usage combined for analysis (**K**

& **L**) Light chain J-gene usage of non-preF-binding **K** and preF-binding **L** VH3-21/VL1-40 B cells. (**M** & **N**) Alignments of CDRH3s **M** and CDRL3s **N** of RSV-neutralizing VH3-21/VL1-40 B cells.

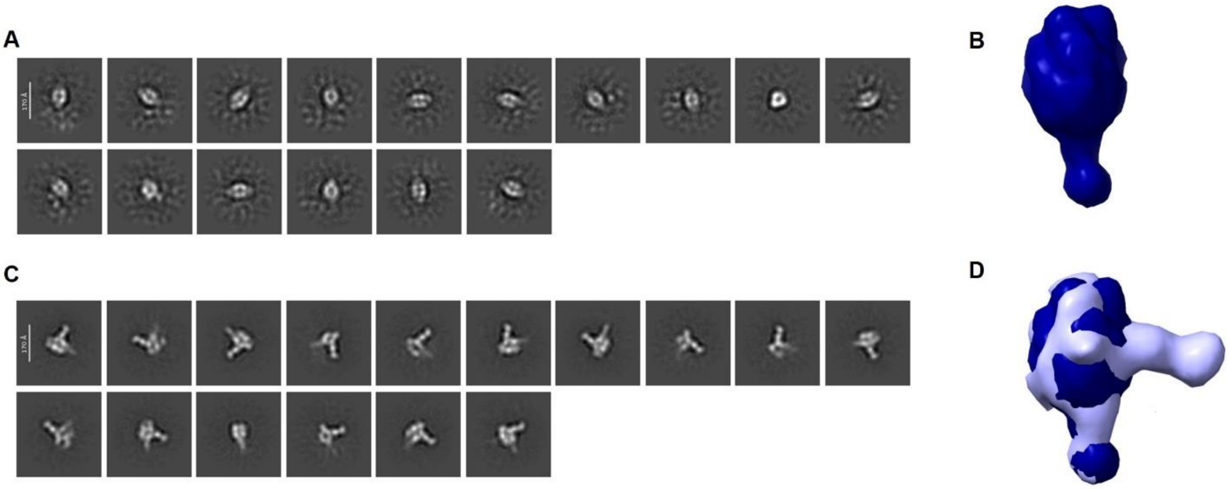

Figure S6: nsEM Images of DS-Cav1 and MLR_24 Fab – DS-Cav1 Complex. Related to Figure 4. (**A**) Representative 2D classes of unbound DS-Cav1[S3]. (**B**) A 20Å map of a crystal structure of RSV preF with a trimerization domain (PDB ID: 5TDG). (**C**) Representative 2D classes of the DS-Cav1 – MLR_24 Fab complex. (**D**) The 20Å map of the RSV preF crystal structure (dark blue) superimposed onto a representative 3D class of the DS-Cav1 – MLR_24 Fab complex (light purple).

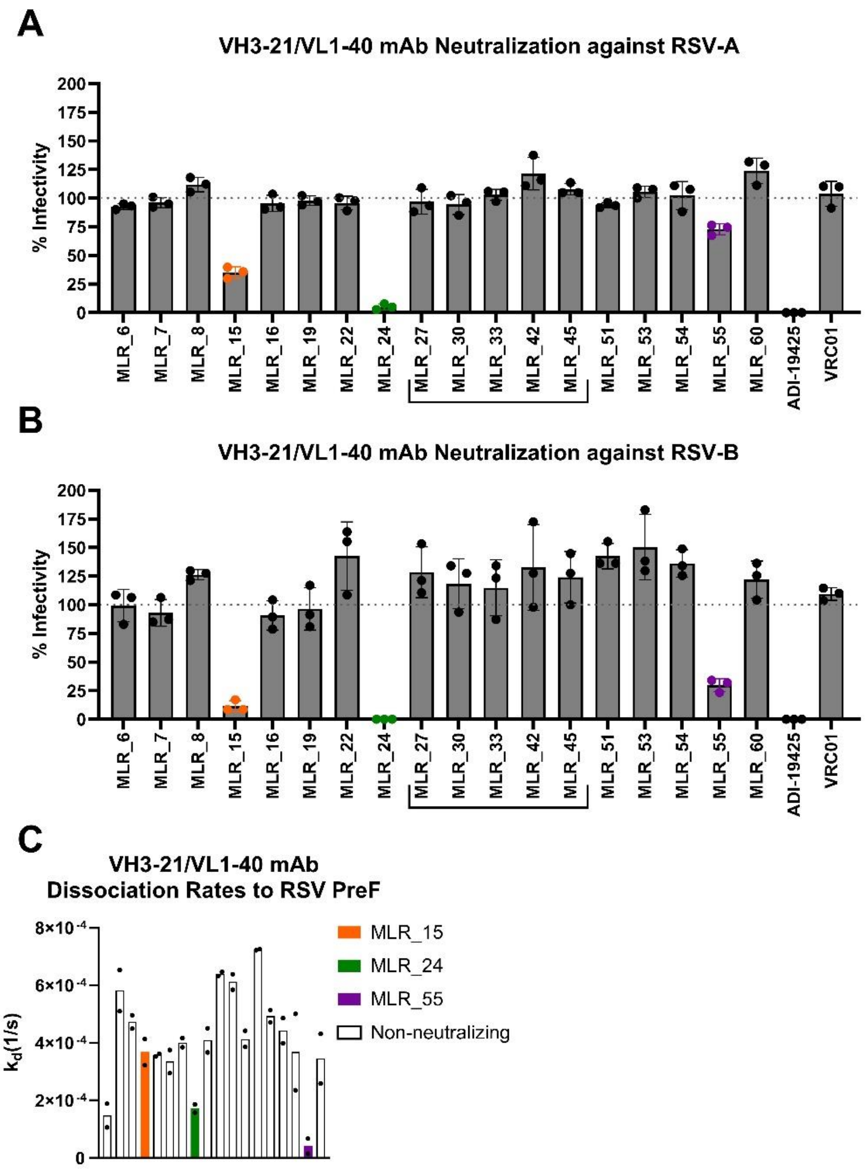

Figure S7: Neutralization screen of VH3-21/VL1-40 sorted mAbs against RSV-A / B and Steady State Affinity Measurements. Related to Figures 5 and 7**.** All recombinant VH3-21/VL1-40 preF-binding mAbs sorted with the bispecific ai-mAb were screened for neutralization activity against RSV-A (**A**) or RSV-B (**B**) at 500 µg/mL final dilution using a plaque reduction assay in triplicate. % Infectivity is the infectivity in the presence of mAb ÷ infectivity in the absence of mAb × 100%. Colored mAbs indicate those followed up for IC_{50} determination. VRC01[S4] is an irrelevant HIV mAb and included as a negative control. Bracketed mAbs were used in neutralization competition assays in **Fig. 7A & B**. Each point represents a technical replicate from a single experiment. Bars represent mean ± standard deviation. (**C**) Dissociation rates for VH3-21/VL1-40 mAbs averaged over the dilution series in **Fig. 5C**, coloring consistent as above. Each data point is is a biological replicate, bars indicate mean.

	ADI-19425 Fab - 1D3 Fab	ADI-19425 Fab - 2C1 Fab
**Data collection**		
Space group	P1	$P12_11$
Cell dimensions		
a, *b*, *c* (Å)	95.07, 149.98, 164.43	66.80, 66.93, 130.91
α, β, γ (°)	83.98, 89.90, 74.61	90, 103.74, 90
Resolution (Å)	49.45-2.72 (2.72 – 2.67)	50.00–2.44 (2.44-2.40)
R_{merge} [a]	0.116 (0.566)	0.242 (0.891)
<I/σ(I)>	4.0 (0.7)	15.4 (2.1)
$CC_{1/2}$	0.961 (0.319)	0.953 (0.844)
Completeness	92.5 (92.4)	98.4 (92.5)
Redundancy	1.9 (1.9)	6.6 (6.4)
**Refinement**		
Resolution (Å)	49.33 – 2.67 (2.77 – 2.67)	42.16 – 2.41 (2.50 – 2.41)
No. unique reflections	228772 (22653)	42798 (3991)
R_{work} [b] / R_{free} [c]	23.1/27.3 (32.6/37.2)	20.5/25.3 (26.9/33.1)
No. atoms	45020	6660
Protein	44852	6464
Water	178	166
Ligand	0	60
B-factors (Å^2)	56.24	34.79
Protein	56.31	34.80
Water	39.65	33.19
Ligand	0	41.86
RMS bond length (Å)	0.003	0.005
**Ramachadran Plot Statistics**[d]		
Residues	5948	861
Most Favored region	94.16	95.28
Allowed Region	5.48	4.37
Disallowed Region	0.36	0.35
Clashscore	6.10	6.66
**PDB ID**	8VS8	8VS7

[a] $R_{merge} = [\sum_h \sum_i |I_h - I_{hi}| / \sum_h \sum_i I_{hi}]$ where I_h is the mean of I_{hi} observations of reflection *h*. Numbers in parenthesis represent highest resolution shell. [b] R_{factor} and [c] $R_{free} = \sum||F_{obs}| - |F_{calc}|| / \sum|F_{obs}|$ x 100 for 95% of recorded data (R_{factor}) or 5% data (R_{free}). [d] Determined using MolProbity (10.1002/pro.3330)

Table S1: Data collection and refinement statistics for crystal structures

Name	Donor	RSV PreF Rmax (DS-Cav1)*	RSV-A Neut. IC_{50} (μg/mL)**	RSV-B Neut. IC_{50} (μg/mL)**	GenBank Accession Number (VH)	GenBank Accession Number (VL)
MLR_1	A	N.B.	N.N.	N.N.	PP429379	PP429439
MLR_2	A	N.B.	N.N.	N.N.	PP429380	PP429440
MLR_3	A	N.B.	N.N.	N.N.	PP429381	PP429441
MLR_4	A	N.B.	N.N.	N.N.	PP429382	PP429442
MLR_5	A	N.B.	N.N.	N.N.	PP429383	PP429443
MLR_6	A	0.311	N.N.	N.N.	PP429384	PP429444
MLR_7	A	0.834	N.N.	N.N.	PP429385	PP429445
MLR_8	A	0.437	N.N.	N.N.	PP429386	PP429446
MLR_9	A	N.B.	N.N.	N.N.	PP429387	PP429447
MLR_10	A	N.B.	N.N.	N.N.	PP429388	PP429448
MLR_11	A	N.B.	N.N.	N.N.	PP429389	PP429449
MLR_12	A	N.B.	N.N.	N.N.	PP429390	PP429450
MLR_13	A	N.B.	N.N.	N.N.	PP429391	PP429451
MLR_14	B	N.B.	N.N.	N.N.	PP429392	PP429452
MLR_15	B	1.017	130.57	21.75	PP429393	PP429453
MLR_16	B	0.333	N.N.	N.N.	PP429394	PP429454
MLR_17	B	N.B.	N.N.	N.N.	PP429395	PP429455
MLR_18	B	N.B.	N.N.	N.N.	PP429396	PP429456
MLR_19	B	0.465	N.N.	N.N.	PP429397	PP429457
MLR_20	B	N.B.	N.N.	N.N.	PP429398	PP429458
MLR_21	B	N.B.	N.N.	N.N.	PP429399	PP429459
MLR_22	B	0.303	N.N.	N.N.	PP429400	PP429460
MLR_23	B	N.B.	N.N.	N.N.	PP429401	PP429461
MLR_24	B	1.122	24.2	0.22	PP429402	PP429462
MLR_25	B	N.B.	N.N.	N.N.	PP429403	PP429463
MLR_26	B	N.B.	N.N.	N.N.	PP429404	PP429464
MLR_27	B	0.494	N.N.	N.N.	PP429405	PP429465
MLR_28	B	N.B.	N.N.	N.N.	PP429406	PP429466
MLR_29	B	N.B.	N.N.	N.N.	PP429407	PP429467
MLR_30	B	0.776	N.N.	N.N.	PP429408	PP429468
MLR_31	B	N.B.	N.N.	N.N.	PP429409	PP429469
MLR_32	B	N.B.	N.N.	N.N.	PP429410	PP429470
MLR_33	B	0.753	N.N.	N.N.	PP429411	PP429471
MLR_34	C	N.B.	N.N.	N.N.	PP429412	PP429472
MLR_35	C	N.B.	N.N.	N.N.	PP429413	PP429473
MLR_36	C	N.B.	N.N.	N.N.	PP429414	PP429474
MLR_37	C	N.B.	N.N.	N.N.	PP429415	PP429475
MLR_38	C	N.B.	N.N.	N.N.	PP429416	PP429476
MLR_39	C	N.B.	N.N.	N.N.	PP429417	PP429477
MLR_40	C	N.B.	N.N.	N.N.	PP429418	PP429478
MLR_41	C	N.B.	N.N.	N.N.	PP429419	PP429479
MLR_42	C	0.362	N.N.	N.N.	PP429420	PP429480
MLR_43	C	N.B.	N.N.	N.N.	PP429421	PP429481
MLR_44	C	N.B.	N.N.	N.N.	PP429422	PP429482
MLR_45	C	0.882	N.N.	N.N.	PP429423	PP429483
MLR_46	C	N.B.	N.N.	N.N.	PP429424	PP429484
MLR_47	C	N.B.	N.N.	N.N.	PP429425	PP429485
MLR_48	C	N.B.	N.N.	N.N.	PP429426	PP429486
MLR_49	C	N.B.	N.N.	N.N.	PP429427	PP429487
MLR_50	C	N.B.	N.N.	N.N.	PP429428	PP429488
MLR_51	C	0.500	N.N.	N.N.	PP429429	PP429489

MLR_52	C	N.B.	N.N.	N.N.	PP429430	PP429490
MLR_53	C	0.381	N.N.	N.N.	PP429431	PP429491
MLR_54	C	0.430	N.N.	N.N.	PP429432	PP429492
MLR_55	C	0.606	N.N.	98.21	PP429433	PP429493
MLR_56	C	N.B.	N.N.	N.N.	PP429434	PP429494
MLR_57	C	N.B.	N.N.	N.N.	PP429435	PP429495
MLR_58	C	N.B.	N.N.	N.N.	PP429436	PP429496
MLR_59	C	N.B.	N.N.	N.N.	PP429437	PP429497
MLR_60	C	0.426	N.N.	N.N.	PP429438	PP429498
*N.B., non-binder; **N.N. non-neutralizing.						

Table S2: Properties and accession numbers for sorted and produced VH3-21/VL1-40 mAbs

Supplemental References

1. Goodwin, E., Gilman, M.S.A., Wrapp, D., Chen, M., Ngwuta, J.O., Moin, S.M., Bai, P., Sivasubramanian, A., Connor, R.I., Wright, P.F., et al. (2018). Infants Infected with Respiratory Syncytial Virus Generate Potent Neutralizing Antibodies that Lack Somatic Hypermutation. Immunity *48*, 339-349.e335. 10.1016/j.immuni.2018.01.005.
2. Fong, Y., Huang, Y., Gilbert, P.B., and Permar, S.R. (2017). chngpt: threshold regression model estimation and inference. BMC Bioinformatics *18*, 454. 10.1186/s12859-017-1863-x.
3. McLellan, J.S., Chen, M., Joyce, M.G., Sastry, M., Stewart-Jones, G.B., Yang, Y., Zhang, B., Chen, L., Srivatsan, S., Zheng, A., et al. (2013). Structure-based design of a fusion glycoprotein vaccine for respiratory syncytial virus. Science *342*, 592-598. 10.1126/science.1243283.
4. Wu, X., Yang, Z.Y., Li, Y., Hogerkorp, C.M., Schief, W.R., Seaman, M.S., Zhou, T., Schmidt, S.D., Wu, L., Xu, L., et al. (2010). Rational design of envelope identifies broadly neutralizing human monoclonal antibodies to HIV-1. Science *329*, 856-861. 10.1126/science.1187659.
